# Supplementary material for: Metabolic mutations reduce antibiotic susceptibility of E. coli by pathway-specific bottlenecks
Source: Mol Syst Biol. 2025 Jan 2;21(3):274–93. doi: 10.1038/s44320-024-00084-z (PMC11876631; doi:10.1038/s44320-024-00084-z)
Supplement: Supplementary file 1 — Appendix [file 44320_2024_84_MOESM1_ESM.pdf]

Appendix for

Metabolic mutations reduce antibiotic susceptibility of *E. coli* by pathway-specific bottlenecks

Paul Lubrano[1,2,3,#], Fabian Smollich[1,2,3,#], Thorben Schramm[1†], Elisabeth Lorenz[1], Alejandra Alvarado[1,2], Seraina Carmen Eigenmann[6], Amelie Stadelmann[1,2,3], Sevvalli Thavapalan[1,2,3], Nils Waffenschmidt[1], Timo Glatter[4], Nadine Hoffmann[2,5], Jennifer Müller[5,7], Silke Peter[2,5,7], Knut Drescher[6], Hannes Link[1,2,3]*

[1]Interfaculty Institute of Microbiology and Infection Medicine, University of Tübingen, Auf der Morgenstelle 24, 72076 Tübingen, Germany

[2]Cluster of Excellence "Controlling Microbes to Fight Infections", University of Tübingen, 72076 Tübingen, Germany

[3]M3 Research Center, Otfried-Müller-Straße 37, University of Tübingen, 72076 Tübingen, Germany

[4]Max Planck Institute for Terrestrial Microbiology, Karl-von-Frisch-Straße 10, 35043 Marburg, Germany

[5]Institute of Medical Microbiology and Hygiene, University of Tübingen, Elfriede-Aulhorn-Str. 6, 72076 Tübingen

[6]Biozentrum, University of Basel, Spitalstrasse 41, 4056 Basel, Switzerland

[7]NGS Competence Center Tübingen (NCCT), 72076 Tübingen, Germany

†Present address: Institute of Molecular Systems Biology, ETH Zurich, Otto-Stern-Weg 3, 8093 Zürich, Switzerland

[#]Equal contribution

* Corresponding author: hannes.link@uni-tuebingen.de

Content

Page 3: Appendix Figure S1 – Comparison of CFU per mL on different concentrations of carbenicillin and gentamicin.

Page 4: Appendix Figure S2 – Images of M9 agar plates inoculated with the control strain (top) or the CRISPR library (bottom).

Page 5: Appendix Figure S3 – Agar dilution assays with the control strain and CRISPR mutants (RibD^{L364W}, PgsA^{V44P}, IspE^{V146W}, PtsI^{I330P}, HemA^{L276Q}, PurA^{L75D}, PurM^{F105A} and HisF^{V126P}).

Page 6: Appendix Figure S4 - Agar dilution assays with the control strain and CRISPR mutants (PurA^{L75D}, HemA^{L276Q}) in the absence of chloramphenicol and kanamycin.

Page 7: Appendix Figure S5 – Transcriptome changes in the PurAL75D strain relative to the control strain.

Page 8: Appendix Figure S6 – Comparison of the metabolome of the RibDL364W mutant and the control strain.

Page 9: Appendix Figure S7 – Knockdown of purA with CRISPR interference

Page 10: Appendix Figure S8 – Relative levels of intracellular ATP, ADP, AMP and IMP in the Control and PurA^{L75D} strain in minimal glucose medium with or without supplementation of 1 mM adenine.

Page 11: Appendix Figure S9 – Agar dilution assay with the control strain, the PurA^{L75D} strain and the slow-growth control LeuB^{I134P}.

Page 12: Appendix Figure S10 - Agar dilution assays with the control strain and the mutants PtsI^{I330P}, HemA^{L276Q}, PurA^{L75D}, PurM^{F105A} and HisF^{V126P}.

Page 13: Appendix Figure S11 – Time-kill assays with the control strain and the LeuB^{I134P}, HisF^{V126P}, PurM^{F105A} and PurA^{L75D} strains.

Page 14: Appendix Figure S12 – Relative levels of ATP, ADP and AMP in EC-244 (with and without adenine) and EC-249 (without adenine).

Page 15: Appendix Figure S13 – Distribution of the frequency of 146 amino-acid mutations in PurK found in the NCBI pathogen database (4352 *E. coli* strains).

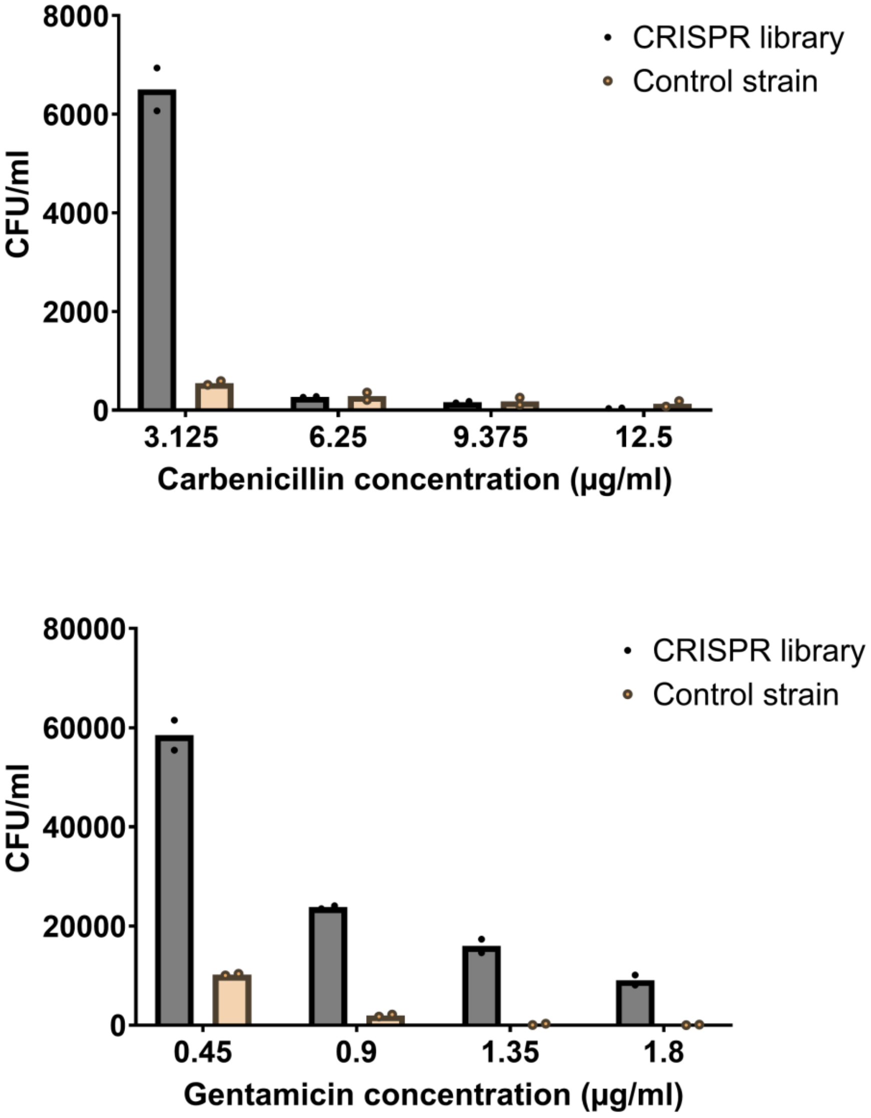

Appendix Figure S1 – Comparison of CFU per mL on different concentrations of carbenicillin and gentamicin.

The CRISPR library and control strain were plated on minimal agar medium with different concentrations of carbenicillin and gentamicin, corresponding to 2X, 4X, 6X and 8X MIC of the control strain. Duplicates of each strain were tested. Colonies were counted after 48 h of incubation at 37°C to determine CFU per mL.

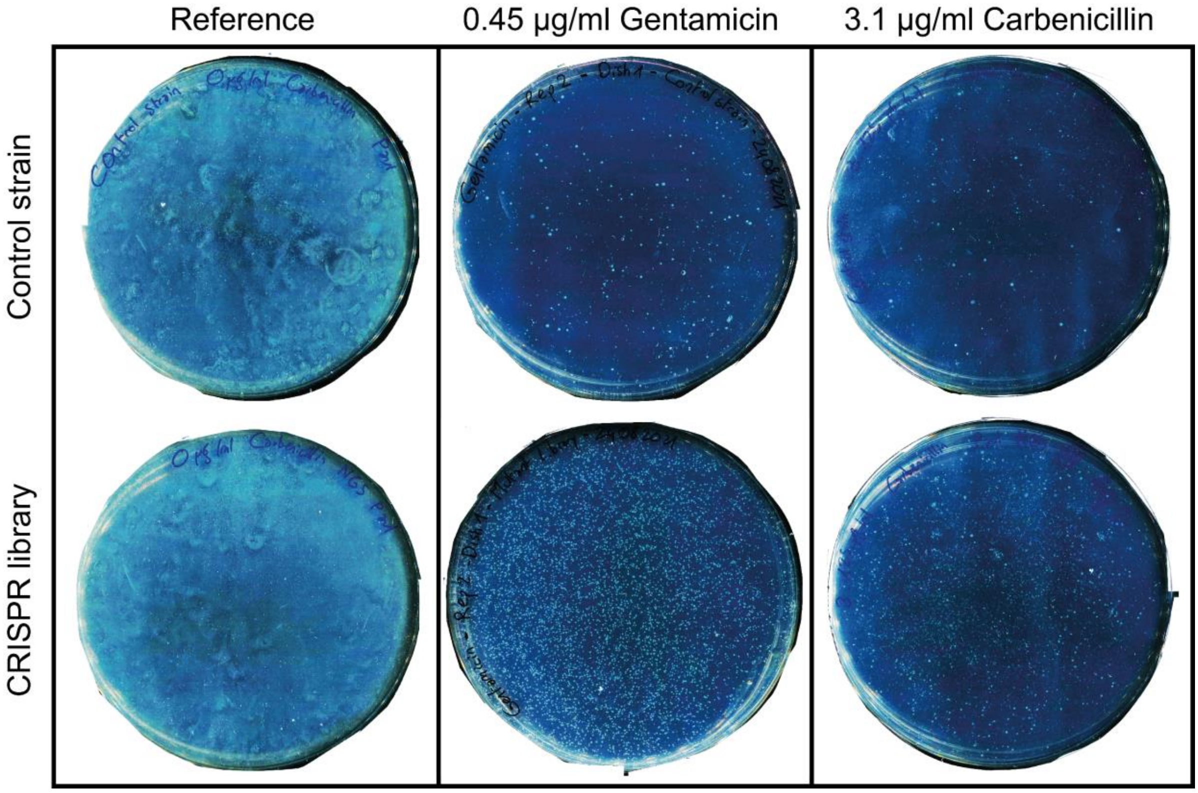

Appendix Figure S2 – Images of M9 agar plates inoculated with the control strain (top) or the CRISPR library (bottom).

Plates contained either gentamicin, carbenicillin, or no additional antibiotics (reference). Images were made with an Epson V370 scanner. Images were assembled using Adobe Illustrator. Brightness and contrast were adjusted with Microsoft PowerPoint.

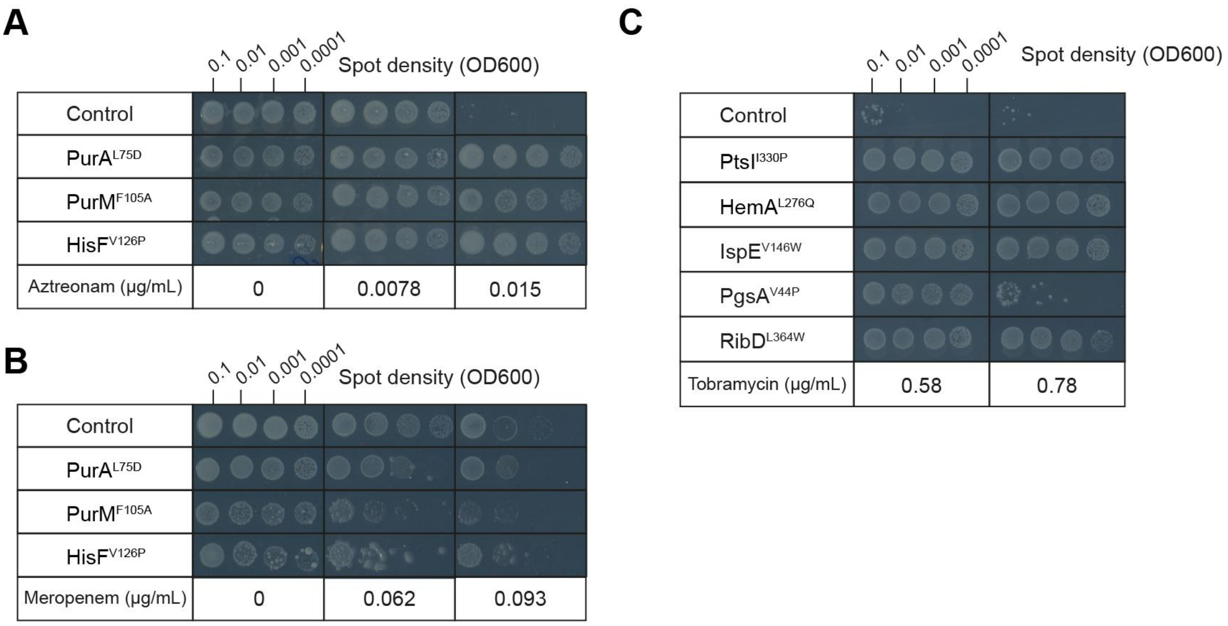

Appendix Figure S3 – Agar dilution assays with the control strain and CRISPR mutants (RibD^{L364W}, PgsA^{V44P}, IspE^{V146W}, PtsI^{I330P}, HemA^{L276Q}, PurA^{L75D}, PurM^{F105A} and HisF^{V126P}).

Strains were plated on minimal agar medium supplemented with the respective antibiotic of the β-lactam class aztreonam (A) or meropenem (B), or the aminoglycoside tobramycin (C). Multiple inoculum densities were used to assess inoculum effects. Plates were incubated 48 h. Shown are one of n = 2 replicates. Spot assays were performed on the same plate per concentration, and scans of plates with different concentrations were assembled into a single figure using Adobe Illustrator. Drug-free spots of RibD^{L364W}, PgsA^{V44P}, IspE^{V146W}, PtsI^{I330P}, HemA^{L276Q} are shown in Figure 2D of the main text.

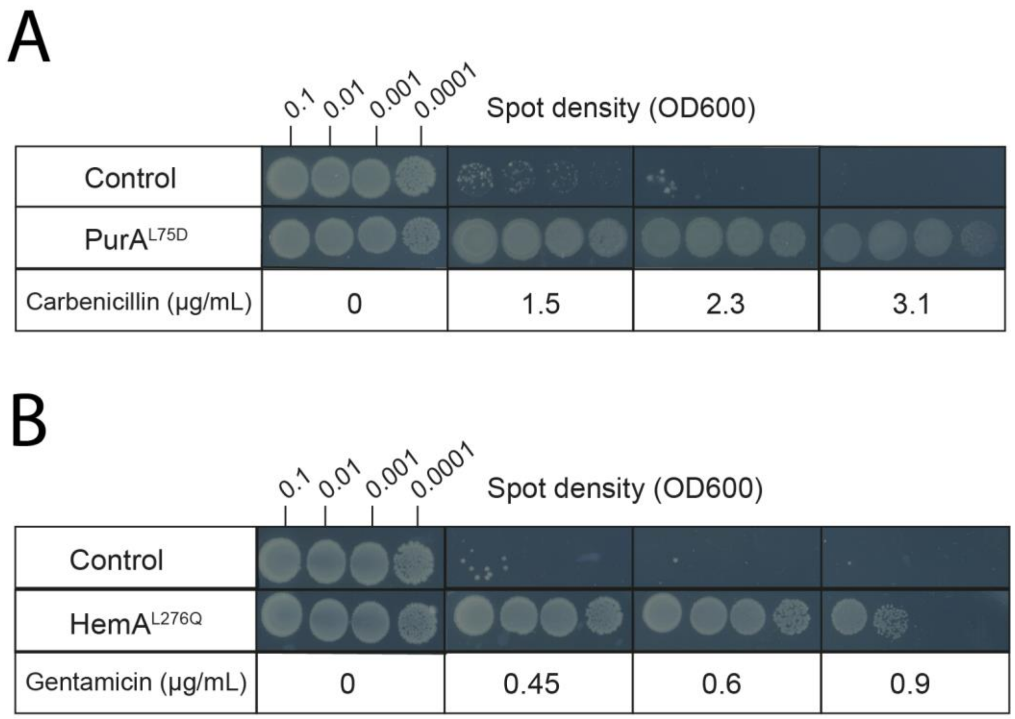

Appendix Figure S4 – Agar dilution assays with the control strain and CRISPR mutants (PurA^{L75D}, HemA^{L276Q}) in the absence of chloramphenicol and kanamycin.

Strains were plated on minimal agar medium supplemented with either carbenicillin (A) or gentamicin (B). In contrast to other spot assays, the plates did not contain the selection markers chloramphenicol and kanamycin. Cells were washed twice with M9 before spotting to remove remaining antibiotics in the growth medium. Multiple inoculum densities were used to assess inoculum effects. Plates were incubated 48 h. Shown are one of n = 2 replicates. Spot assays were performed on the same plate per concentration, and scans of plates with different concentrations were assembled into a single figure using Adobe Illustrator.

A

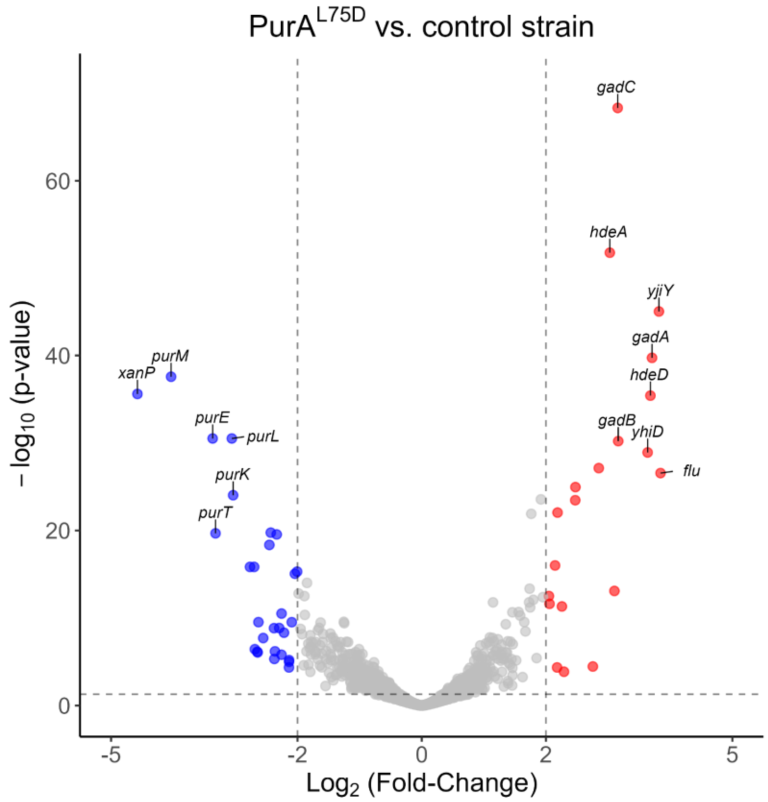

B

Gene	GO Terms	Description	Function
flu	N/A	CP4-44 prophage; self recognizing antigen 43 (Ag43) autotransporter	N/A
gadA	GO:0051454<br>GO:0006536<br>GO:0006538	Glutamate decarboxylase A	Acid resistance
gadB	GO:0051454<br>GO:0006536<br>GO:0006538	Glutamate decarboxylase B	Acid resistance
gadC	GO:0051454<br>GO:0006865<br>GO:0055085	L-glutamate:4-aminobutyrate antiporter	Acid resistance
hdeA	GO:0061077<br>GO:1990451<br>GO:0071468	Periplasmic acid stress chaperone	Acid resistance
hdeD	GO:0009268	Acid-resistance membrane protein	Acid resistance
yhiD	N/A	Inner membrane protein	N/A
yjiY	GO:0006849<br>GO:0006974<br>GO:0031669<br>GO:0009267	Pyruvate:H^+ symporter	Pyruvate uptake

Appendix Figure S5 – Transcriptome changes in the PurA^{L75D} strain relative to the control strain.

A) Volcano plot shows the $\log_2$ fold-change on the x axis and the $-\log_{10}$ of the adjusted p values on the y axis. A subset of significant genes with a fold change >2 or <-2 and a p value <0.05 is annotated. Both strains were measured with n = 3 replicates. Figure was created using R and the package ggplot2 (version 3.4.4). B) Eight genes that are upregulated in the PurA^{L75D} strain, their GO terms, name of the gene product, and the general function.

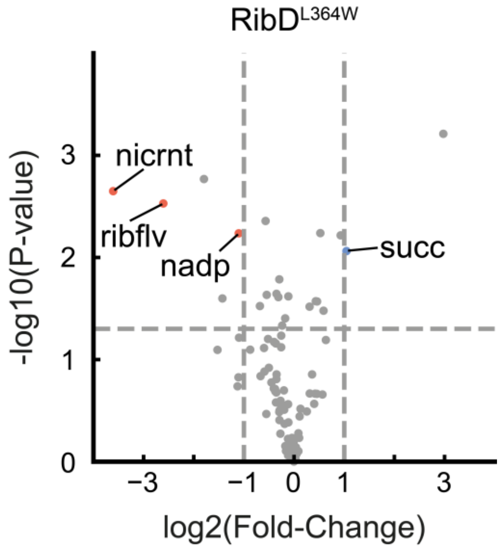

Appendix Figure S6 – Comparison of the metabolome of the RibD^{L364W} mutant and the control strain.

Volcano plot showing metabolite levels of the RibD^{L364W} mutant relative to the control strain (n=3 distinct samples). Significant metabolites of interest are annotated (p-value < 0.05).

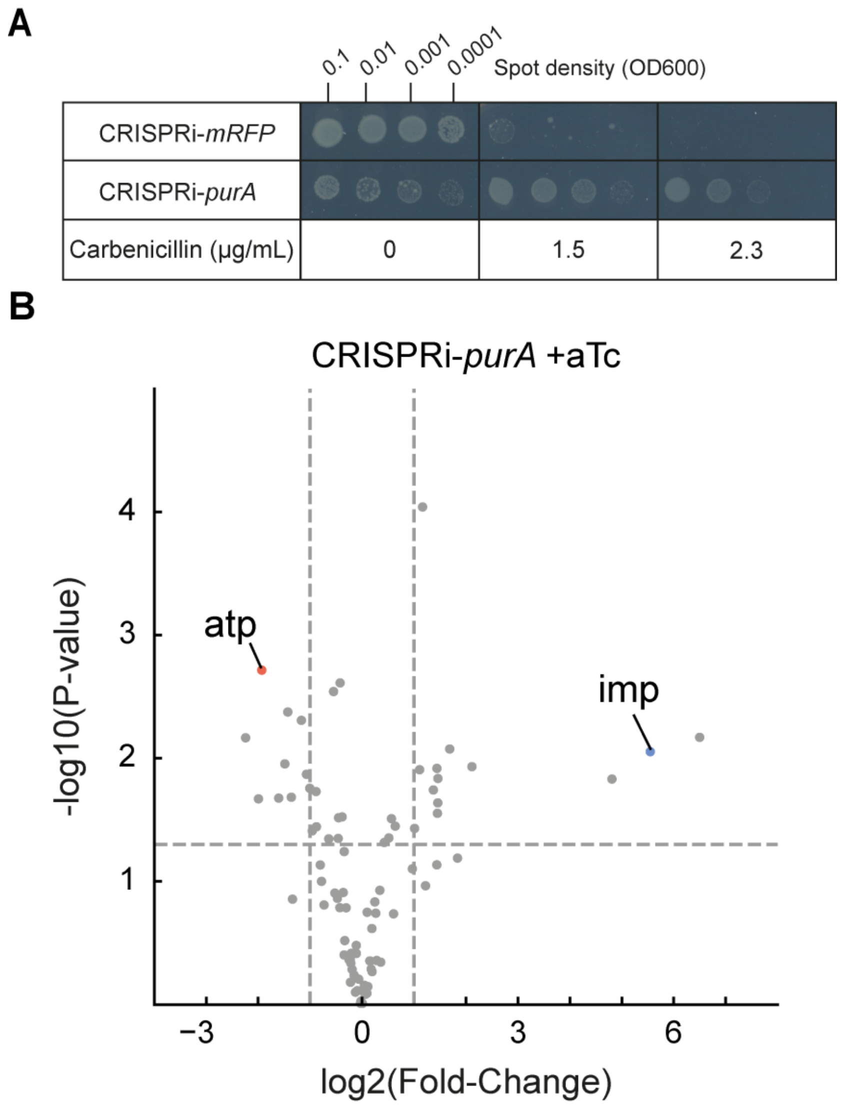

Appendix Figure S7 – Knockdown of *purA* with CRISPR interference.

A. Agar dilution assay with the CRISPRi-*purA* strain and the CRISPRi-*mRFP* strain (control). Each strain was plated on agar plates with minimal glucose medium containing increasing concentrations of carbenicillin (MIC = 1.5 µg/mL). 1 µM anhydrotetracycline (aTc) was added to induce the expression of dCas9. Multiple inoculum densities were used to assess inoculum effects. Plates were incubated 48 h. Shown is one of n = 2 replicates. Spot assays were performed on the same plate per concentration, and scans of plates with different concentrations were assembled into a single figure using Adobe Illustrator. **B.** Volcano plot showing metabolite levels of the aTc-induced CRISPRi-*purA* strain relative to the non-induced CRISPRi-*purA* strain (n=3 distinct samples). Significant metabolites of interest are annotated (p-value < 0.05).

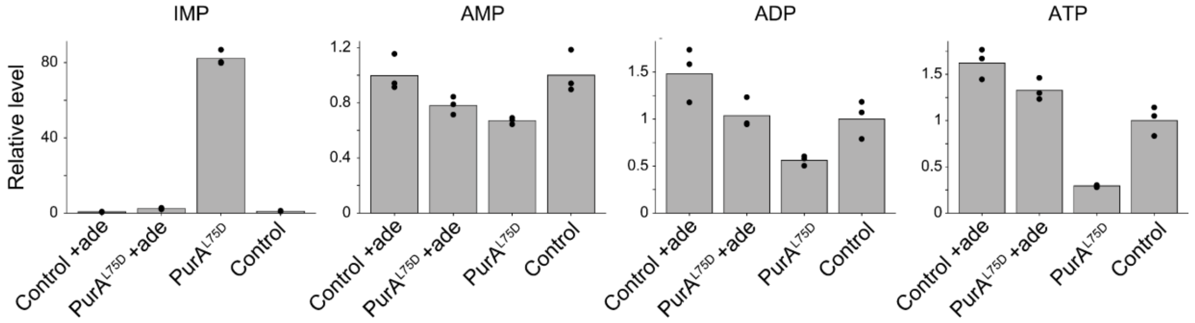

Appendix Figure S8 – Relative levels of intracellular ATP, ADP, AMP and IMP in the Control and PurA^{L75D} strain in minimal glucose medium with or without supplementation of 1 mM adenine.

Data are normalized to the control strain without adenine (ade). Bars are means of n = 3 distinct samples (black dots). Data for the control strain and the PurA^{L75D} mutant without adenine feeding is the same as shown in Fig. 2E.

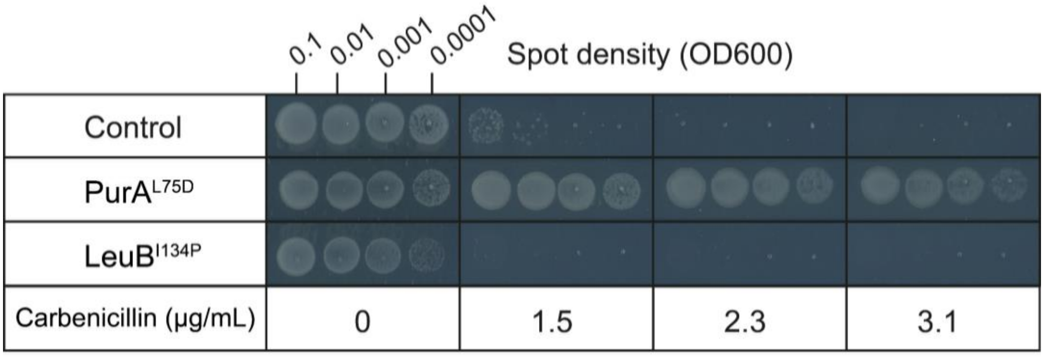

Appendix Figure S9 – Agar dilution assay with the control strain, the PurA^{L75D} strain and the slow-growth control LeuB^{I134P}.

Plates were incubated 48 h. Spot assays were performed on the same plate per concentration, and scans of plates with different concentrations were assembled into a single figure using Adobe Illustrator. Shown is one of n=2 replicates.

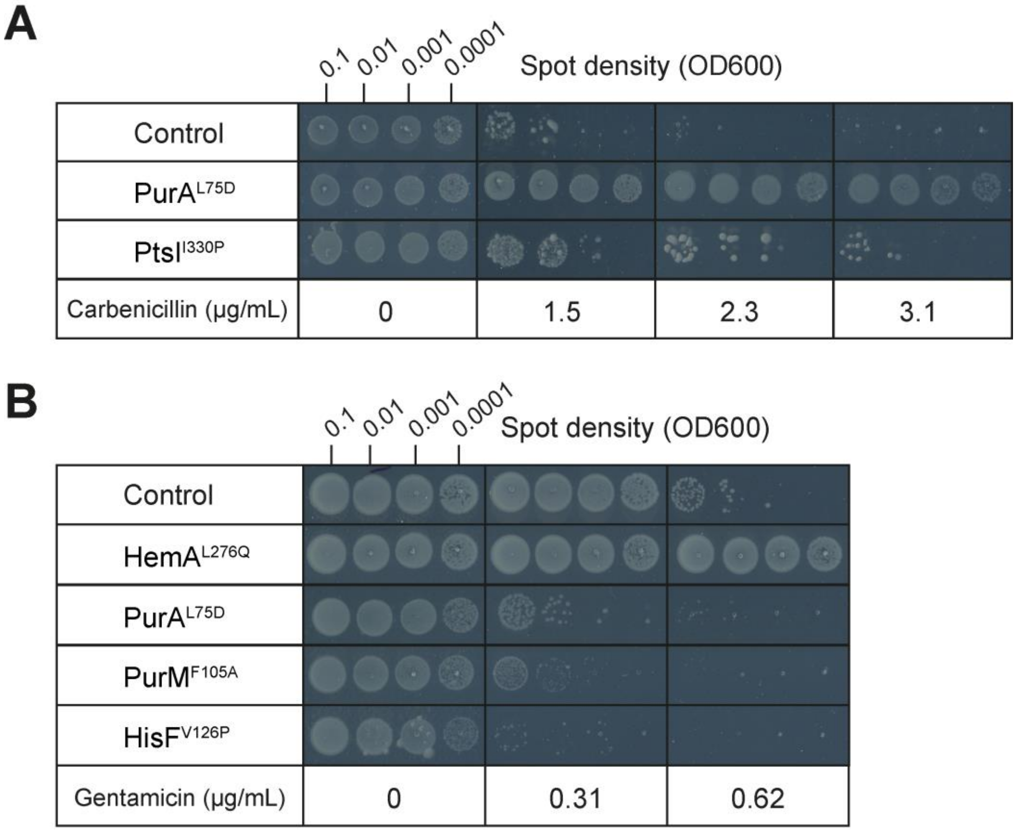

Appendix Figure S10 - Agar dilution assays with the control strain and the mutants PtsI^{I330P}, HemA^{L276Q}, PurA^{L75D}, PurM^{F105A} and HisF^{V126P}.

Strains were plated on minimal agar medium supplemented either with carbenicillin (A) or with gentamicin (B). Spot assays were performed on the same plate per concentration, and scans of plates with different concentrations were assembled into a single figure using Adobe Illustrator.

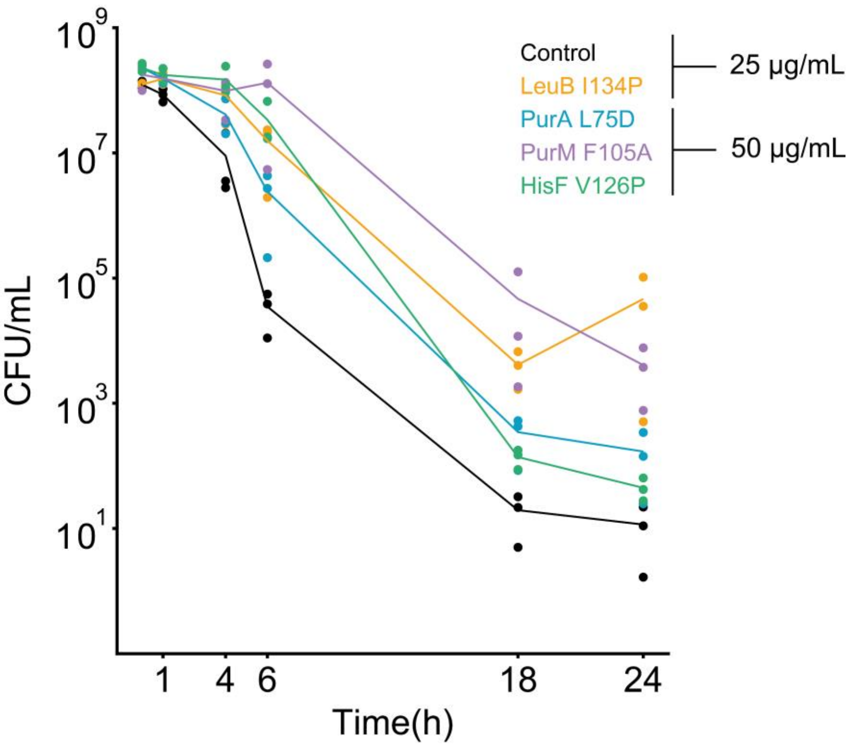

Appendix Figure S11 – Time-kill assays with the control strain and the LeuB^{I134P}, HisF^{V126P}, PurM^{F105A} and PurA^{L75D} strains.

Strains were incubated in minimal glucose medium and carbenicillin (25 and 50 µg/mL) for the time period indicated on the x-axis (n=3 distinct samples). Data for the HisF^{V126P}, PurM^{F105A} and PurA^{L75D} mutants is the same as in Fig. 3c and shown as a reference.

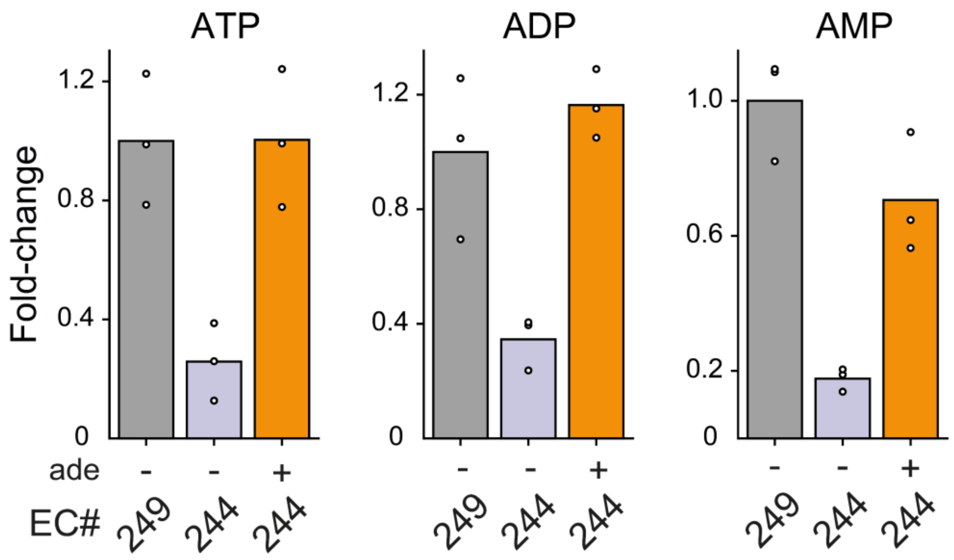

Appendix Figure S12 – Relative levels of ATP, ADP and AMP in EC-244 (with and without adenine) and EC-249 (without adenine).

Data are normalized to EC-249 (without adenine) and are represented as mean of n = 3 distinct samples (shown as black dots).

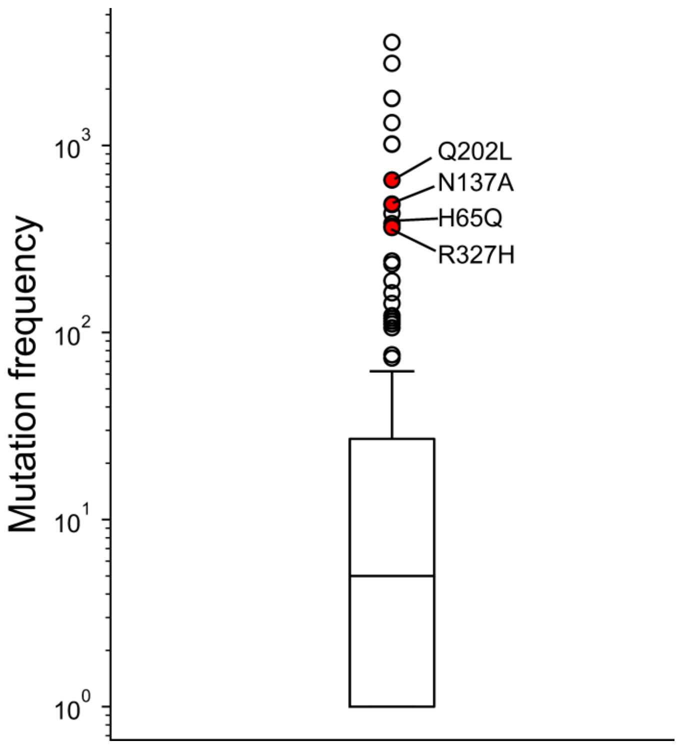

Appendix Figure S13 – Distribution of the frequency of 146 amino-acid mutations in PurK found in the NCBI pathogen database (4352 *E. coli* strains).

Mutations were found by alignment with the *purK* gene of *E. coli* BW25113. Each dot represents an amino-acid mutation, and its frequency in various isolates is indicated on the y-axis. Mutations detected in EC-244 and EC-249 are annotated, except for E49G which was not present in the dataset.
