# Supplementary figures and images for: Metabolic mutations reduce antibiotic susceptibility of E. coli by pathway-specific bottlenecks
Source: Mol Syst Biol. 2025 Jan 2;21(3):274–93. doi: 10.1038/s44320-024-00084-z (PMC11876631; doi:10.1038/s44320-024-00084-z)

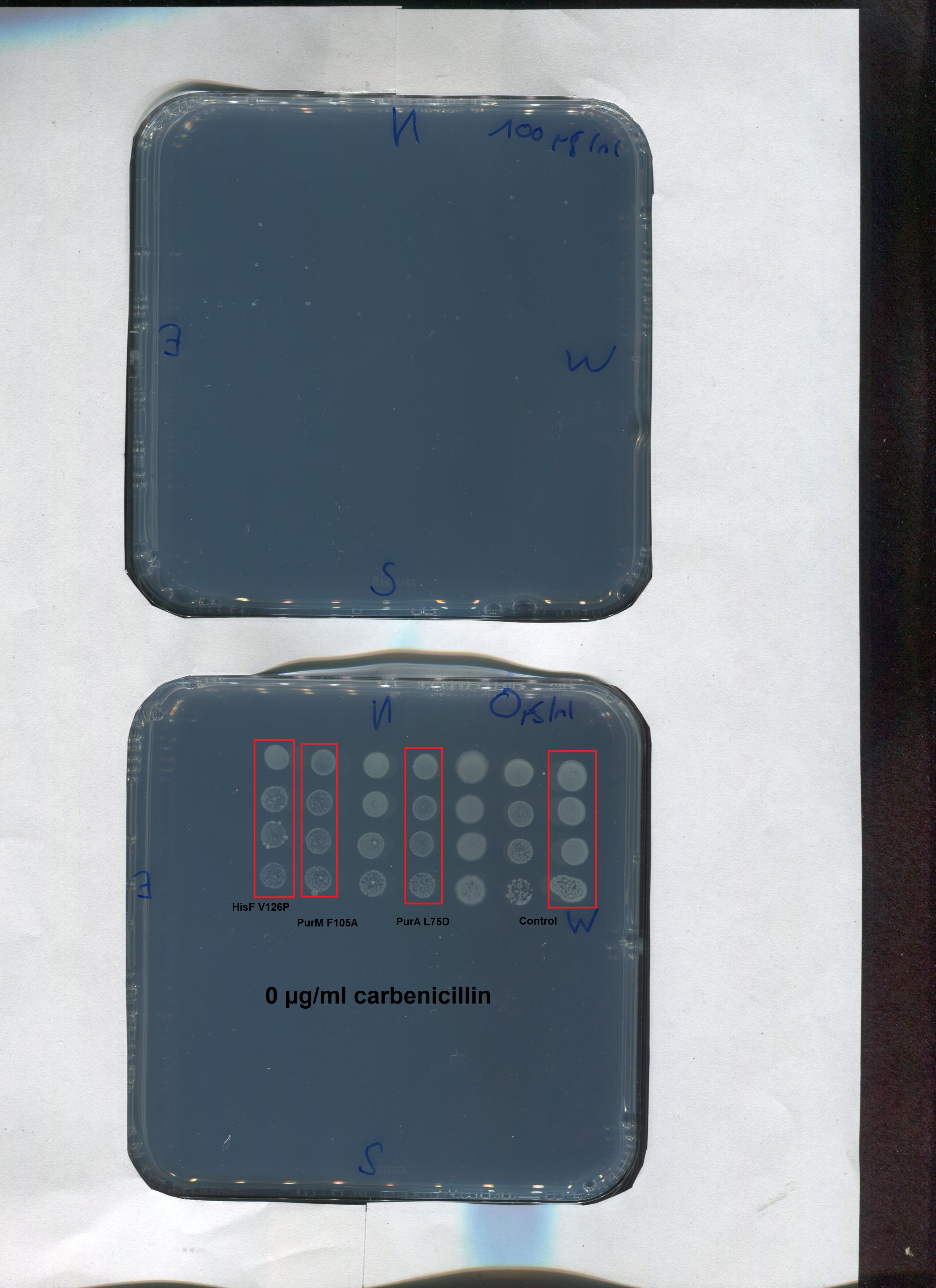

Supplement: Supplementary file 12 — Source data Fig. 2 [file 44320_2024_84_MOESM12_ESM.zip › SD_fig2/2B/0 and 100.jpg]

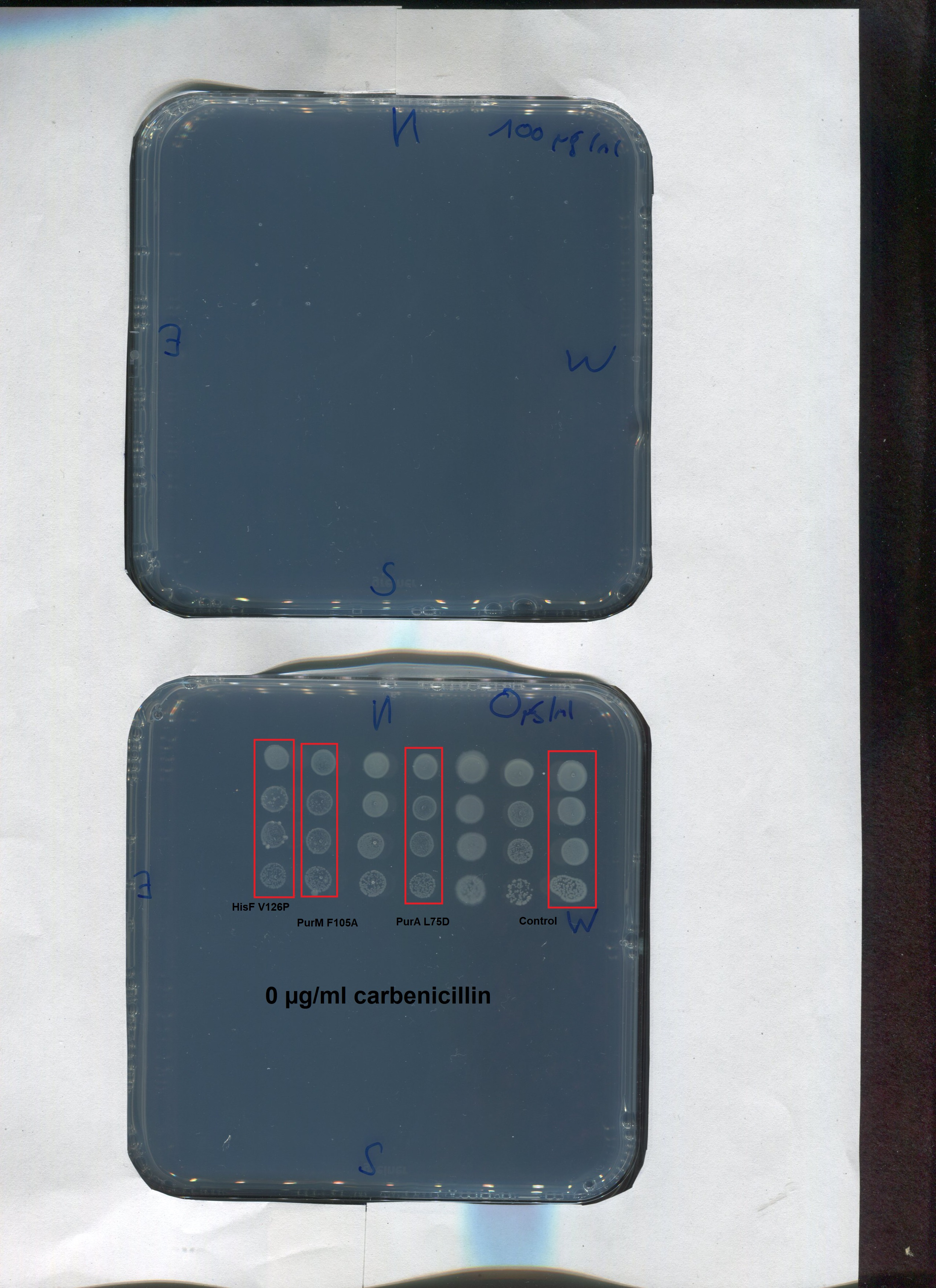

Supplement: Supplementary file 12 — Source data Fig. 2 [file 44320_2024_84_MOESM12_ESM.zip › SD_fig2/2B/0 and 100.tif]

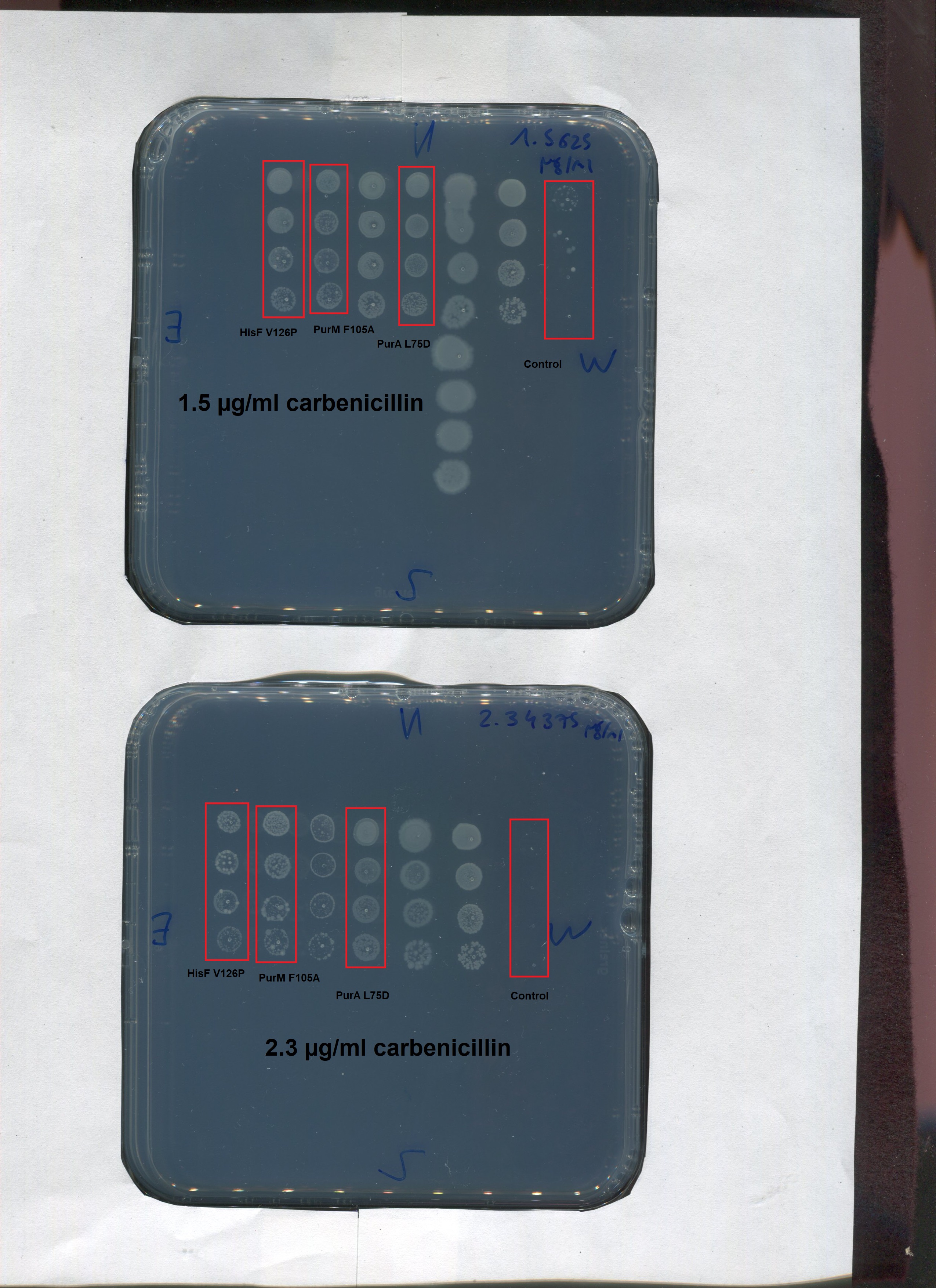

Supplement: Supplementary file 12 — Source data Fig. 2 [file 44320_2024_84_MOESM12_ESM.zip › SD_fig2/2B/1.5_and_2.3.jpg]

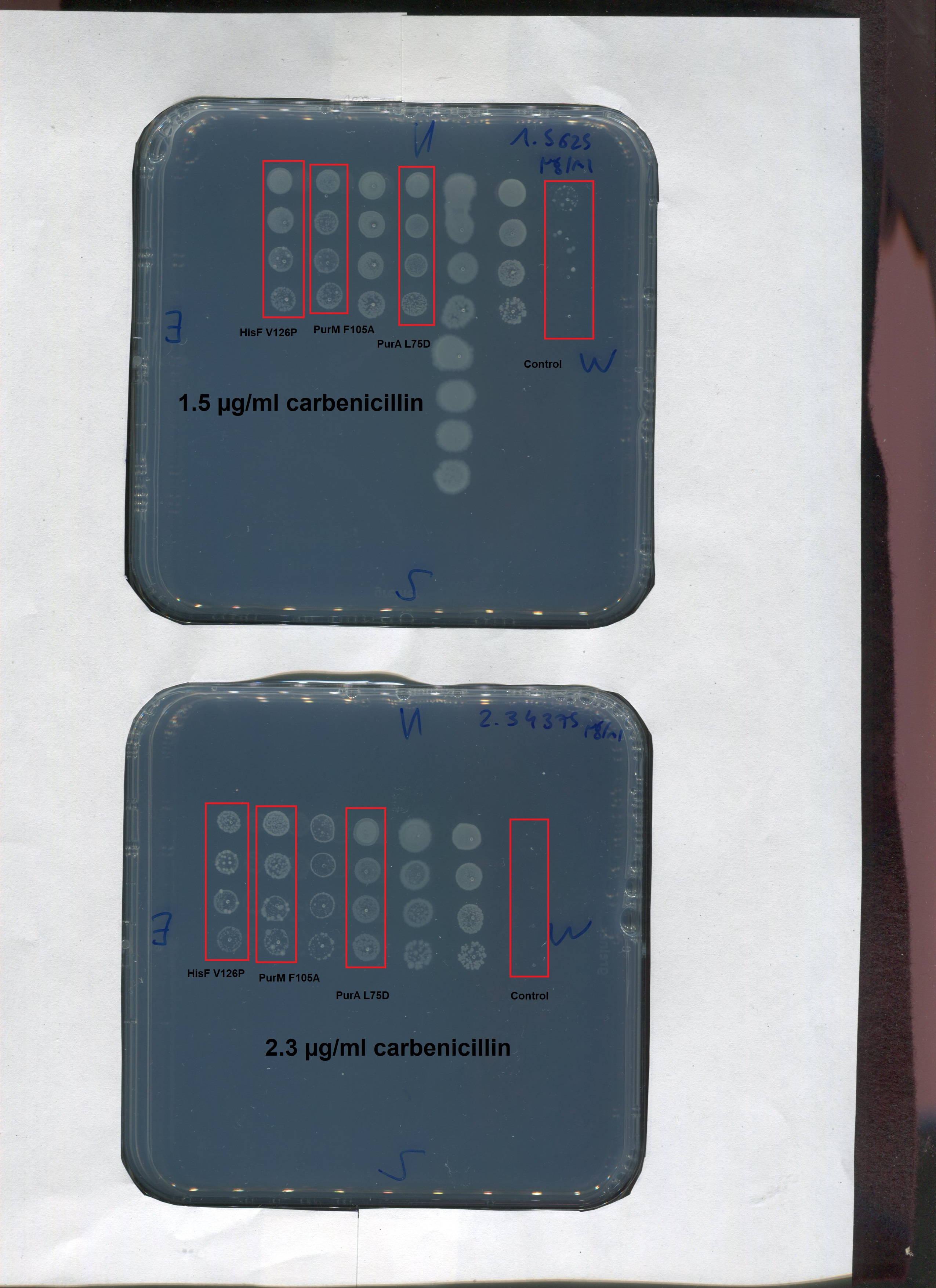

Supplement: Supplementary file 12 — Source data Fig. 2 [file 44320_2024_84_MOESM12_ESM.zip › SD_fig2/2B/1.5_and_2.3.tif]

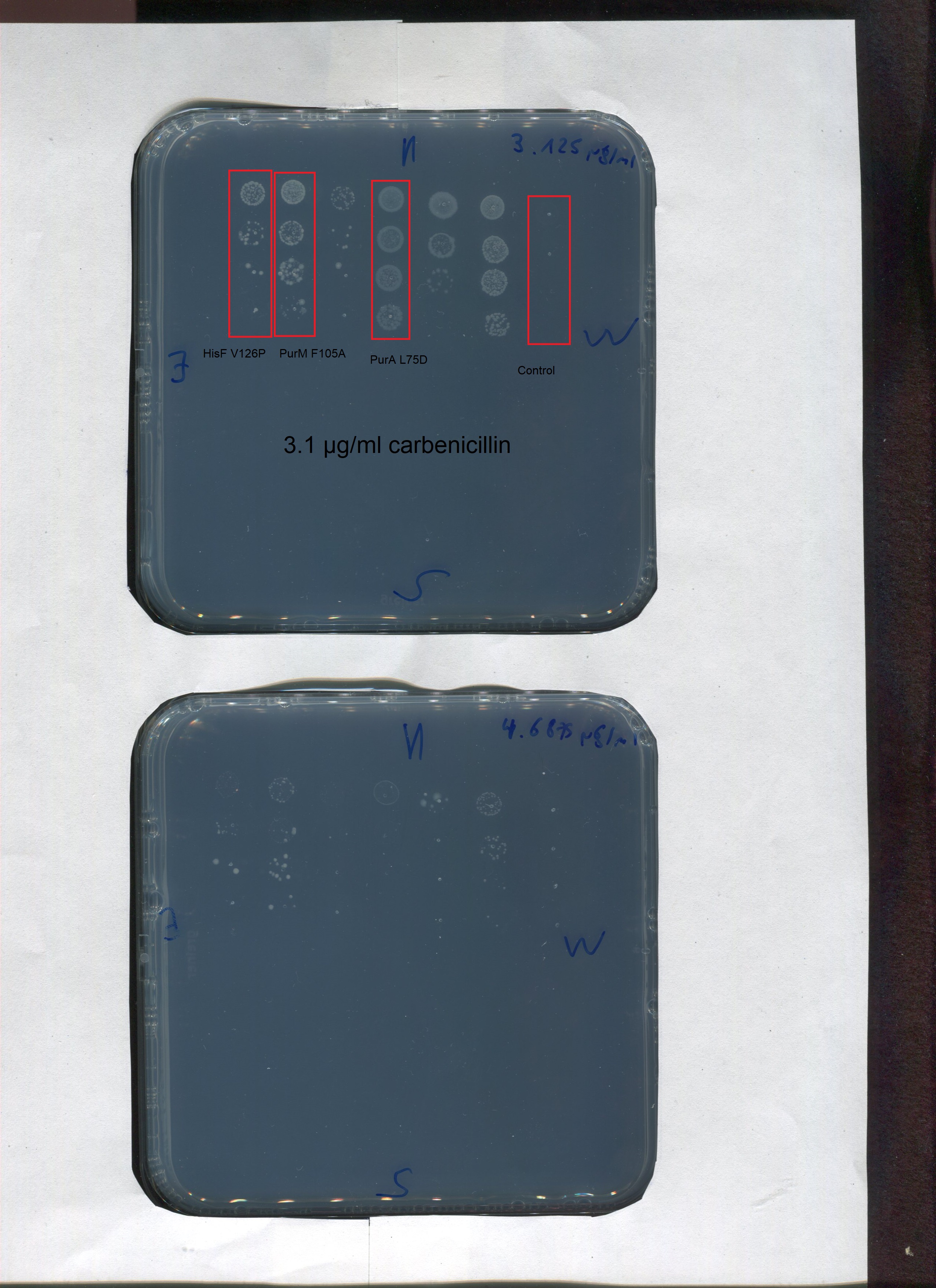

Supplement: Supplementary file 12 — Source data Fig. 2 [file 44320_2024_84_MOESM12_ESM.zip › SD_fig2/2B/3.1_and_4.6.jpg]

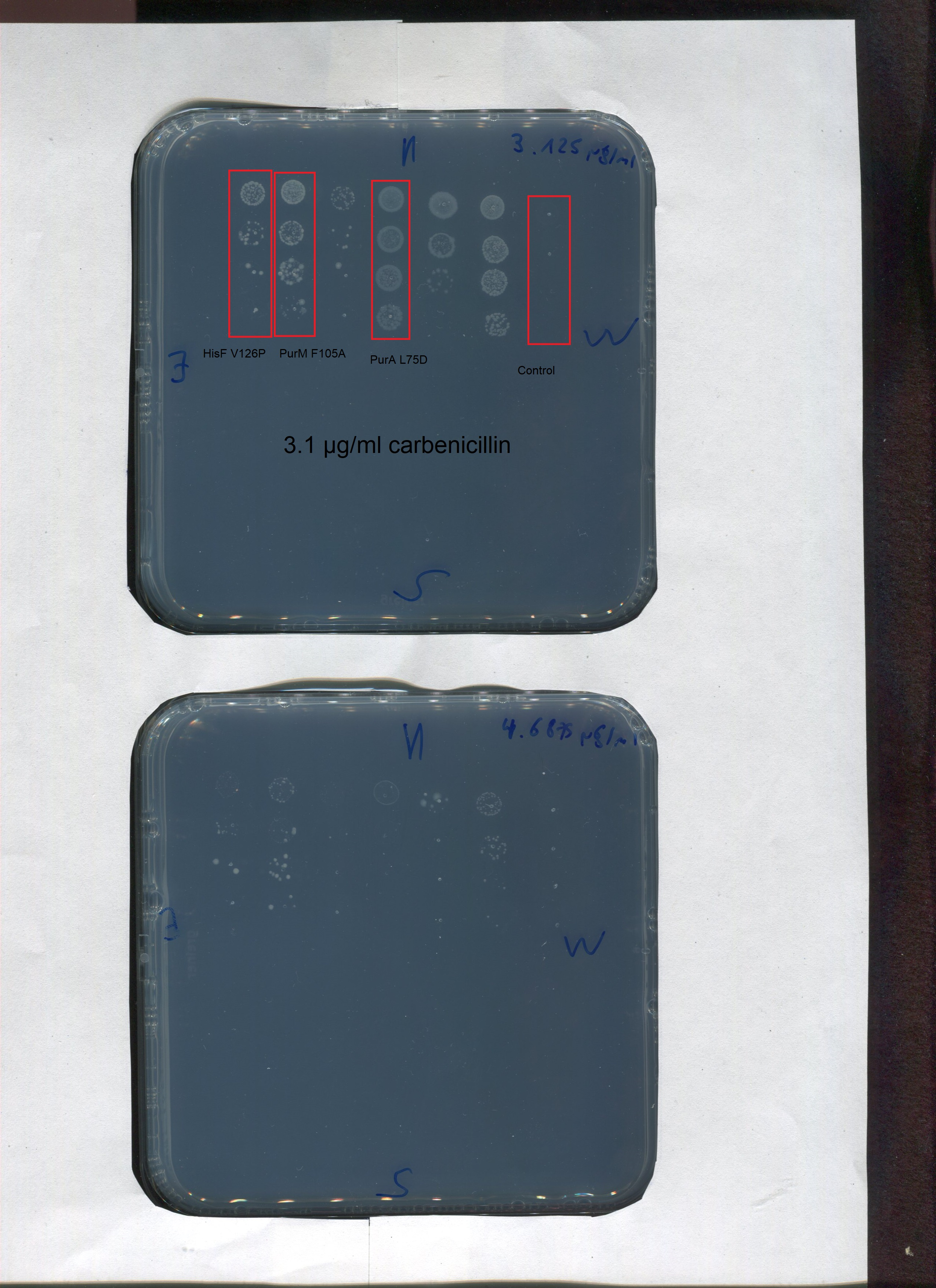

Supplement: Supplementary file 12 — Source data Fig. 2 [file 44320_2024_84_MOESM12_ESM.zip › SD_fig2/2B/3.1_and_4.6.tif]

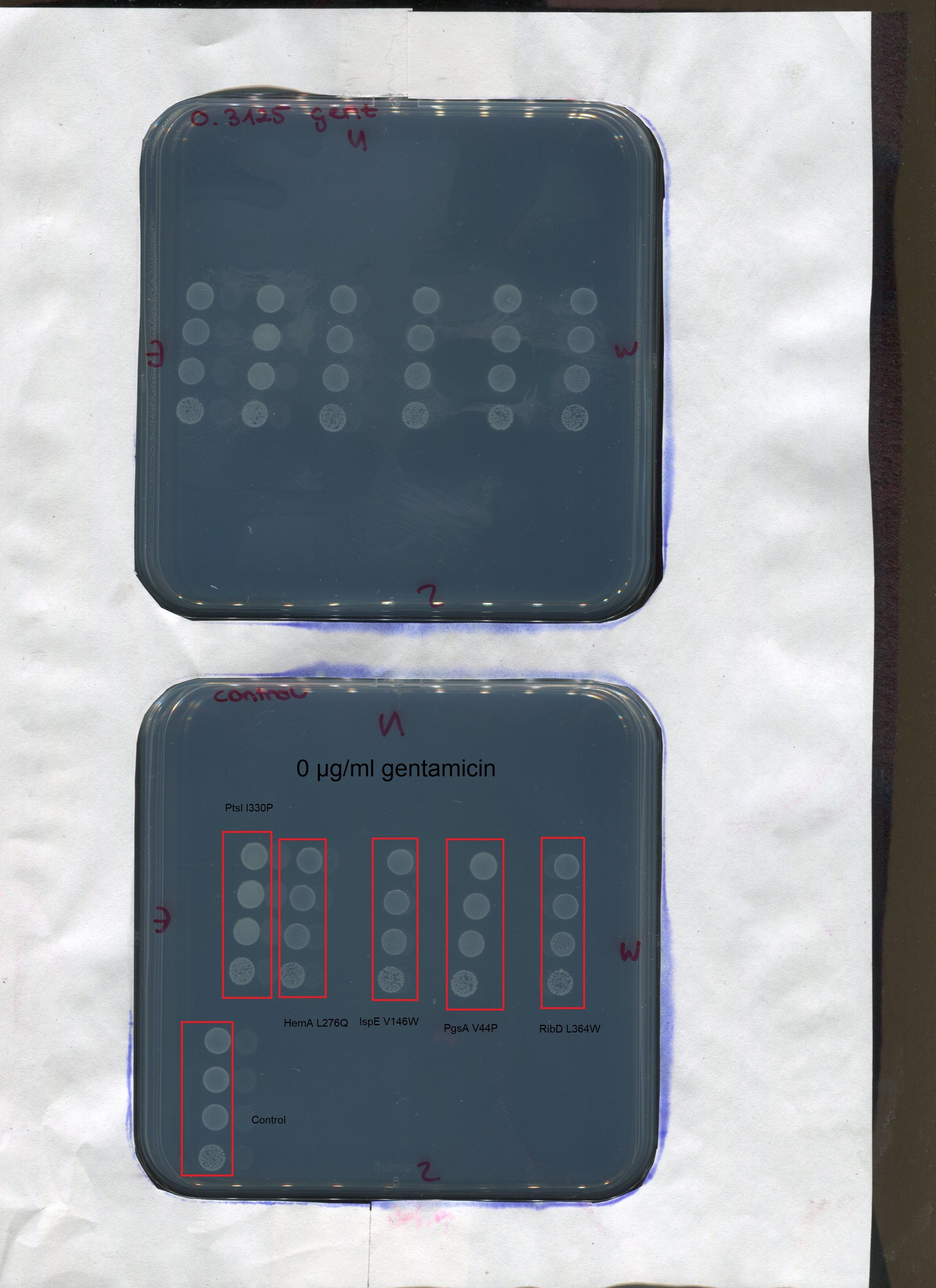

Supplement: Supplementary file 12 — Source data Fig. 2 [file 44320_2024_84_MOESM12_ESM.zip › SD_fig2/2D/0.3 and 0.jpg]

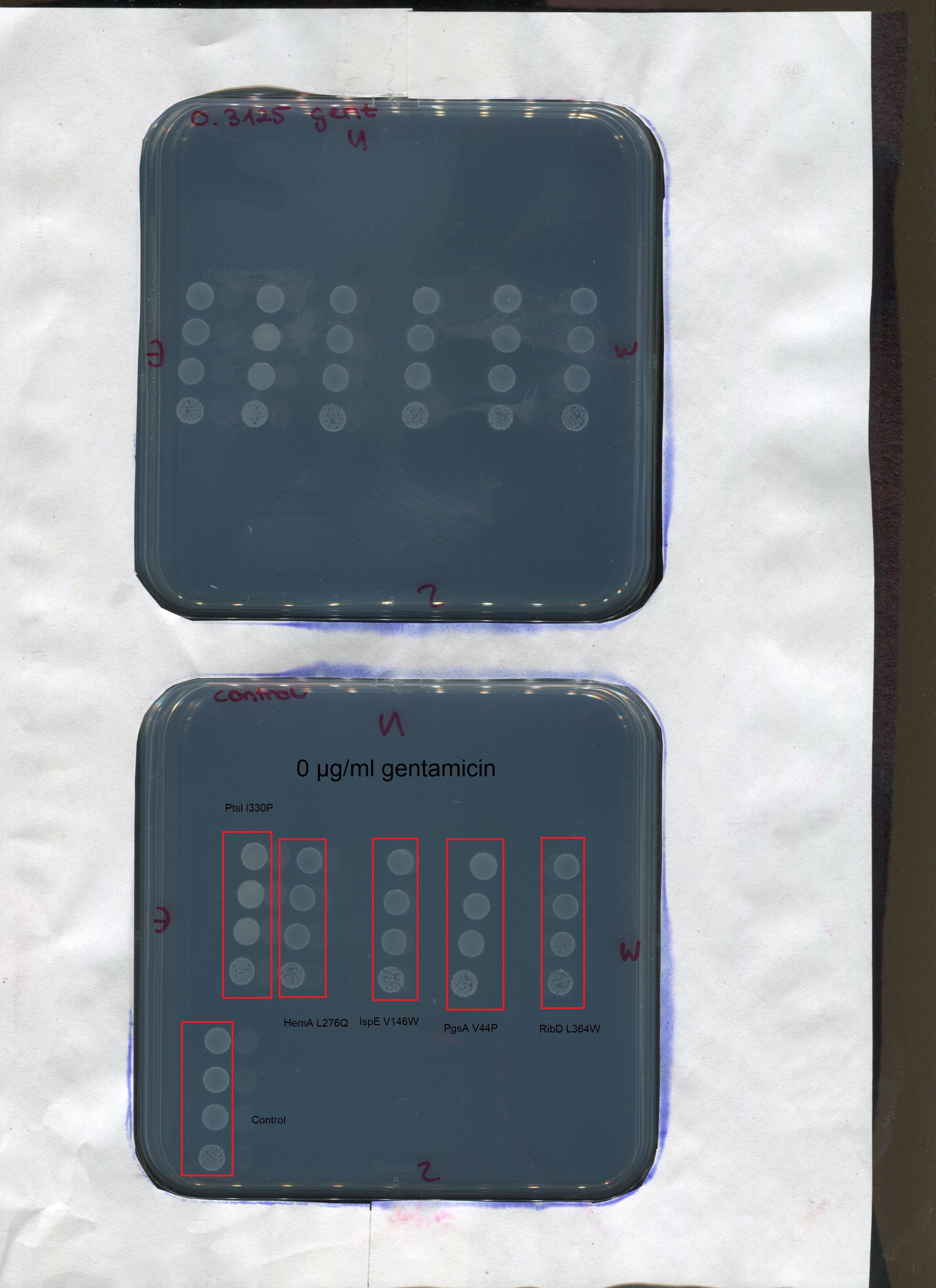

Supplement: Supplementary file 12 — Source data Fig. 2 [file 44320_2024_84_MOESM12_ESM.zip › SD_fig2/2D/0.3 and 0.tif]

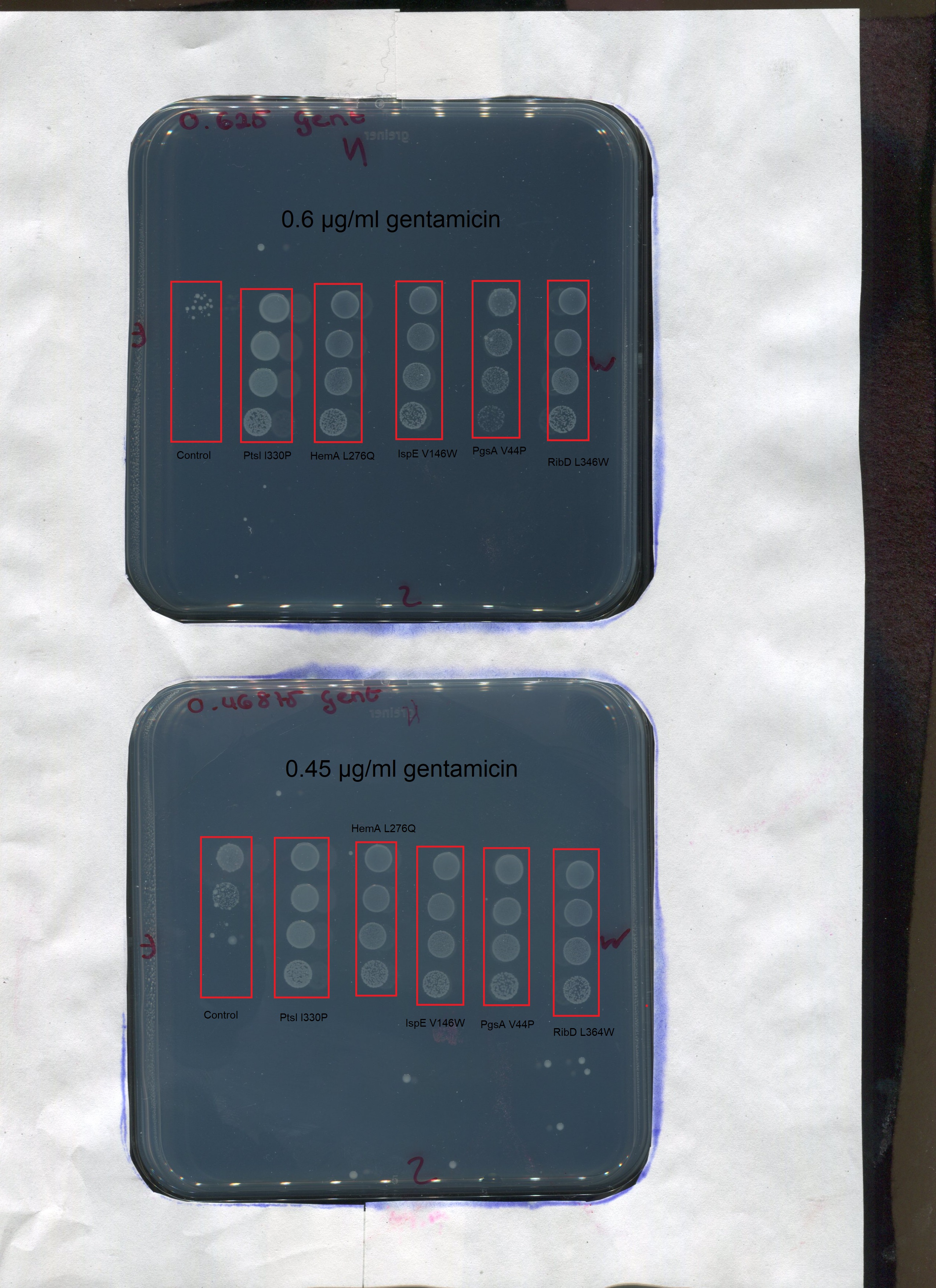

Supplement: Supplementary file 12 — Source data Fig. 2 [file 44320_2024_84_MOESM12_ESM.zip › SD_fig2/2D/0.45 and 0.6.jpg]

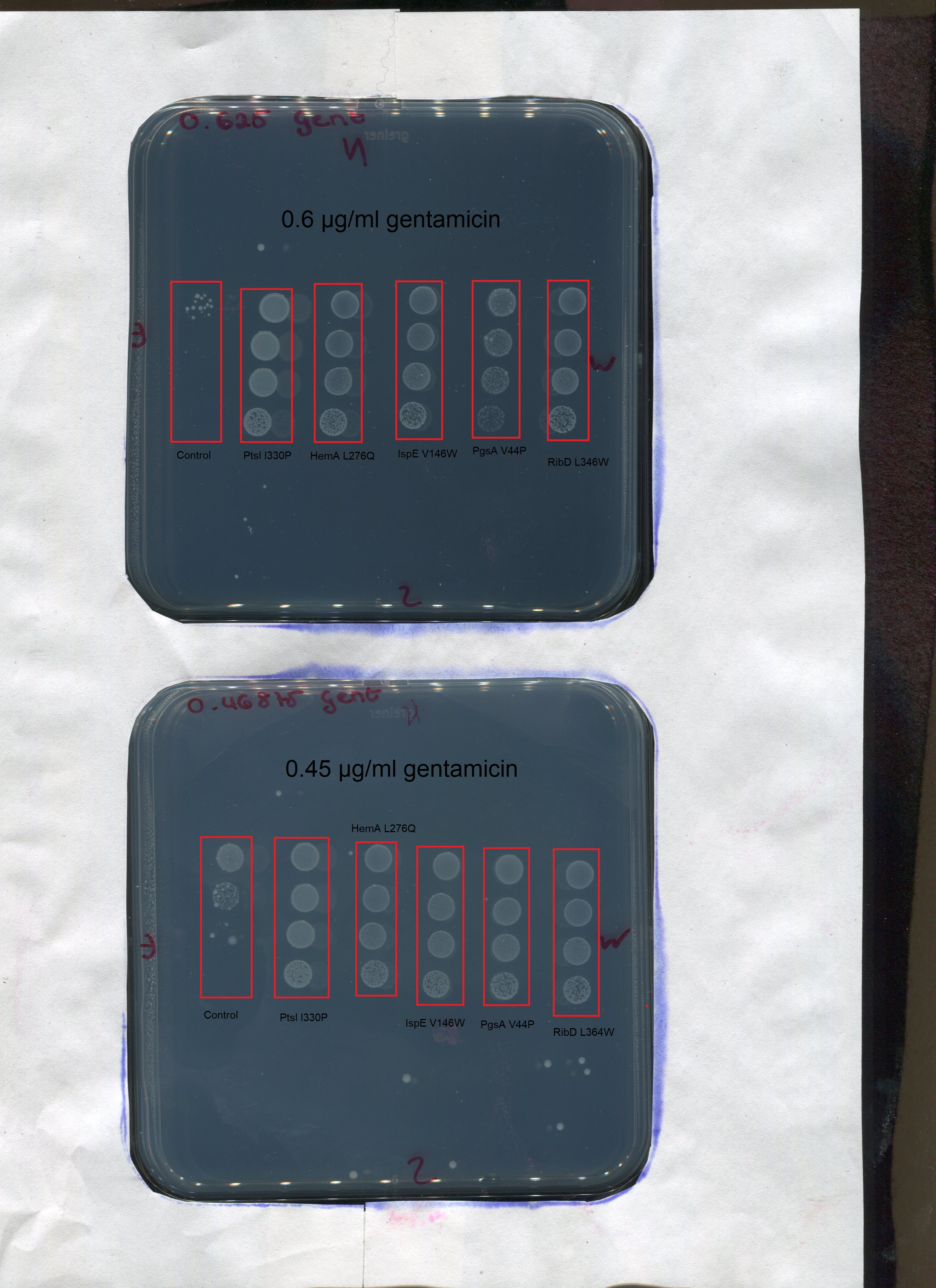

Supplement: Supplementary file 12 — Source data Fig. 2 [file 44320_2024_84_MOESM12_ESM.zip › SD_fig2/2D/0.45 and 0.6.tif]

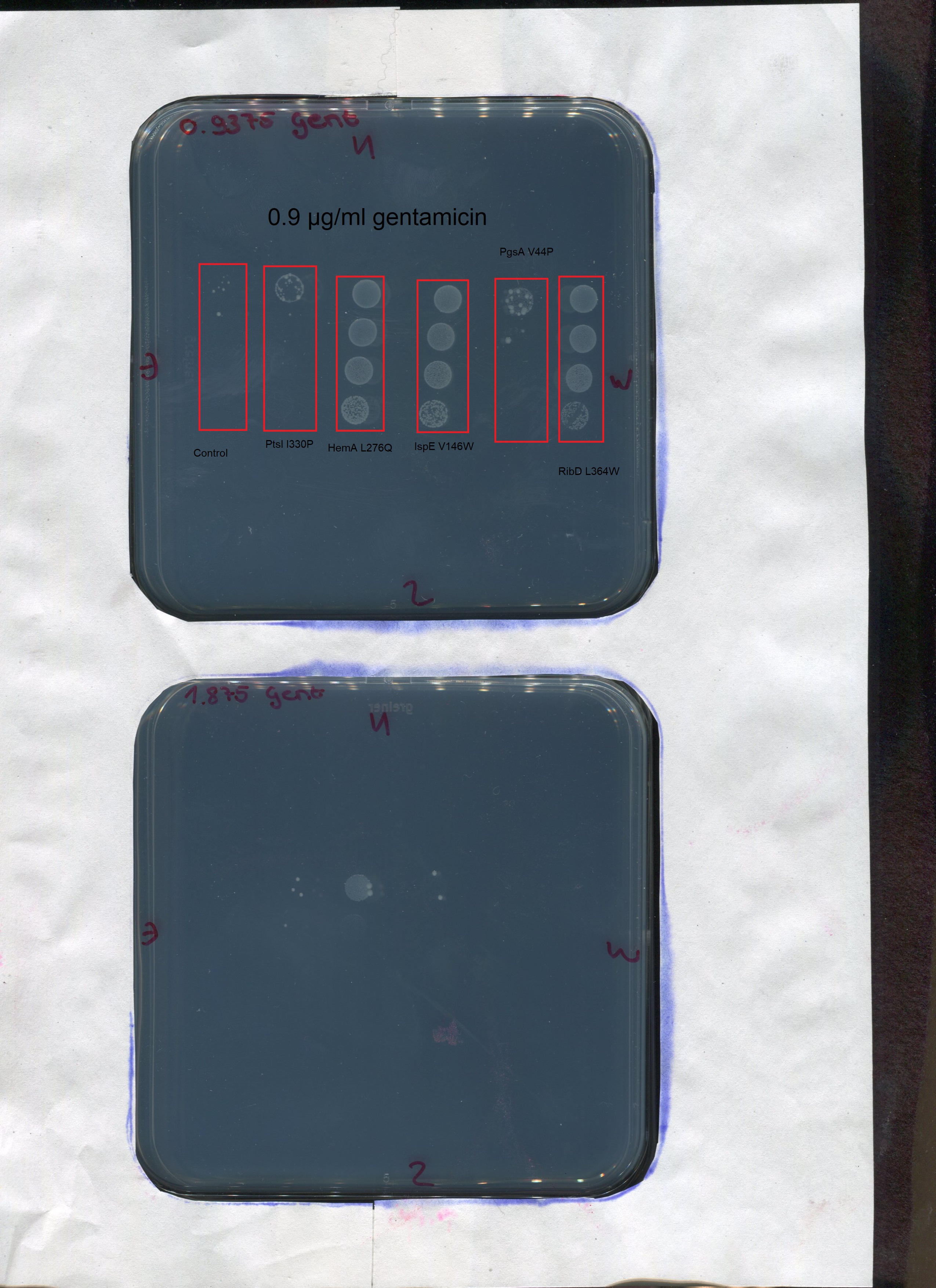

Supplement: Supplementary file 12 — Source data Fig. 2 [file 44320_2024_84_MOESM12_ESM.zip › SD_fig2/2D/0.9 and 1.8.jpg]

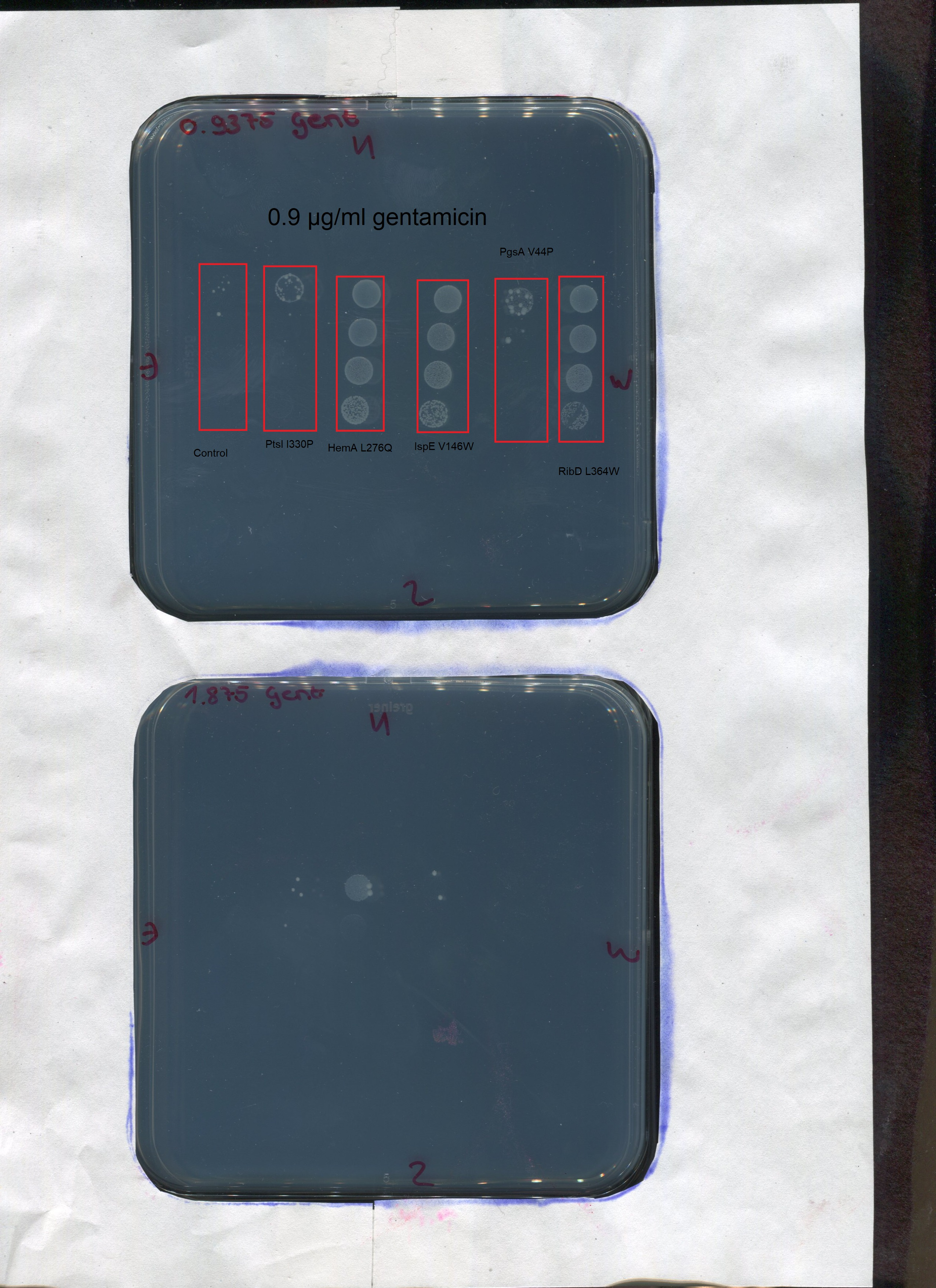

Supplement: Supplementary file 12 — Source data Fig. 2 [file 44320_2024_84_MOESM12_ESM.zip › SD_fig2/2D/0.9 and 1.8.tif]

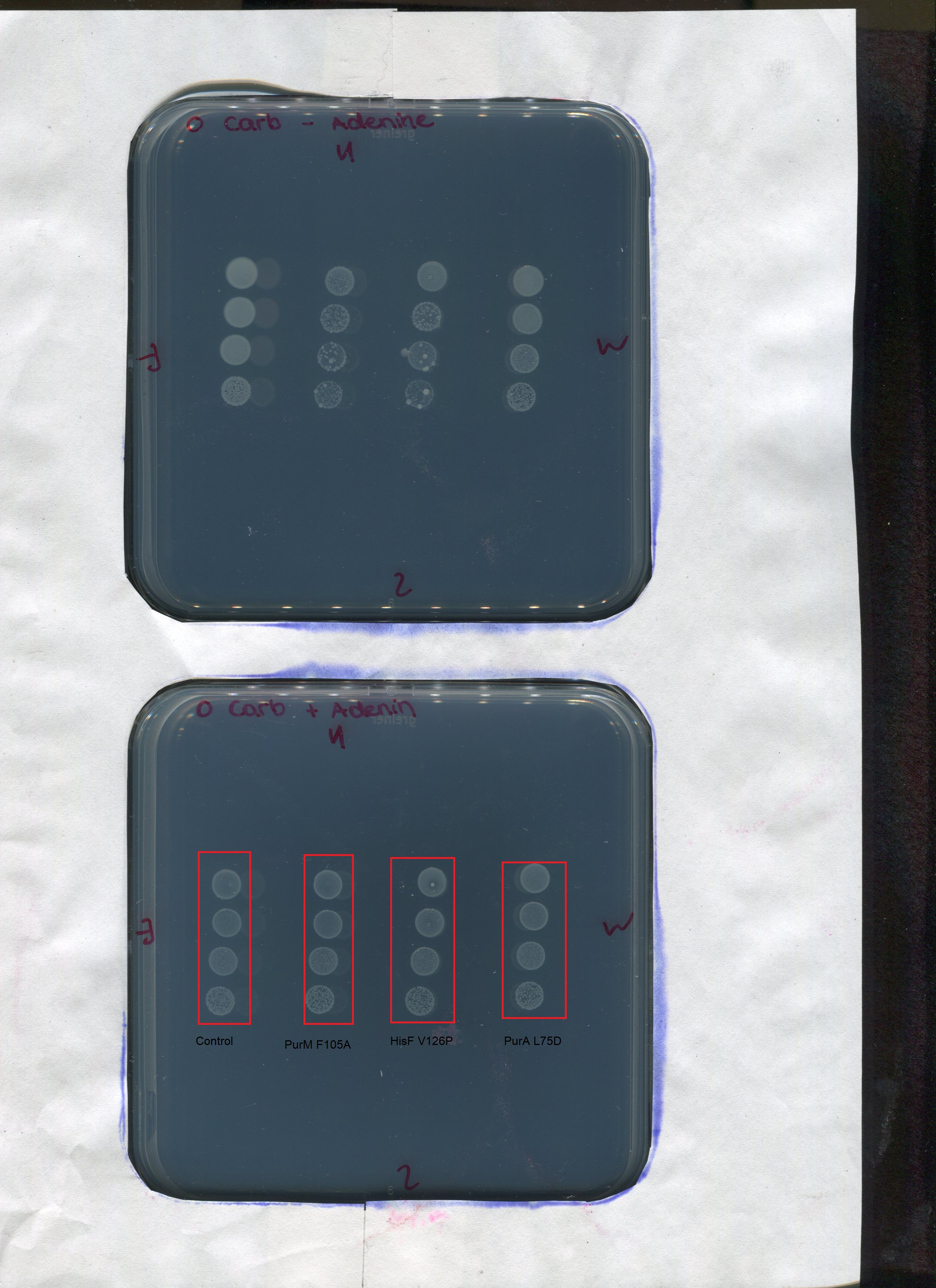

Supplement: Supplementary file 15 — Figure EV2 Source Data [file 44320_2024_84_MOESM15_ESM.zip › SD_figEV2/0.jpg]

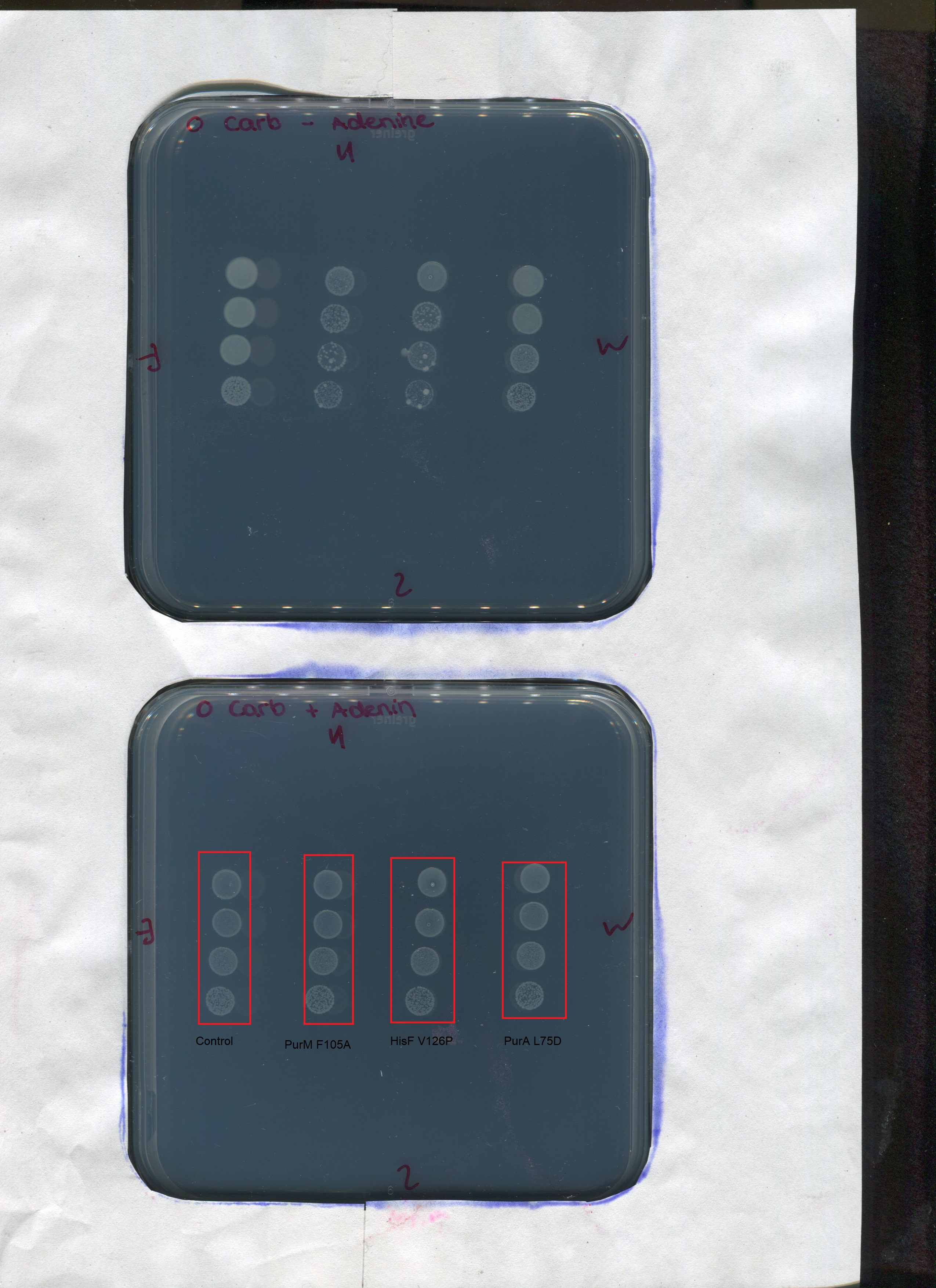

Supplement: Supplementary file 15 — Figure EV2 Source Data [file 44320_2024_84_MOESM15_ESM.zip › SD_figEV2/0.tif]

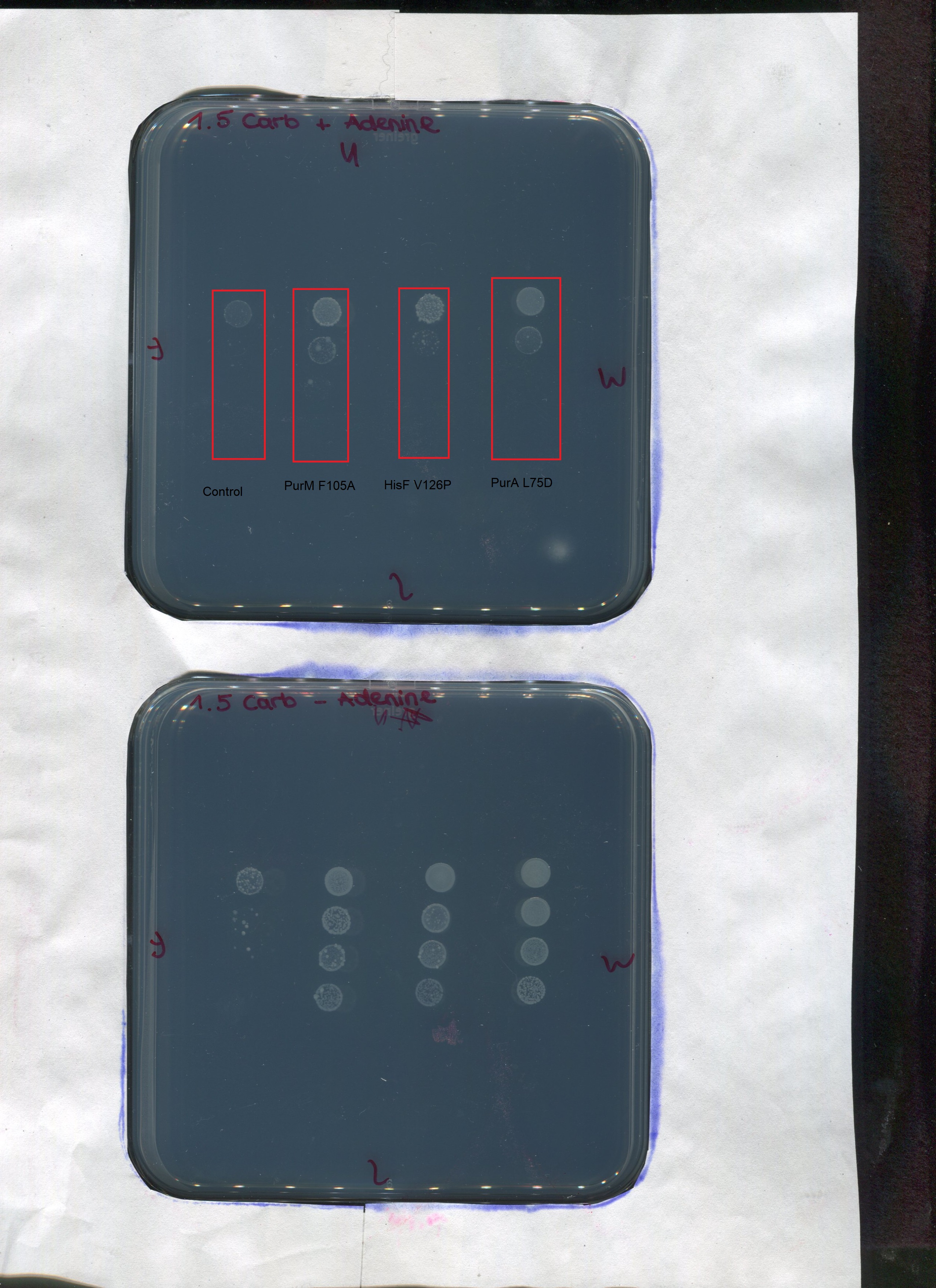

Supplement: Supplementary file 15 — Figure EV2 Source Data [file 44320_2024_84_MOESM15_ESM.zip › SD_figEV2/1.5.jpg]

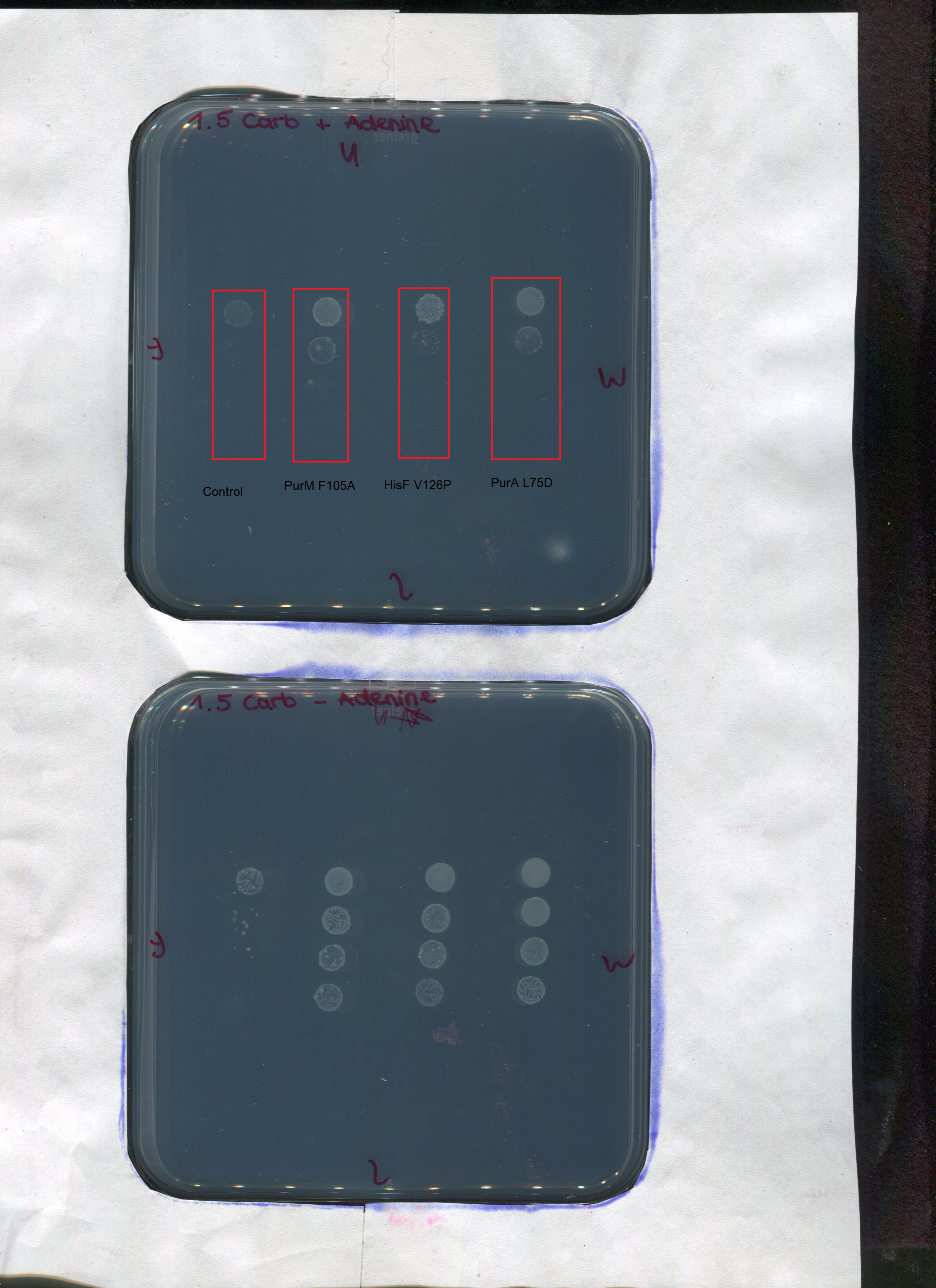

Supplement: Supplementary file 15 — Figure EV2 Source Data [file 44320_2024_84_MOESM15_ESM.zip › SD_figEV2/1.5.tif]

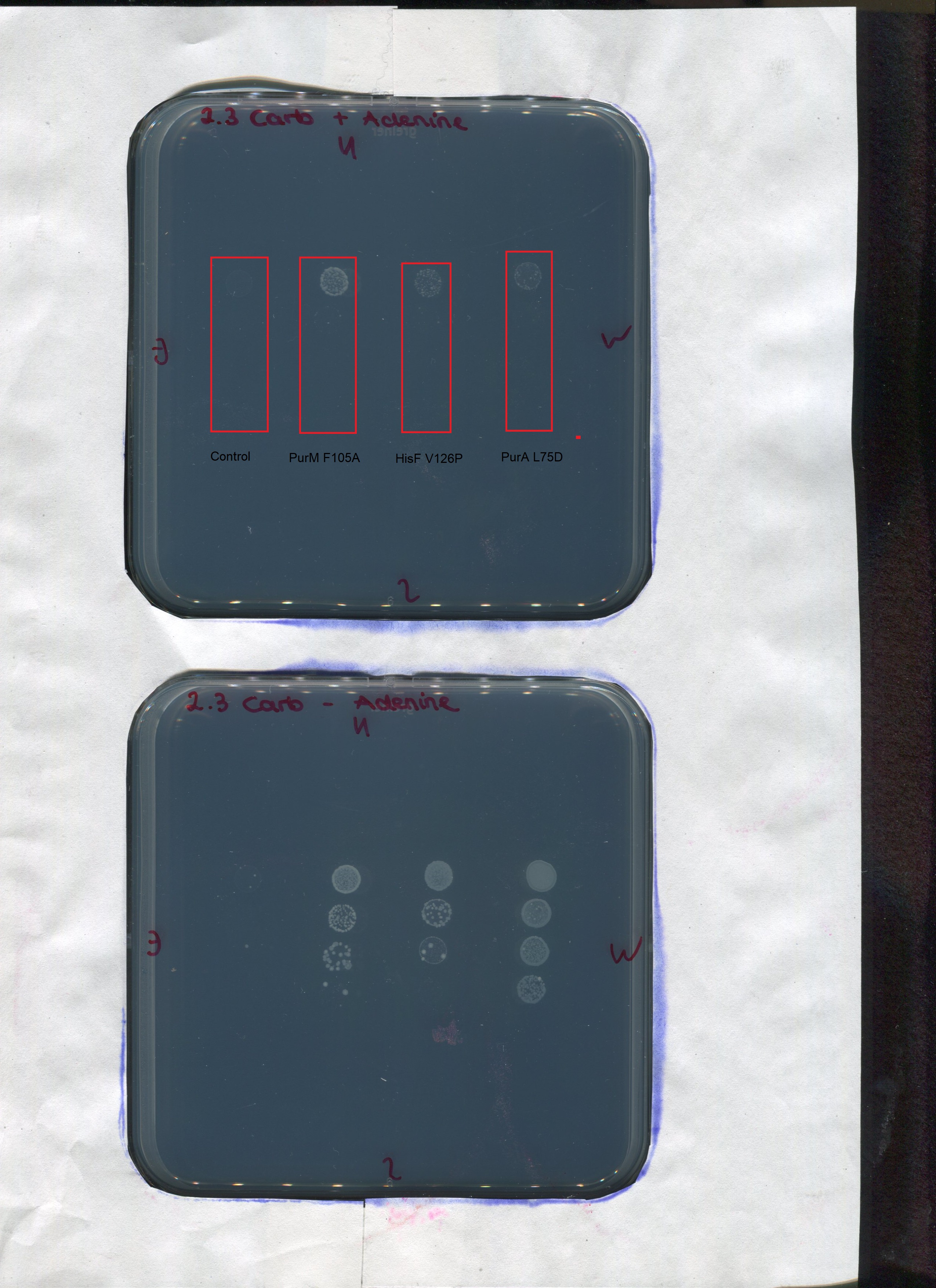

Supplement: Supplementary file 15 — Figure EV2 Source Data [file 44320_2024_84_MOESM15_ESM.zip › SD_figEV2/2.3.jpg]

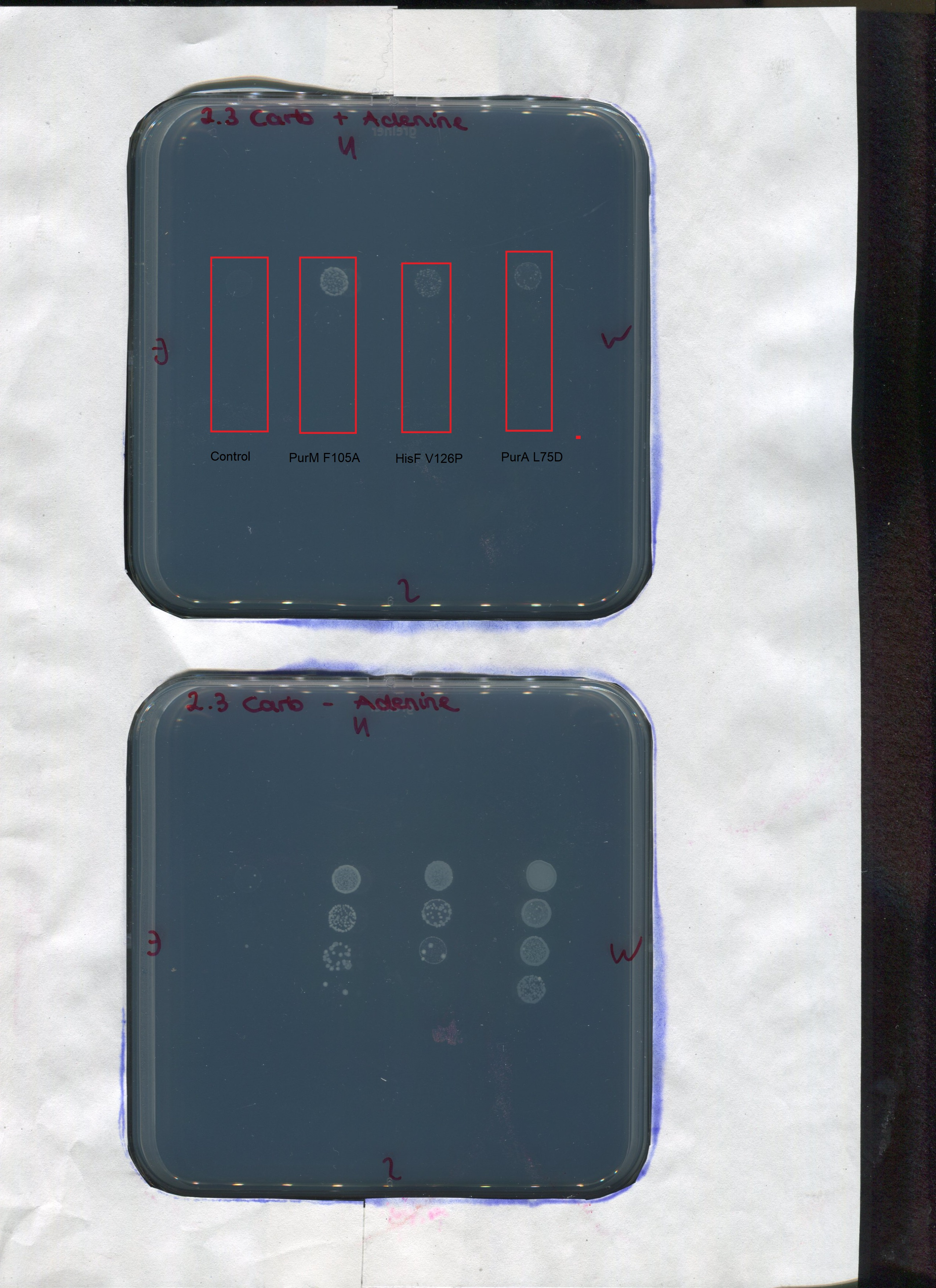

Supplement: Supplementary file 15 — Figure EV2 Source Data [file 44320_2024_84_MOESM15_ESM.zip › SD_figEV2/2.3.tif]

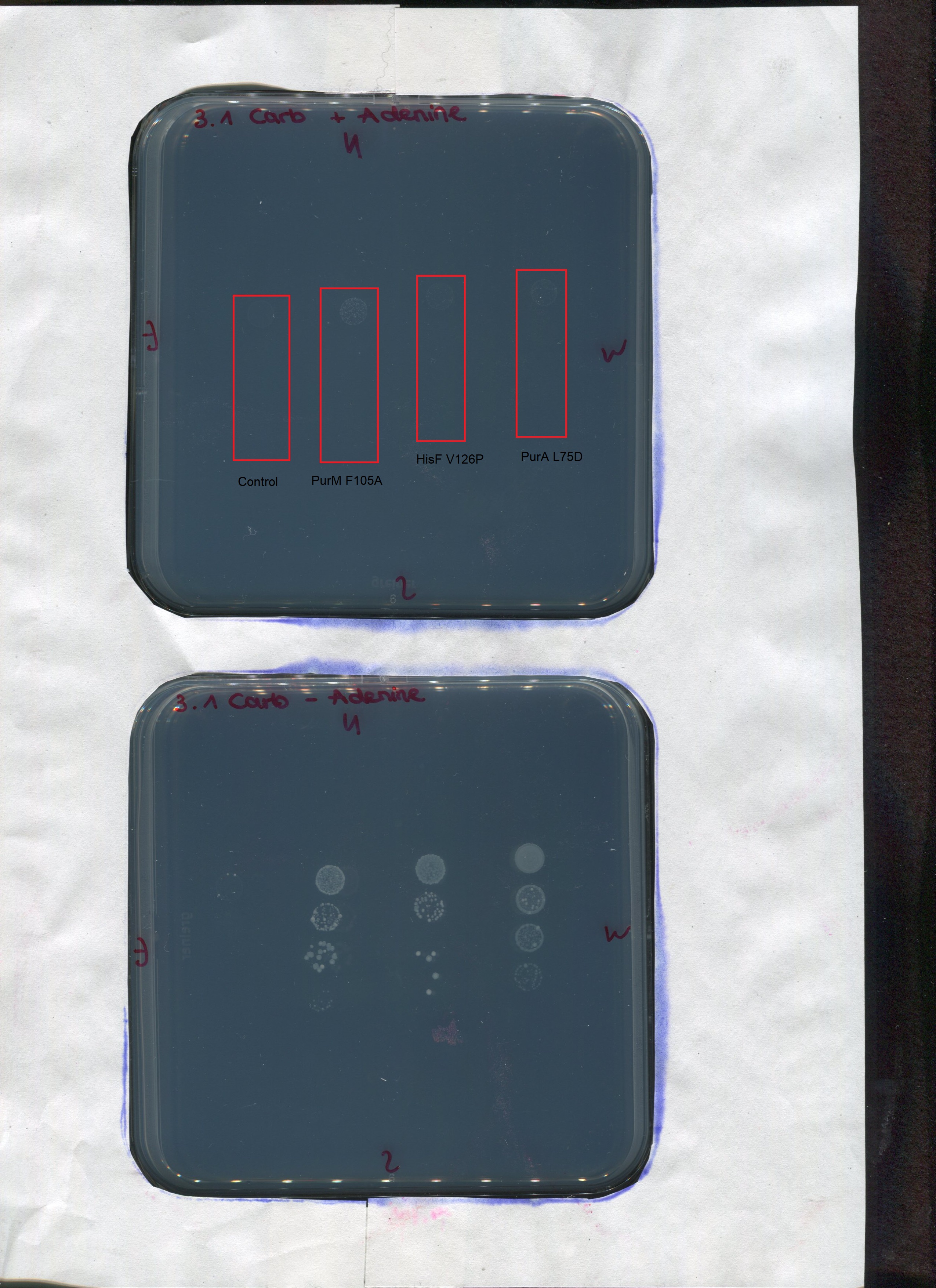

Supplement: Supplementary file 15 — Figure EV2 Source Data [file 44320_2024_84_MOESM15_ESM.zip › SD_figEV2/3.1.jpg]

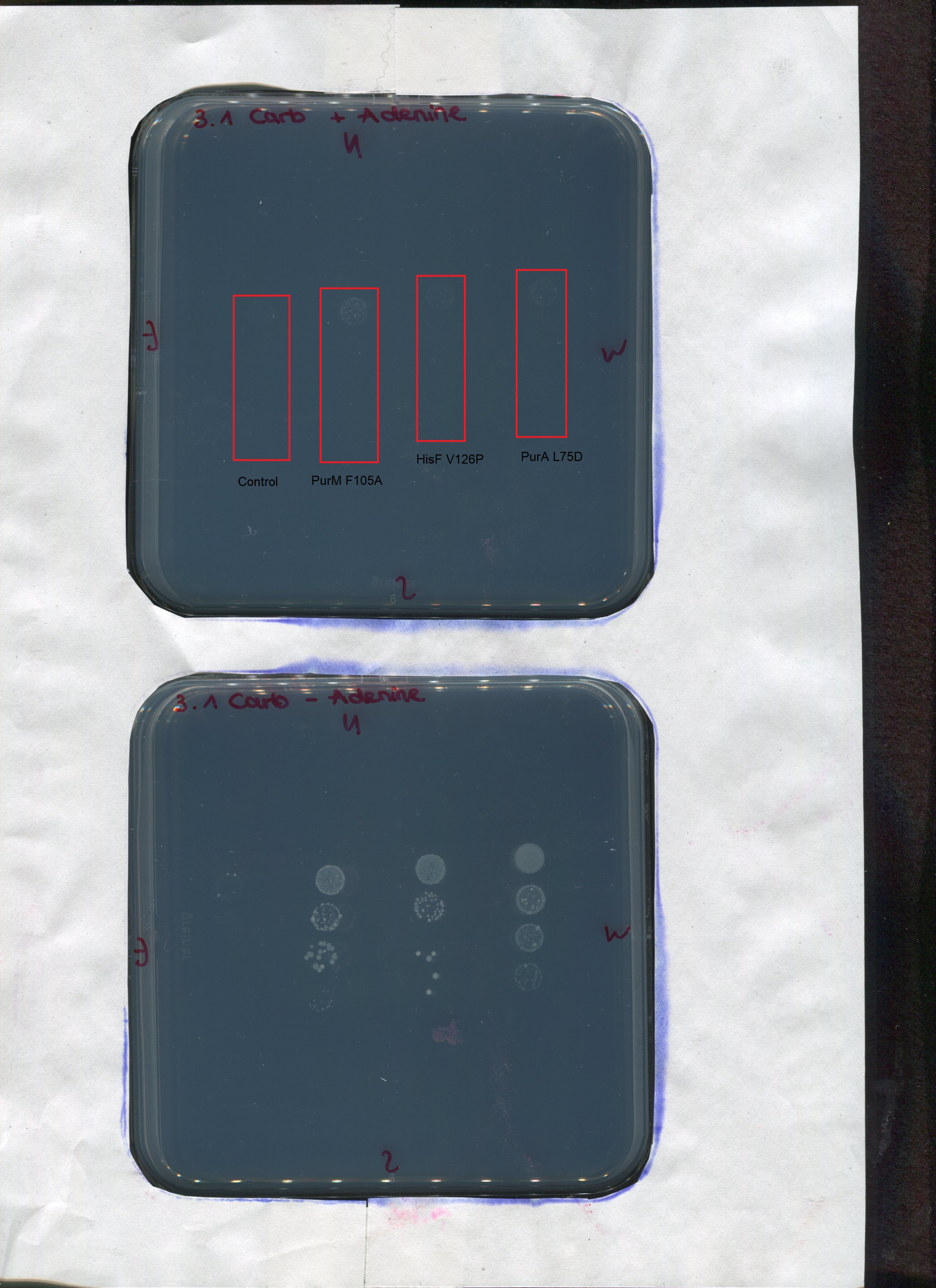

Supplement: Supplementary file 15 — Figure EV2 Source Data [file 44320_2024_84_MOESM15_ESM.zip › SD_figEV2/3.1.tif]

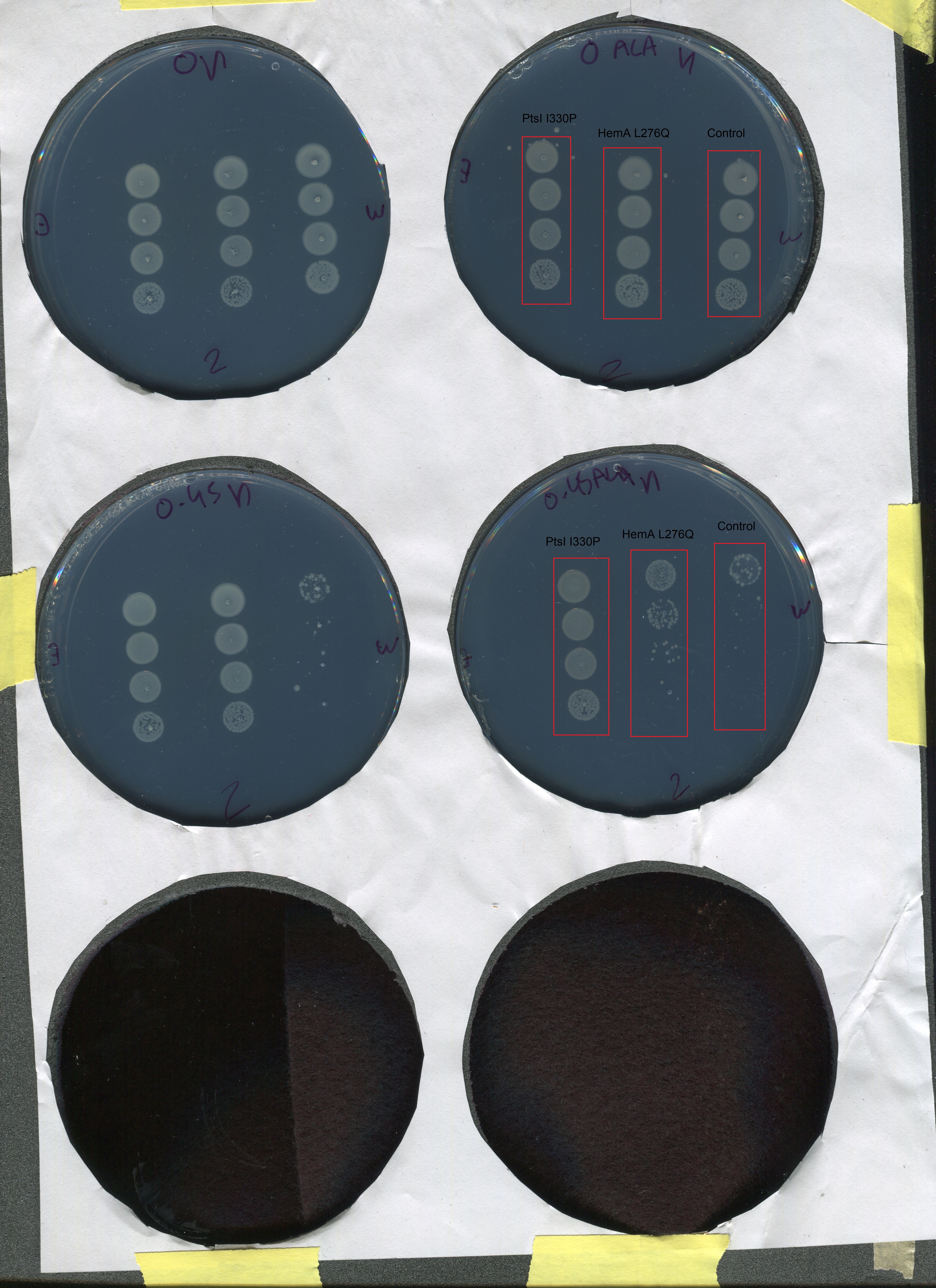

Supplement: Supplementary file 16 — Figure EV3 Source Data [file 44320_2024_84_MOESM16_ESM.zip › SD_figEV3/ALA 0 and 0.45.jpg]

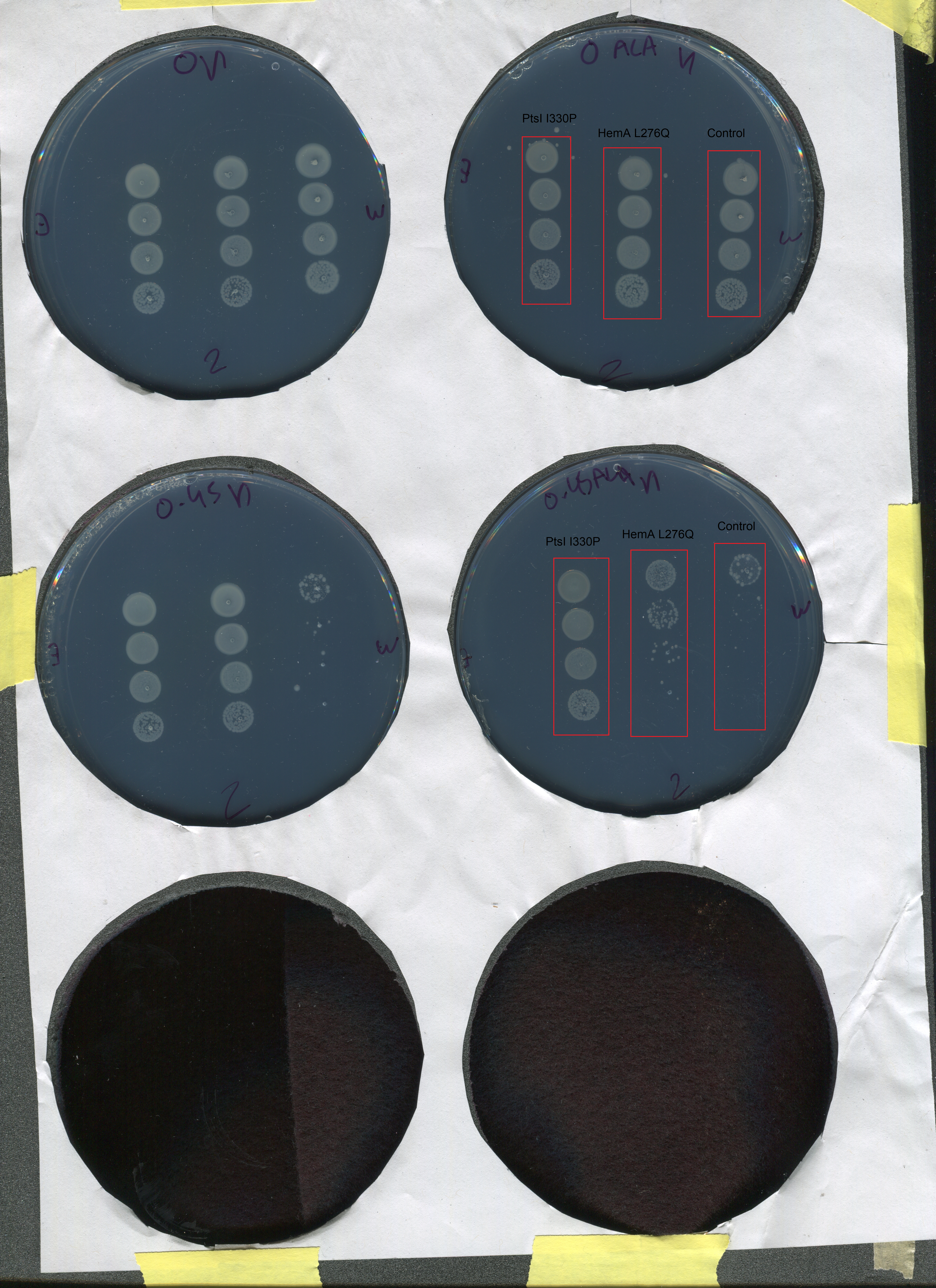

Supplement: Supplementary file 16 — Figure EV3 Source Data [file 44320_2024_84_MOESM16_ESM.zip › SD_figEV3/ALA 0 and 0.45.tif]

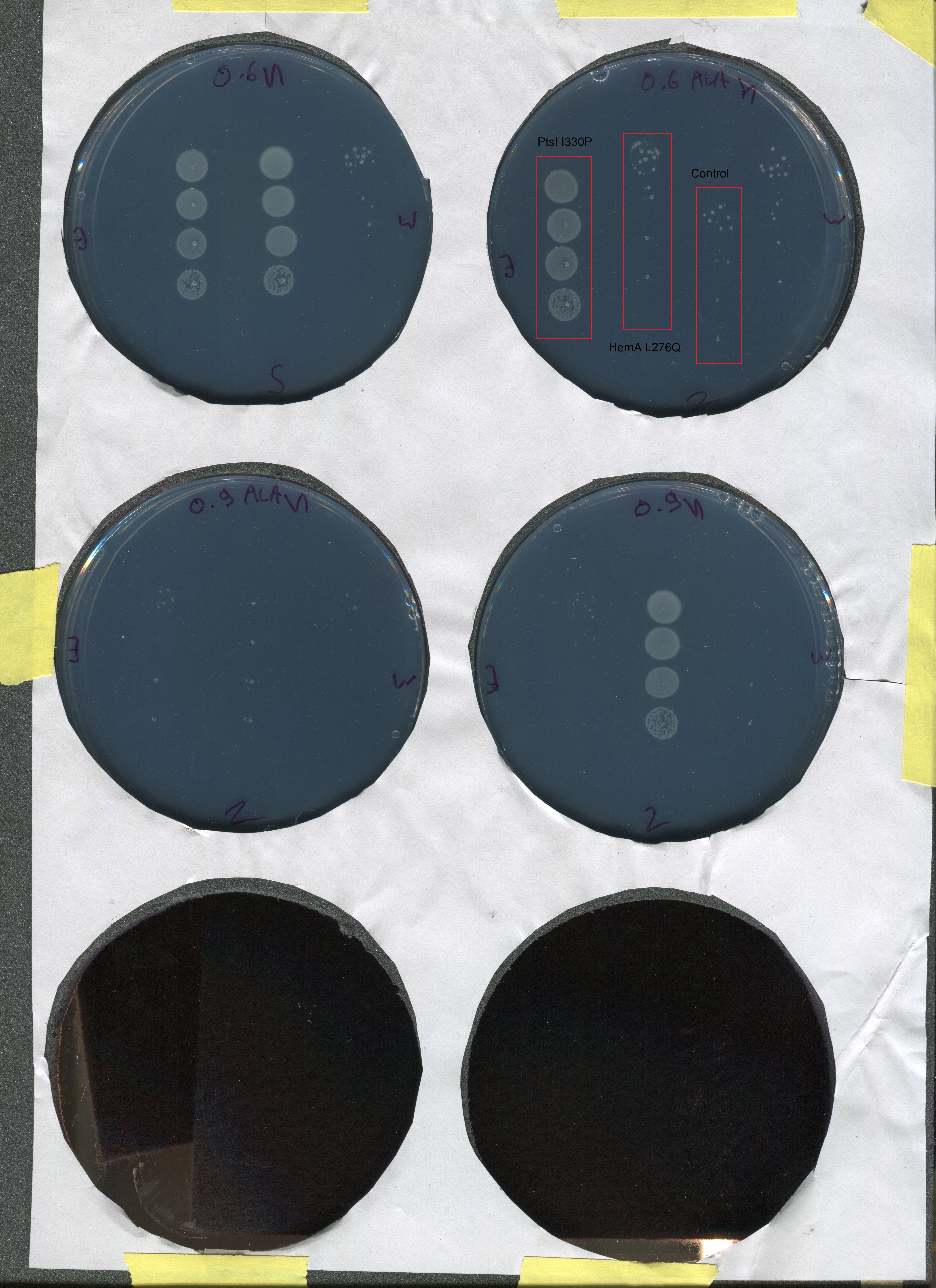

Supplement: Supplementary file 16 — Figure EV3 Source Data [file 44320_2024_84_MOESM16_ESM.zip › SD_figEV3/ALA 0.6 and 0.9.jpg]

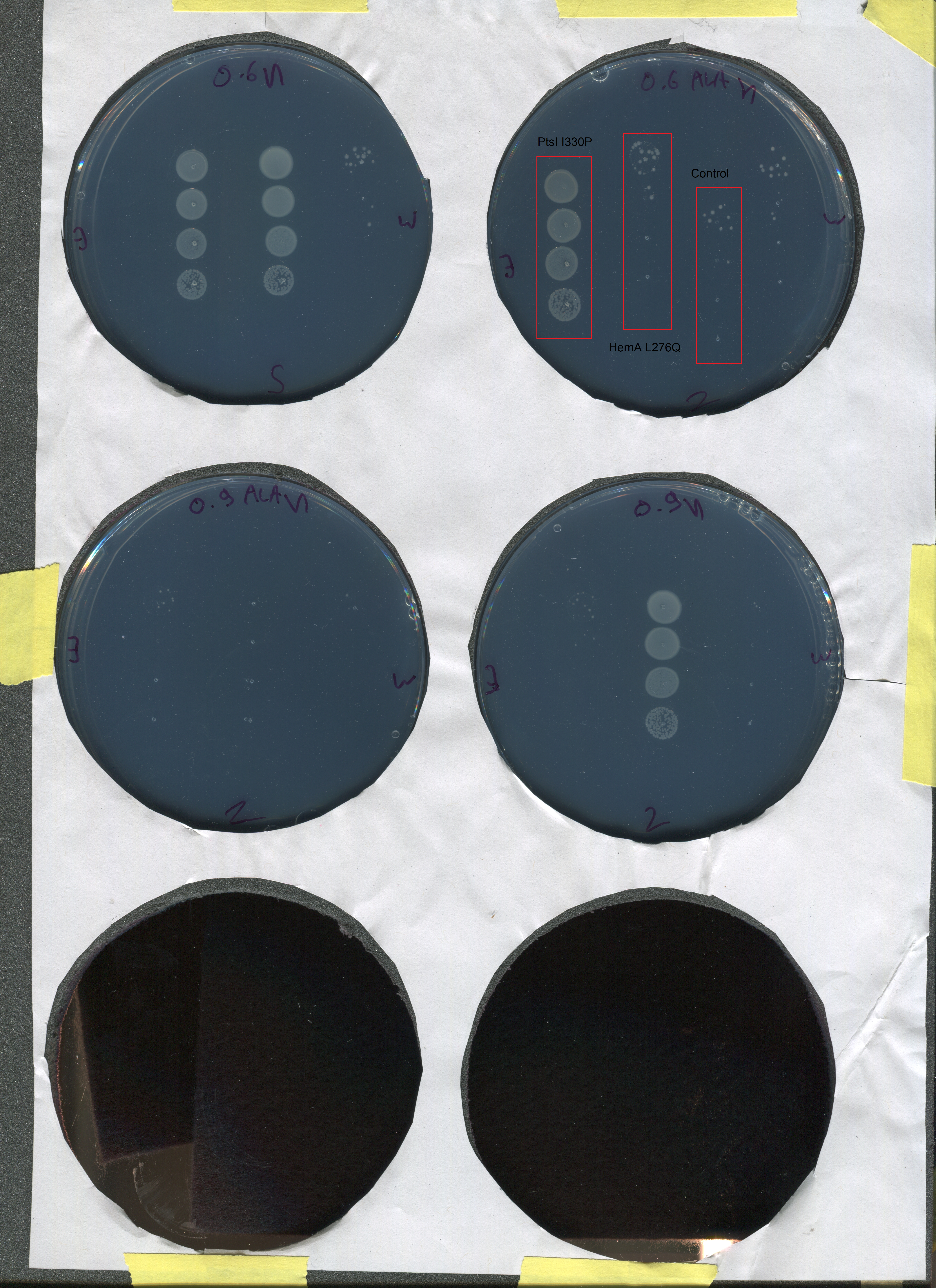

Supplement: Supplementary file 16 — Figure EV3 Source Data [file 44320_2024_84_MOESM16_ESM.zip › SD_figEV3/ALA 0.6 and 0.9.tif]

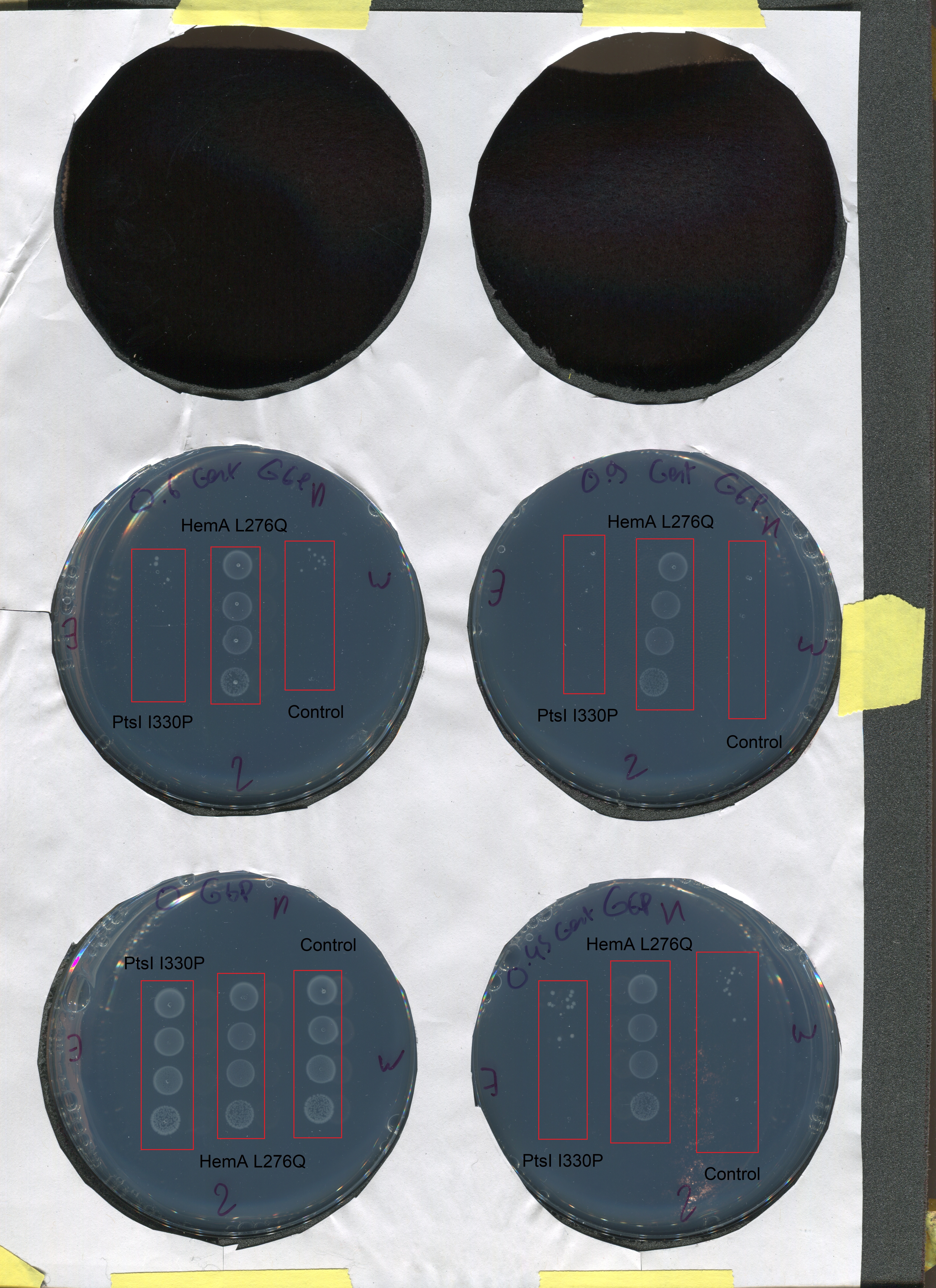

Supplement: Supplementary file 16 — Figure EV3 Source Data [file 44320_2024_84_MOESM16_ESM.zip › SD_figEV3/G6P.jpg]

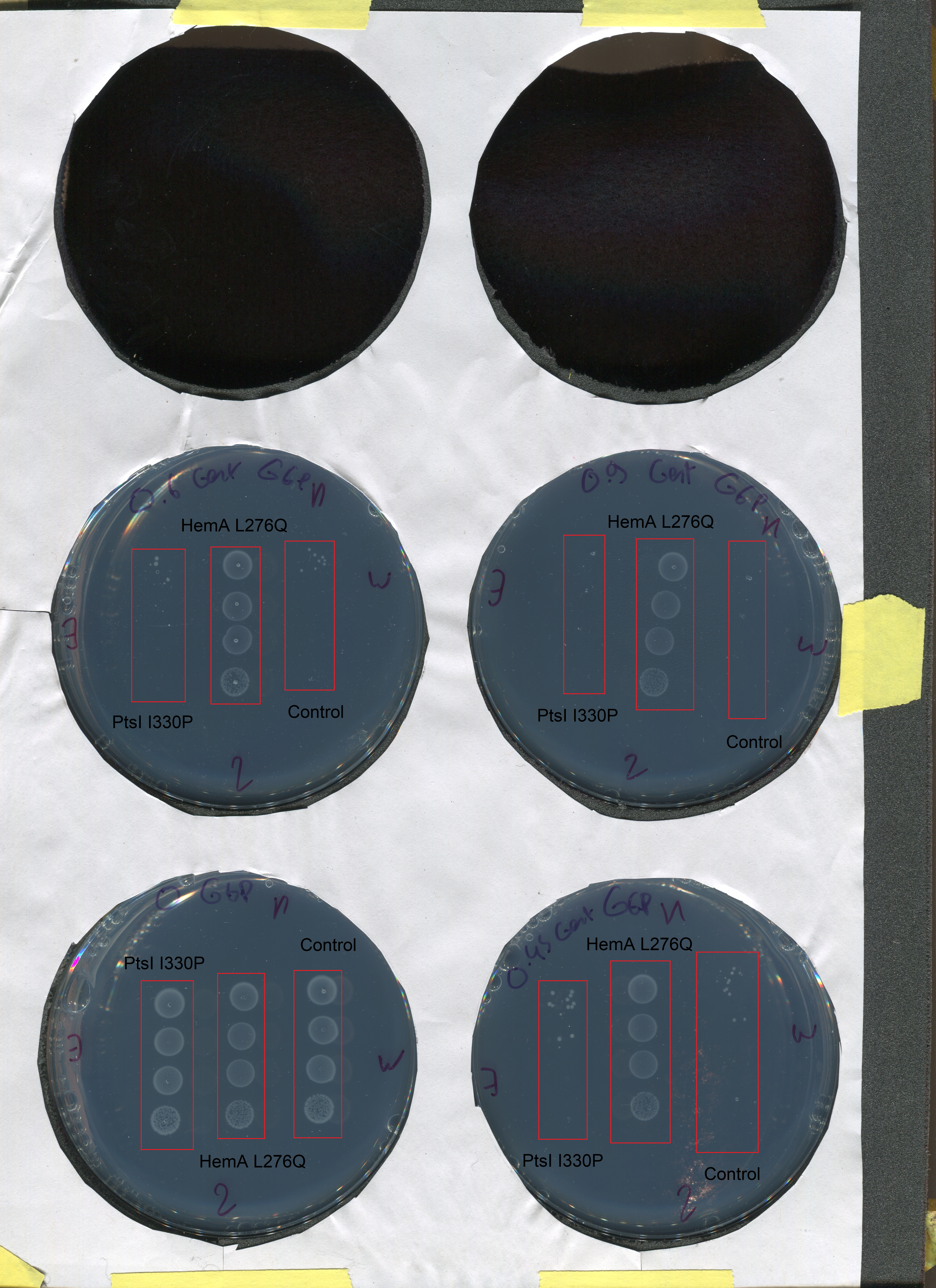

Supplement: Supplementary file 16 — Figure EV3 Source Data [file 44320_2024_84_MOESM16_ESM.zip › SD_figEV3/G6P.tif]

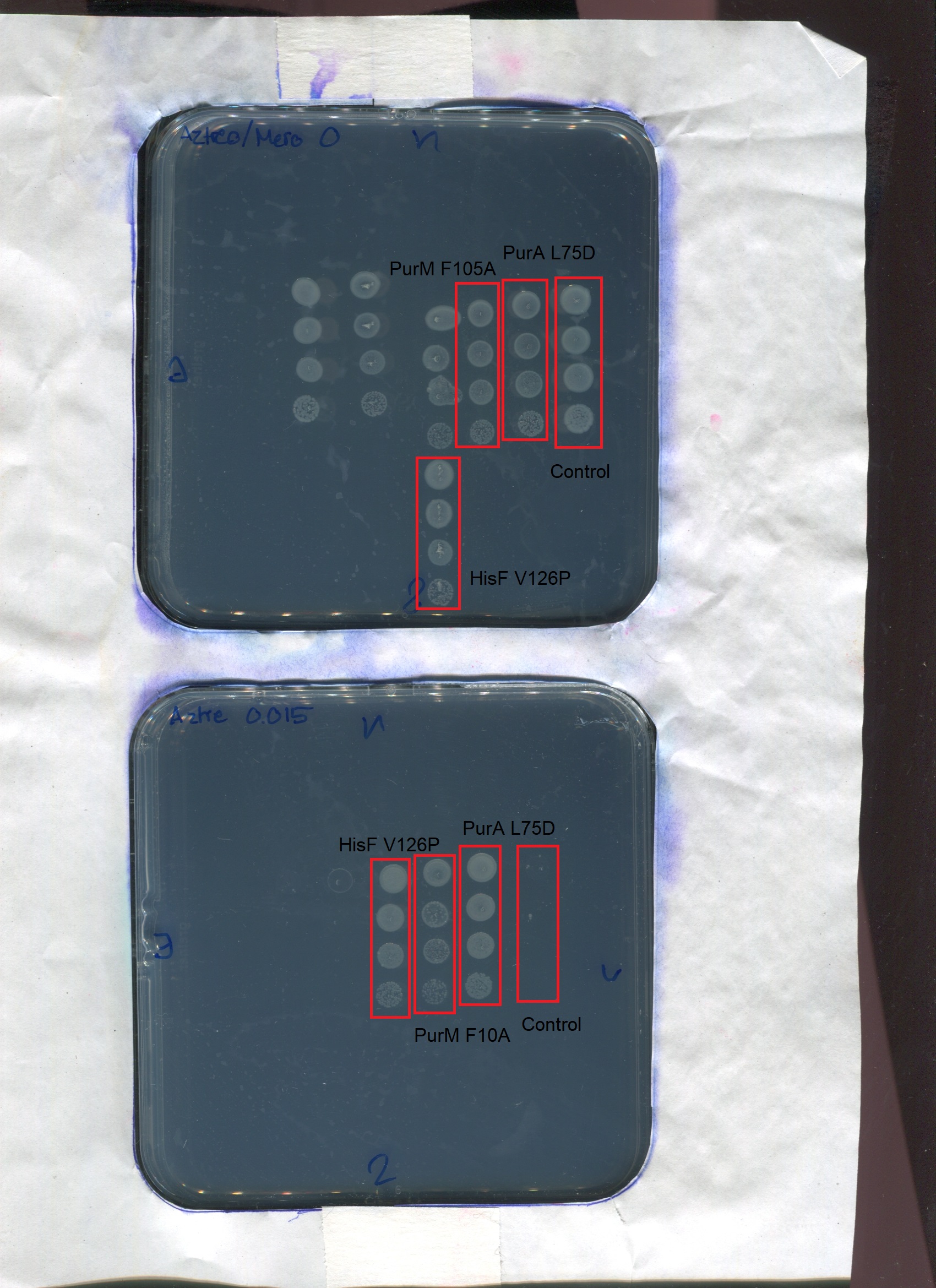

Supplement: Supplementary file 17 — Figure S3 Source Data [file 44320_2024_84_MOESM17_ESM.zip › SD_figS3/S3A/0 and 0.015.jpg]

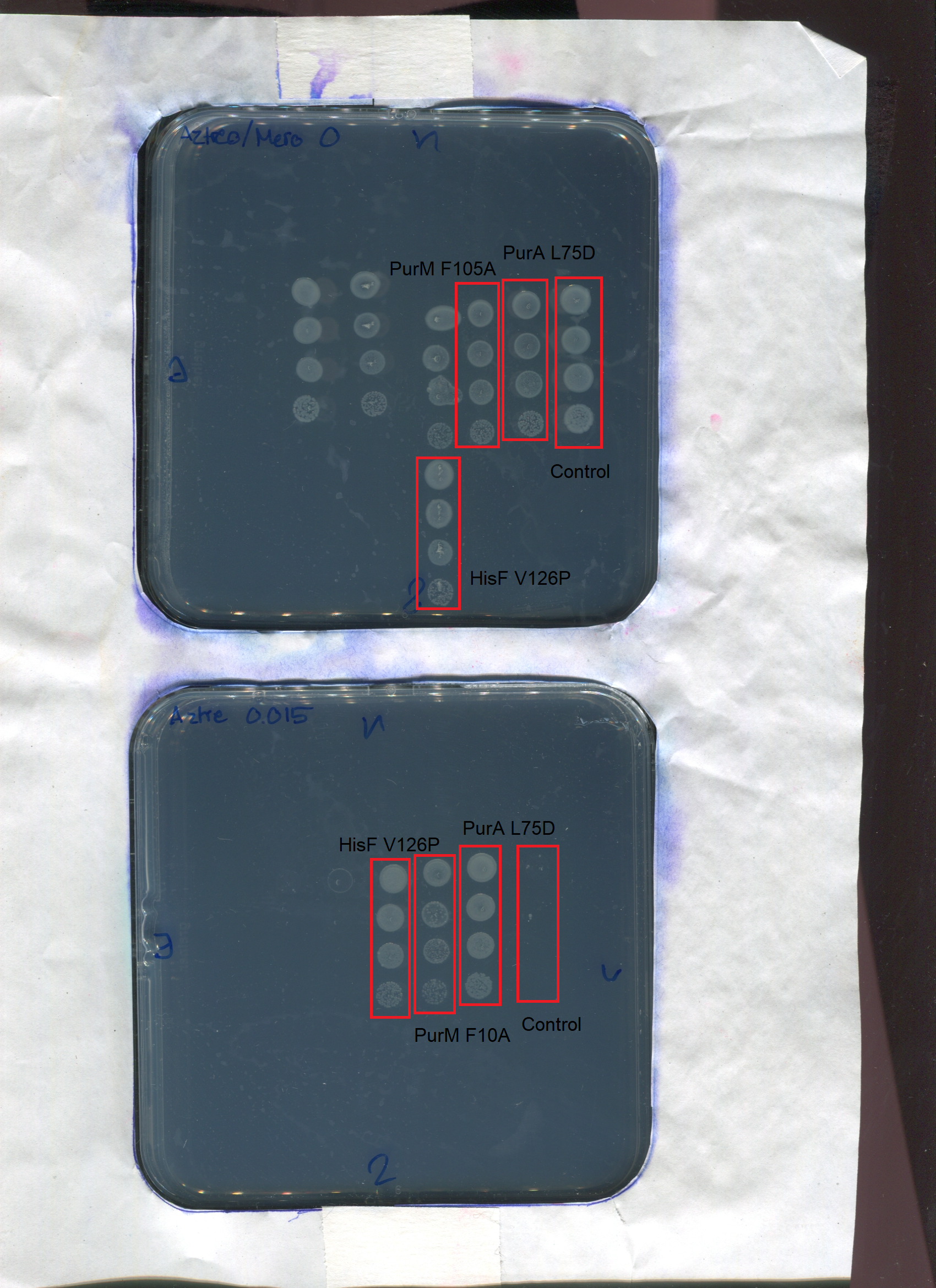

Supplement: Supplementary file 17 — Figure S3 Source Data [file 44320_2024_84_MOESM17_ESM.zip › SD_figS3/S3A/0 and 0.015.tif]

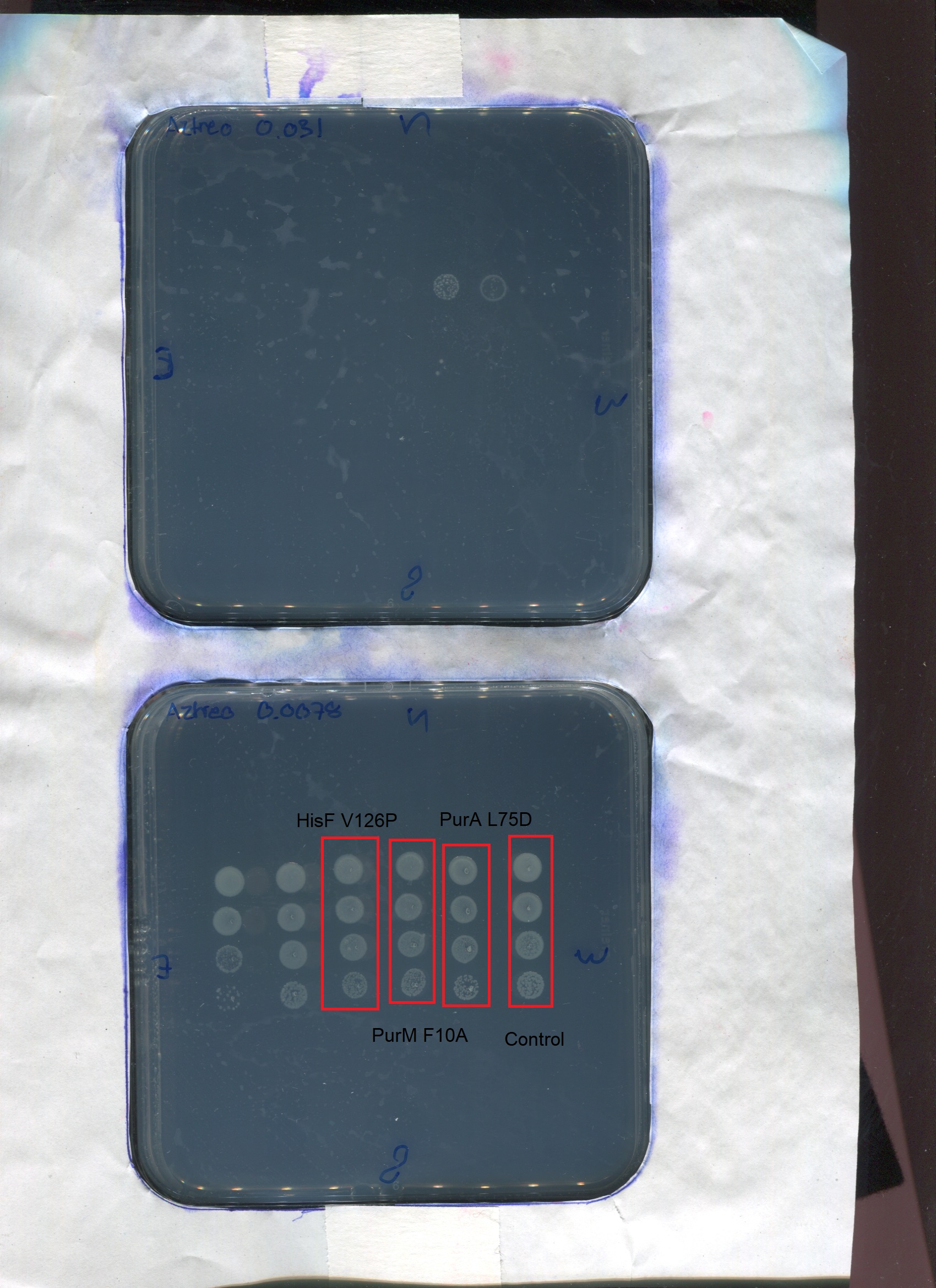

Supplement: Supplementary file 17 — Figure S3 Source Data [file 44320_2024_84_MOESM17_ESM.zip › SD_figS3/S3A/0.0078.jpg]

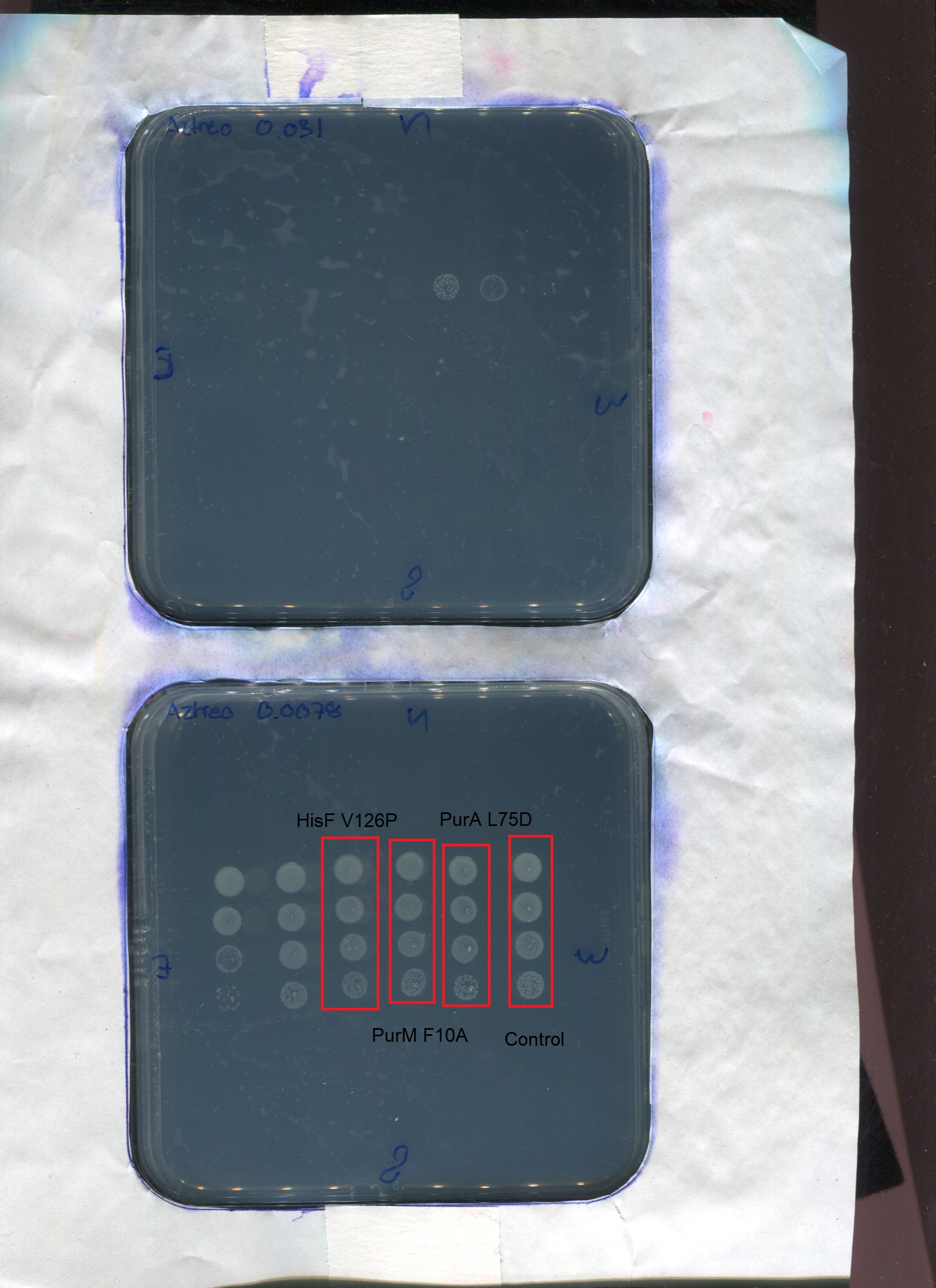

Supplement: Supplementary file 17 — Figure S3 Source Data [file 44320_2024_84_MOESM17_ESM.zip › SD_figS3/S3A/0.0078.tif]

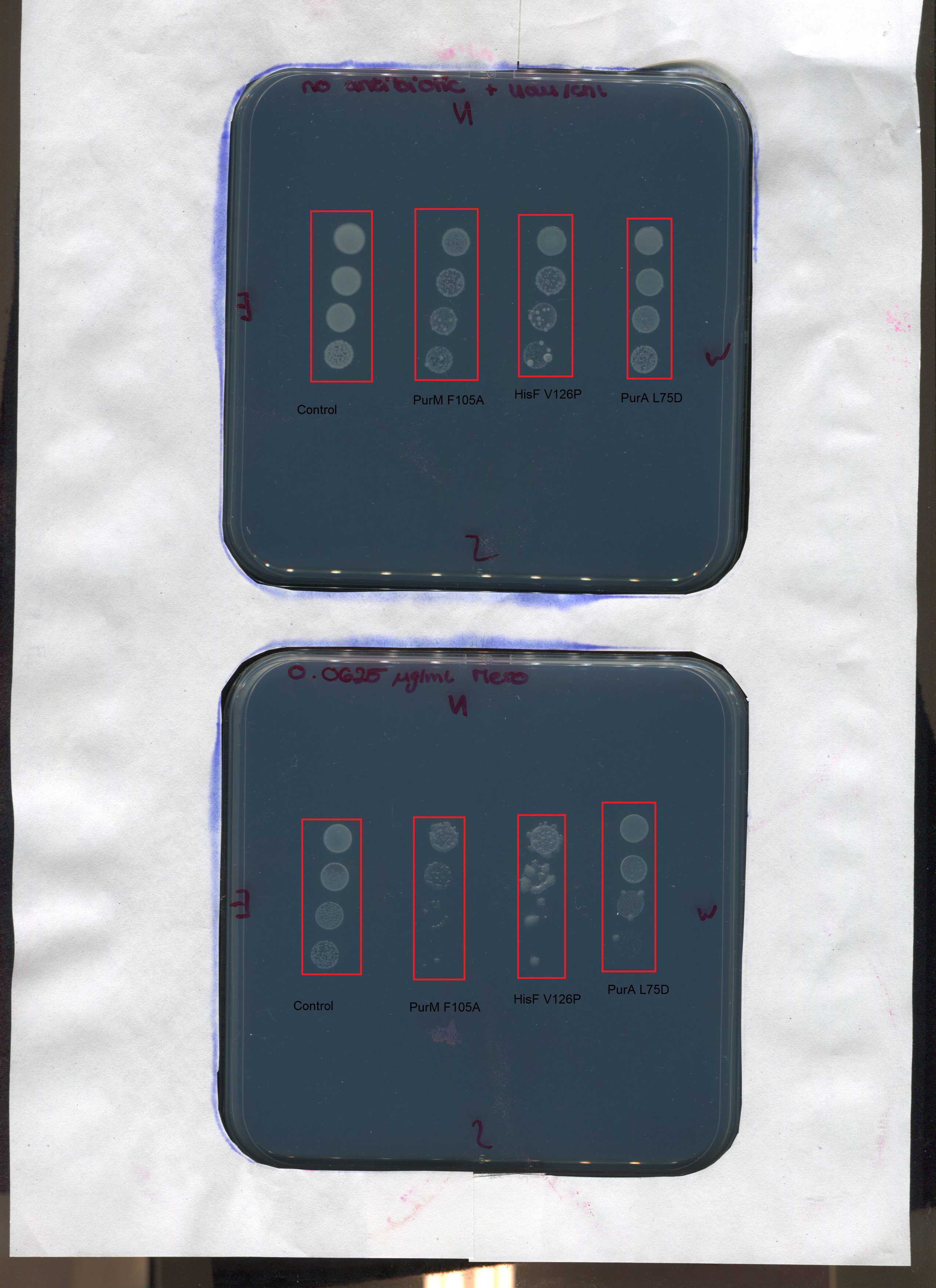

Supplement: Supplementary file 17 — Figure S3 Source Data [file 44320_2024_84_MOESM17_ESM.zip › SD_figS3/S3B/Mero + control.jpg]

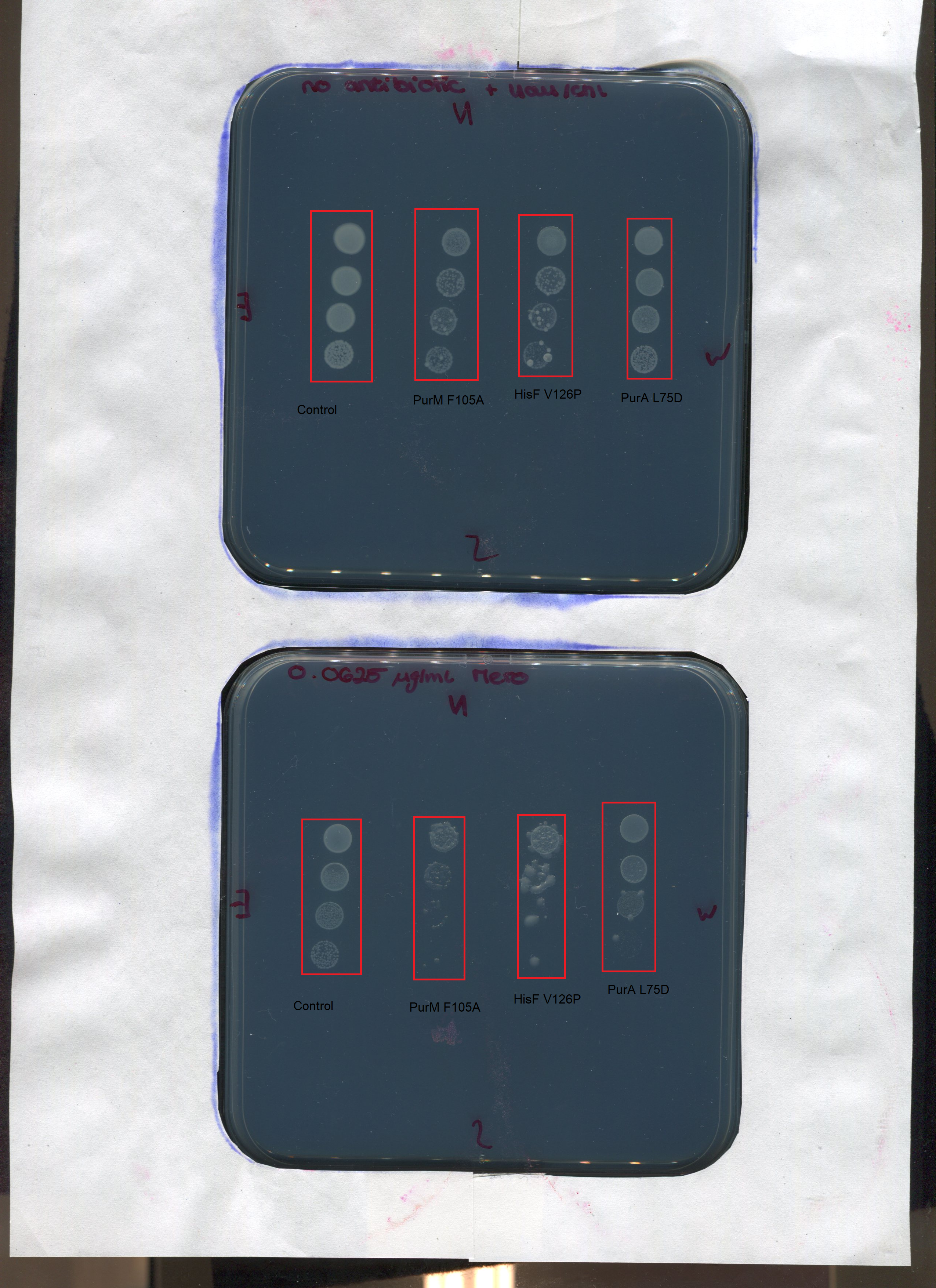

Supplement: Supplementary file 17 — Figure S3 Source Data [file 44320_2024_84_MOESM17_ESM.zip › SD_figS3/S3B/Mero + control.tif]

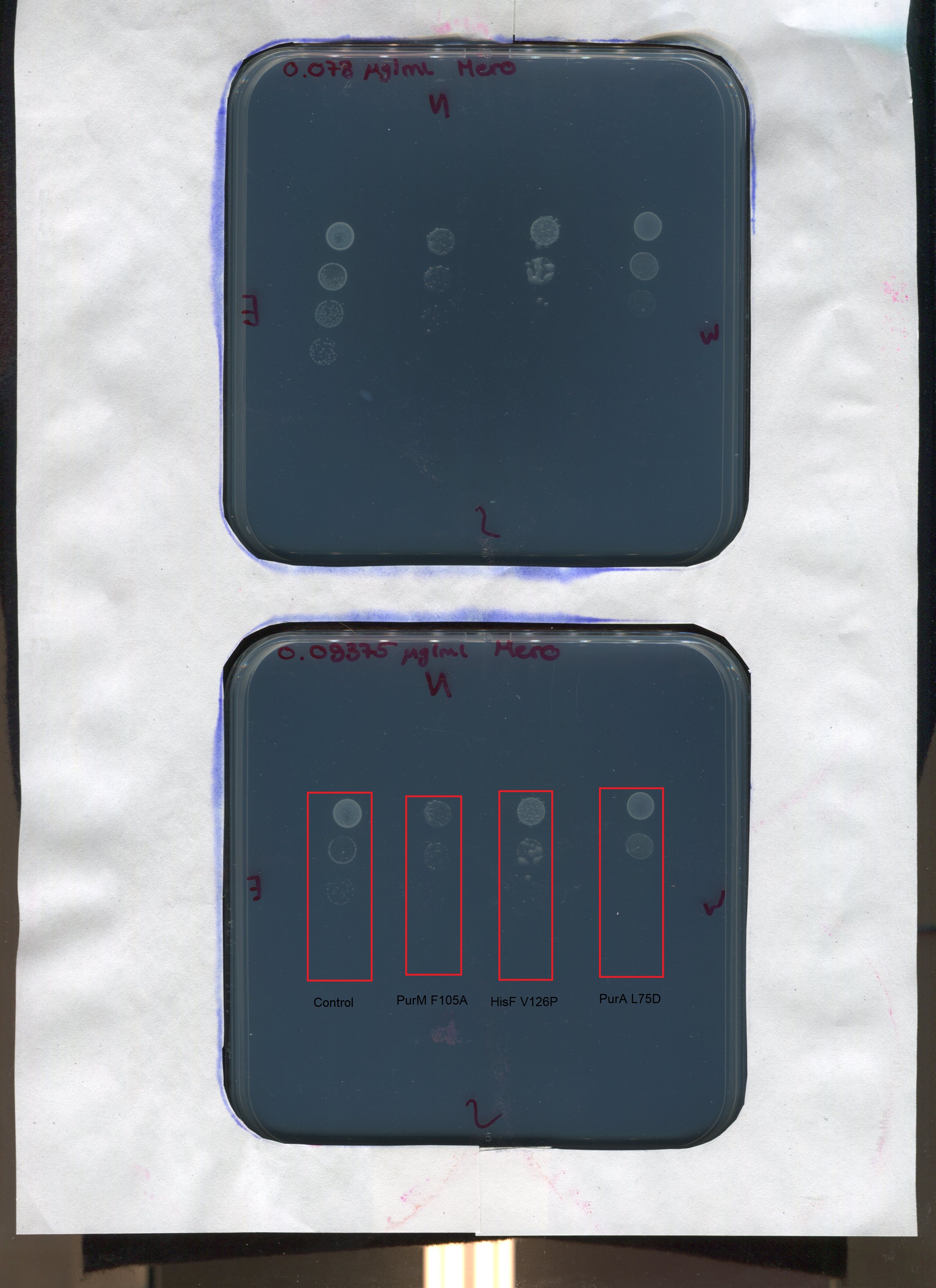

Supplement: Supplementary file 17 — Figure S3 Source Data [file 44320_2024_84_MOESM17_ESM.zip › SD_figS3/S3B/Mero 0.07 + 0.09.jpg]

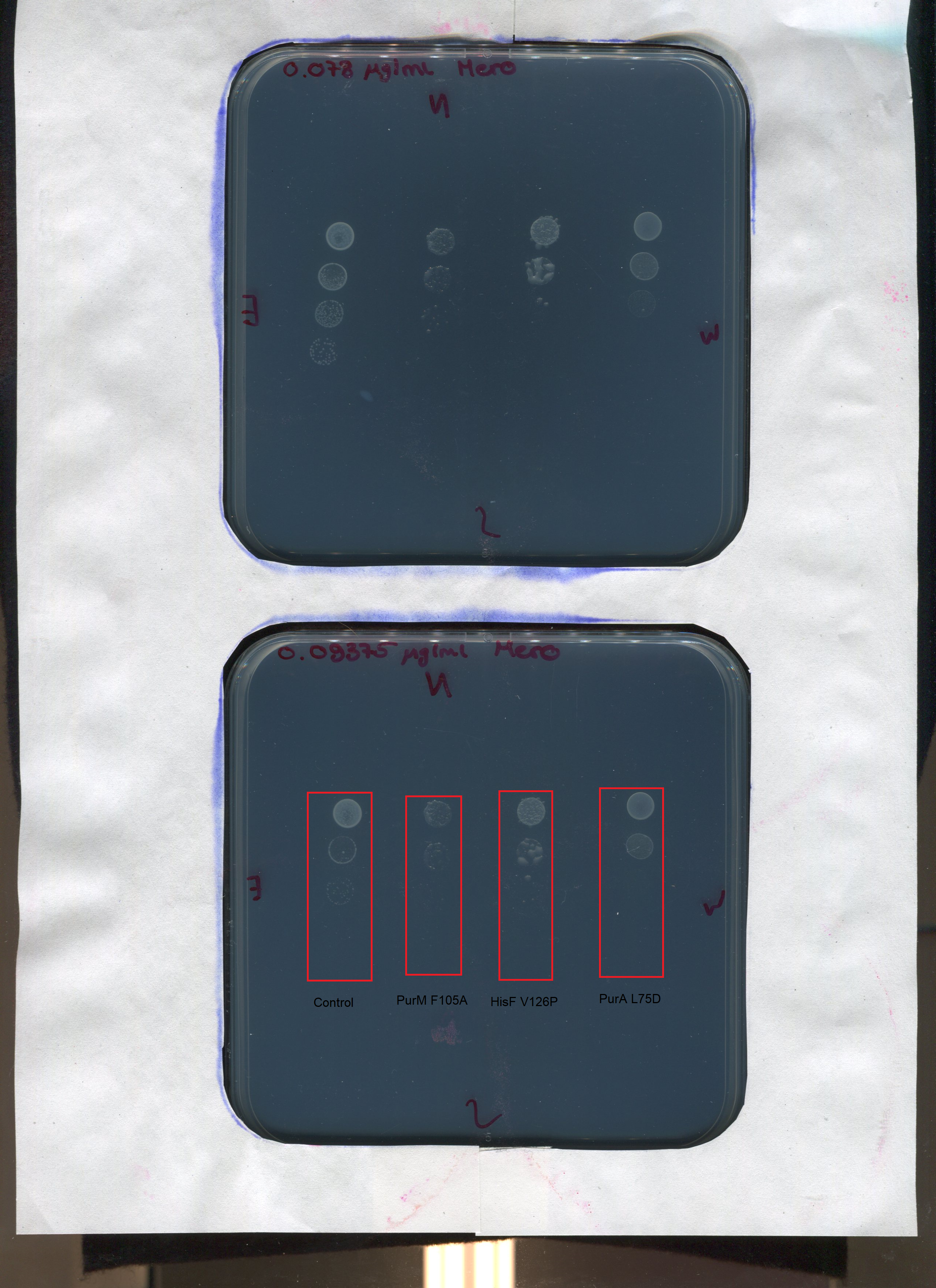

Supplement: Supplementary file 17 — Figure S3 Source Data [file 44320_2024_84_MOESM17_ESM.zip › SD_figS3/S3B/Mero 0.07 + 0.09.tif]

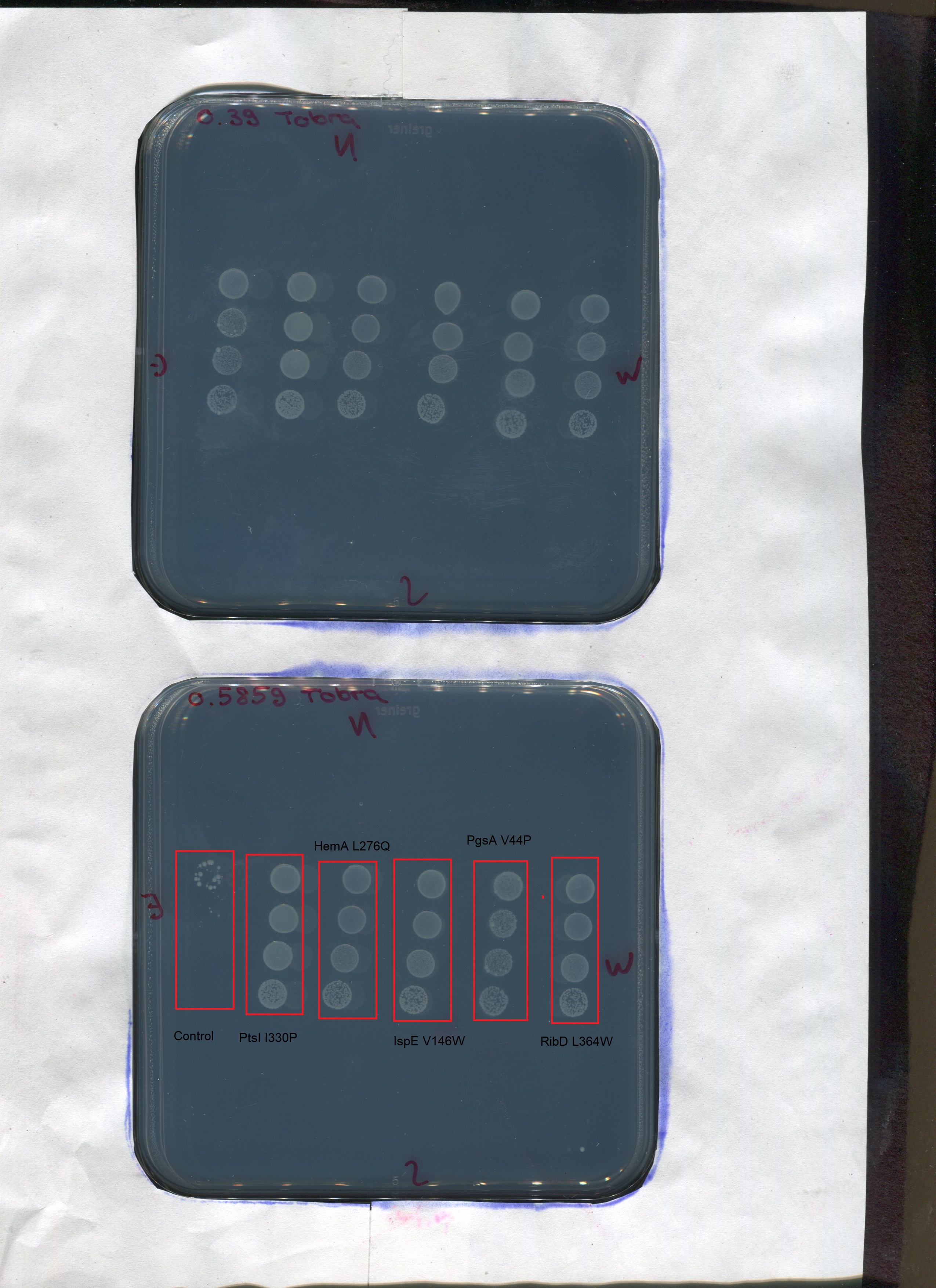

Supplement: Supplementary file 17 — Figure S3 Source Data [file 44320_2024_84_MOESM17_ESM.zip › SD_figS3/S3C/0.39 and 0.58.jpg]

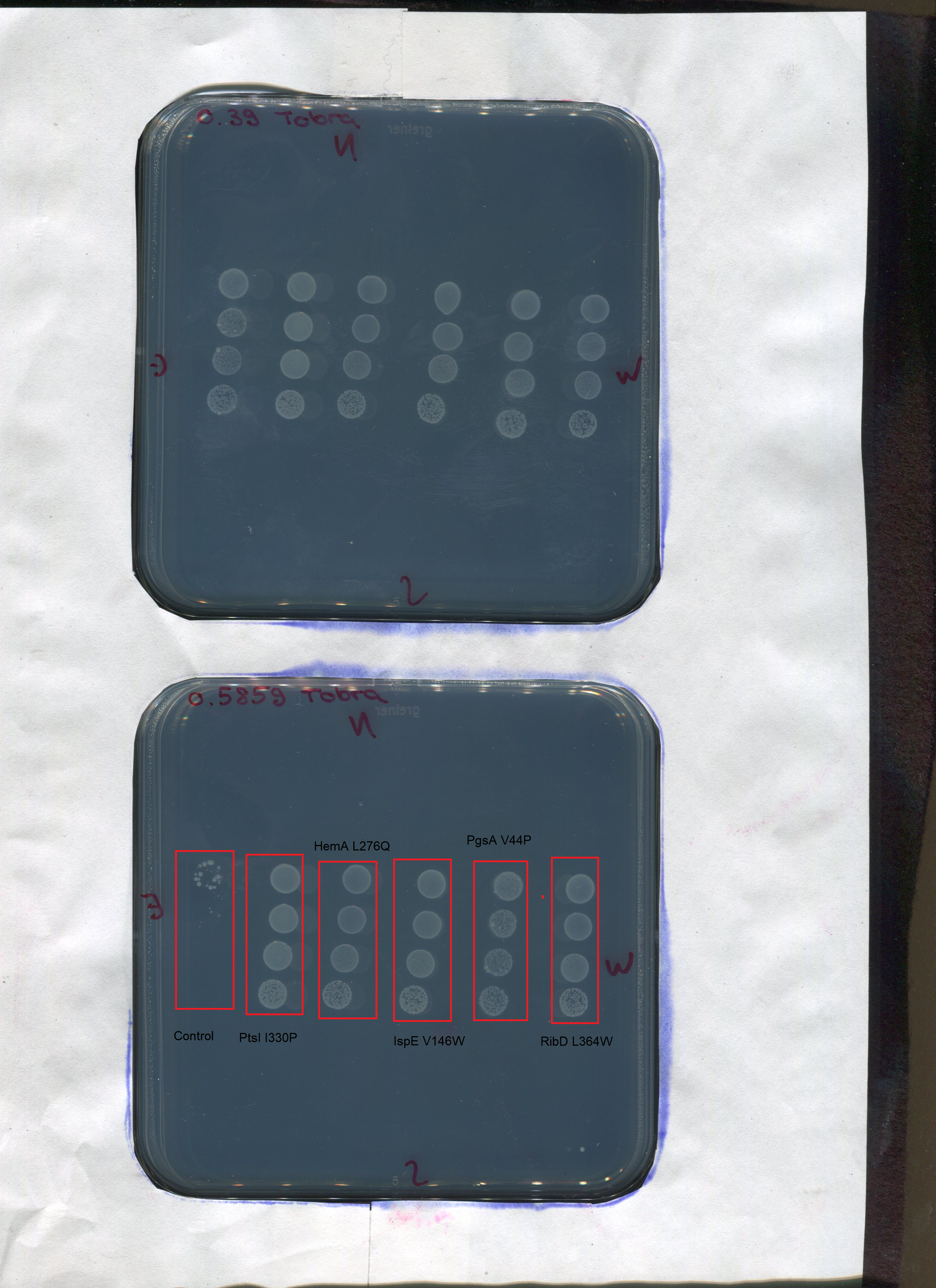

Supplement: Supplementary file 17 — Figure S3 Source Data [file 44320_2024_84_MOESM17_ESM.zip › SD_figS3/S3C/0.39 and 0.58.tif]

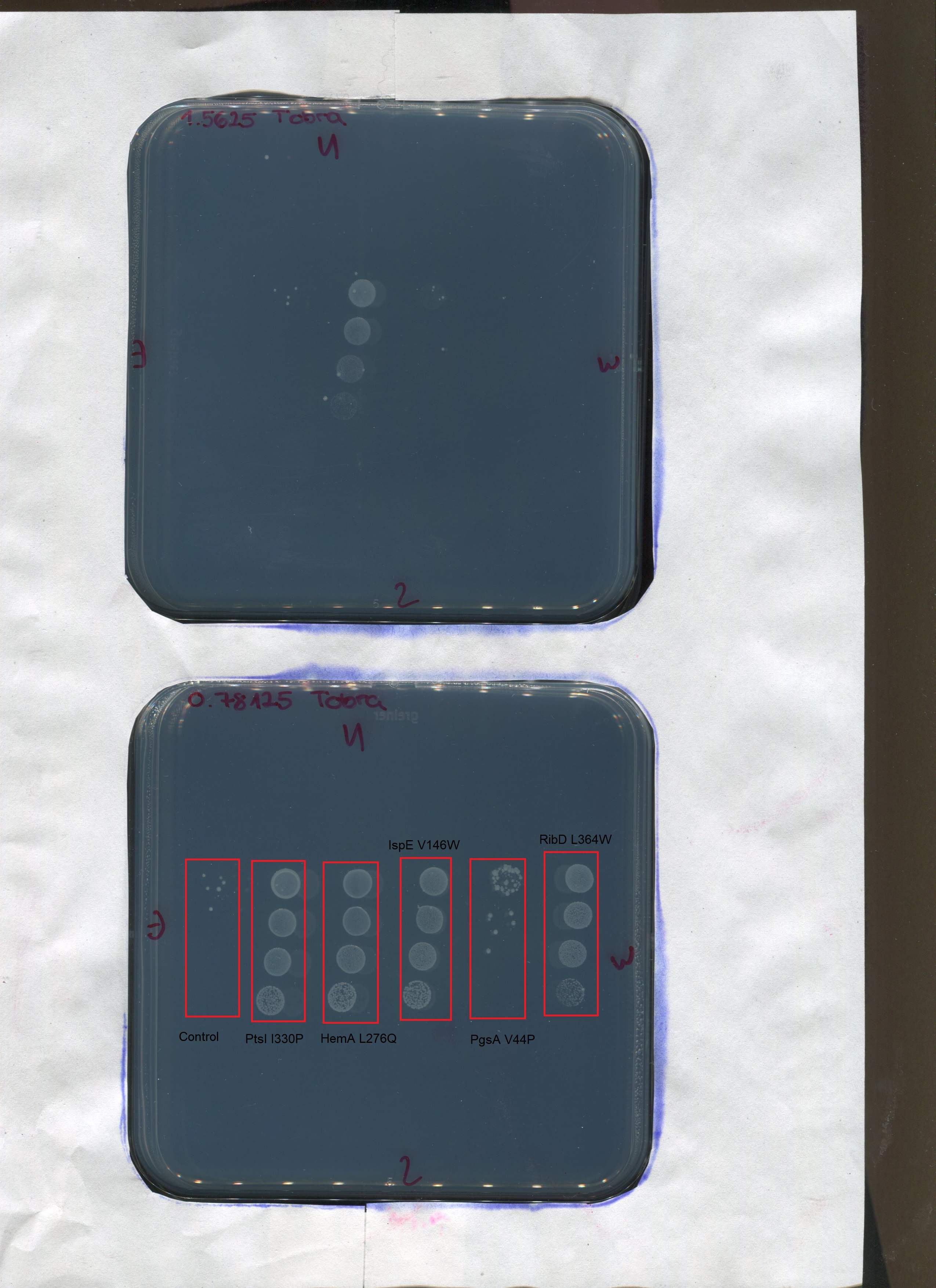

Supplement: Supplementary file 17 — Figure S3 Source Data [file 44320_2024_84_MOESM17_ESM.zip › SD_figS3/S3C/0.7 and 1.5.jpg]

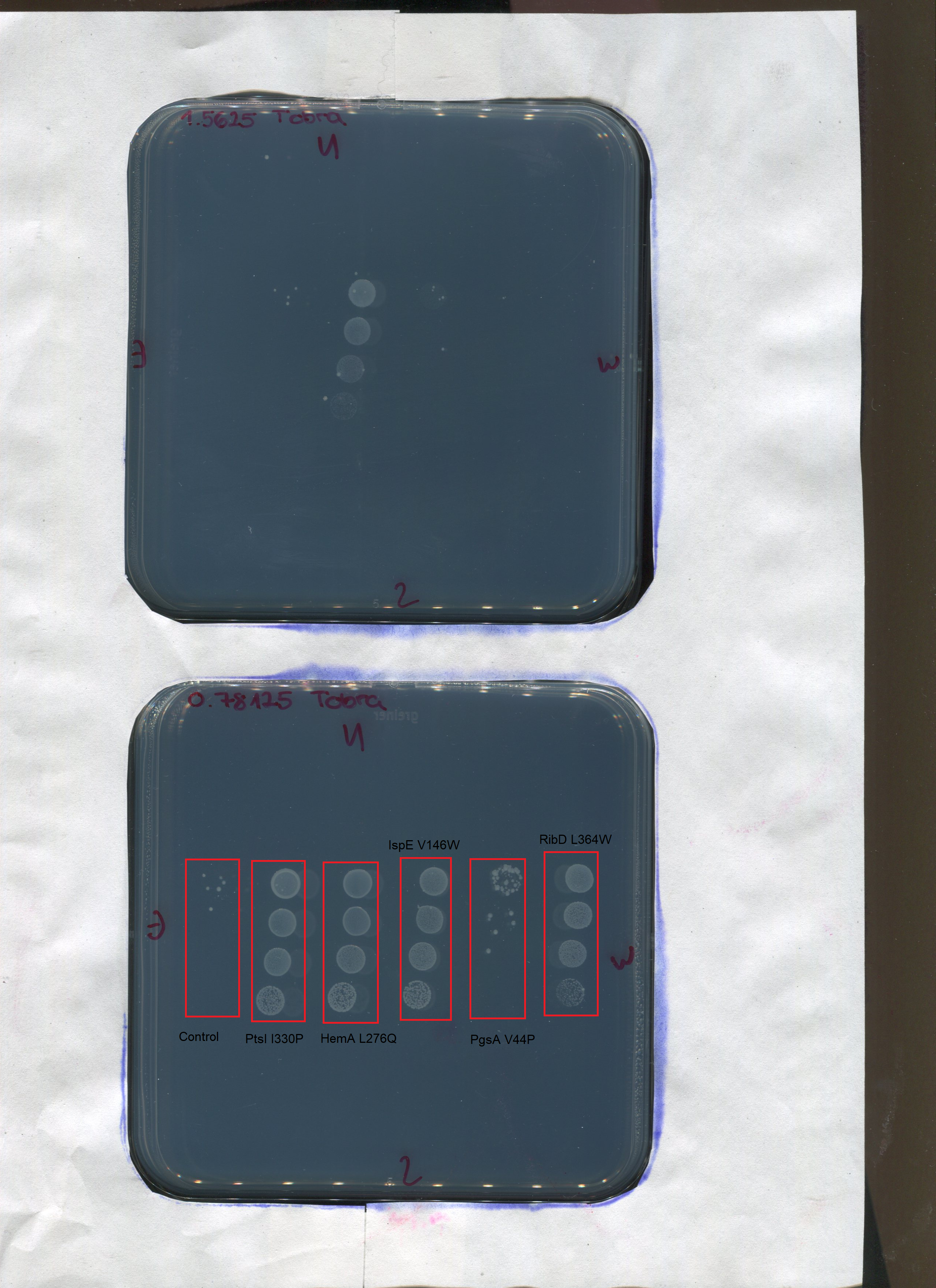

Supplement: Supplementary file 17 — Figure S3 Source Data [file 44320_2024_84_MOESM17_ESM.zip › SD_figS3/S3C/0.7 and 1.5.tif]

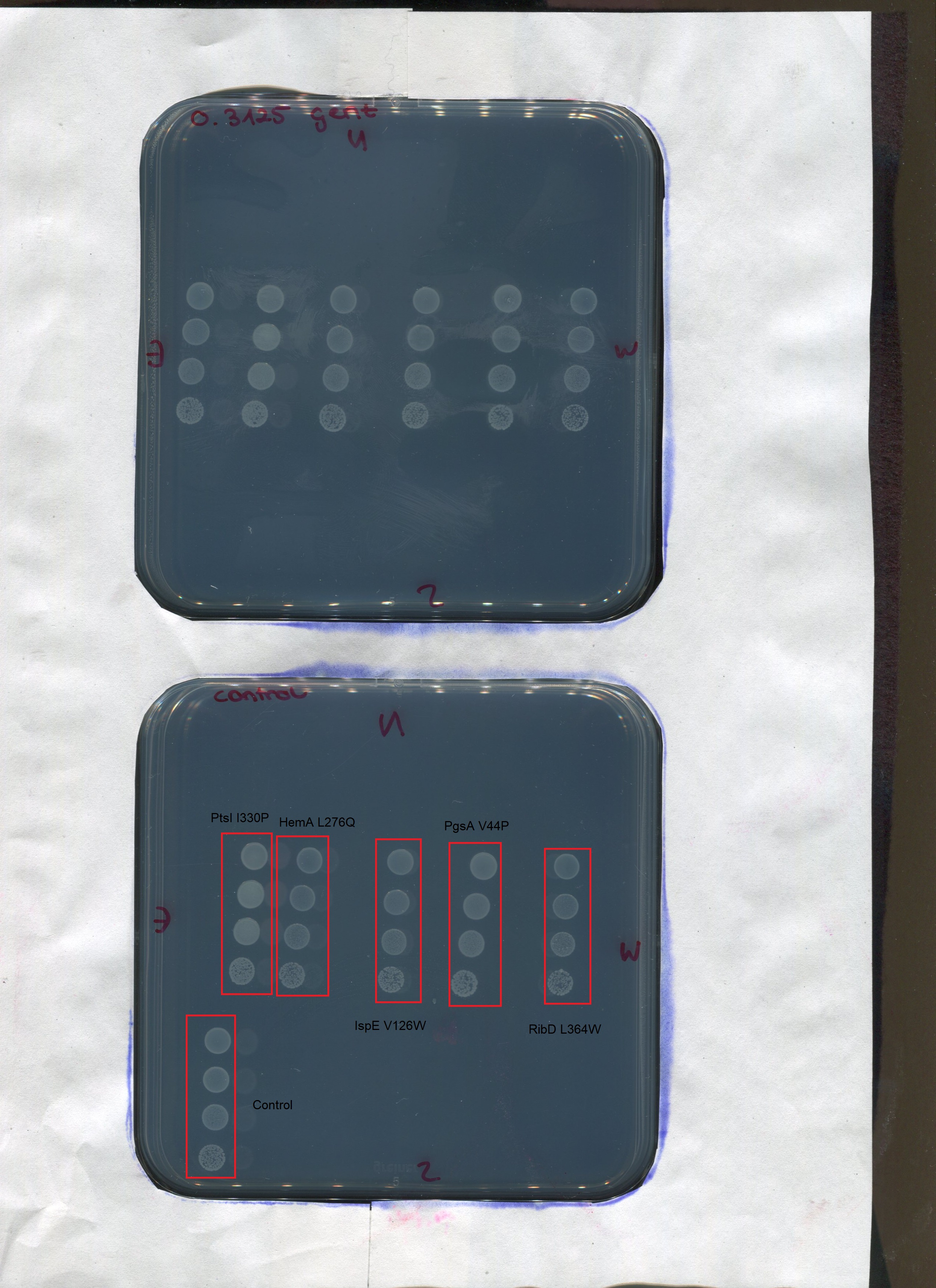

Supplement: Supplementary file 17 — Figure S3 Source Data [file 44320_2024_84_MOESM17_ESM.zip › SD_figS3/S3C/0.jpg]

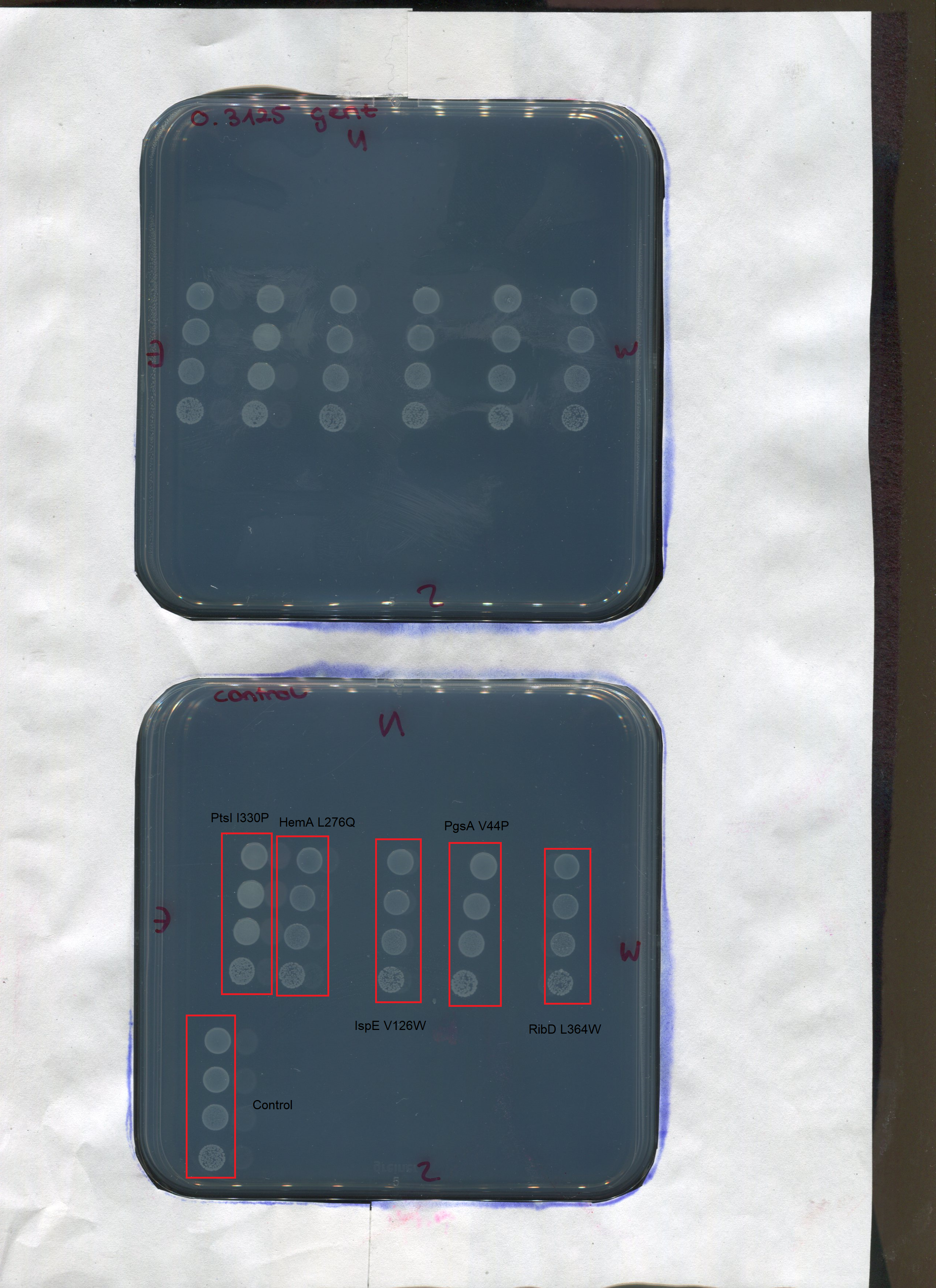

Supplement: Supplementary file 17 — Figure S3 Source Data [file 44320_2024_84_MOESM17_ESM.zip › SD_figS3/S3C/0.tif]

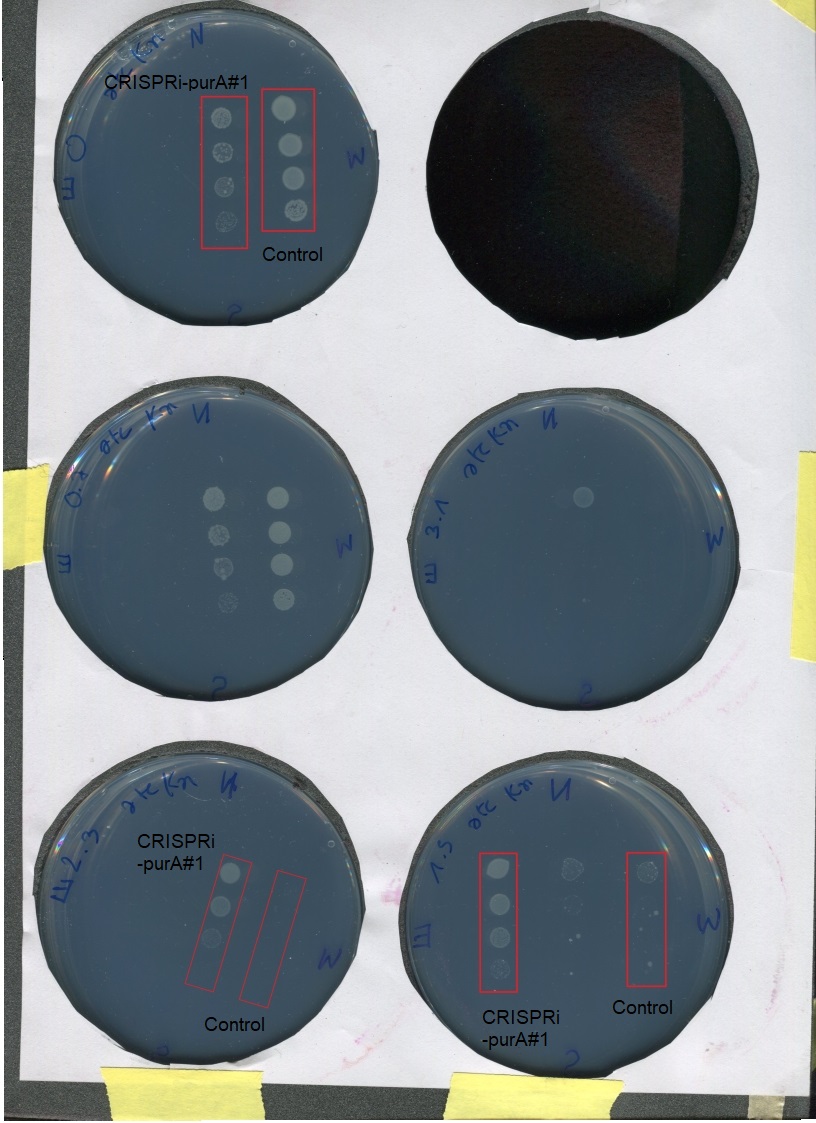

Supplement: Supplementary file 18 — Figure S7 Source Data [file 44320_2024_84_MOESM18_ESM.zip › SD_figS6/All concentrations.jpg]

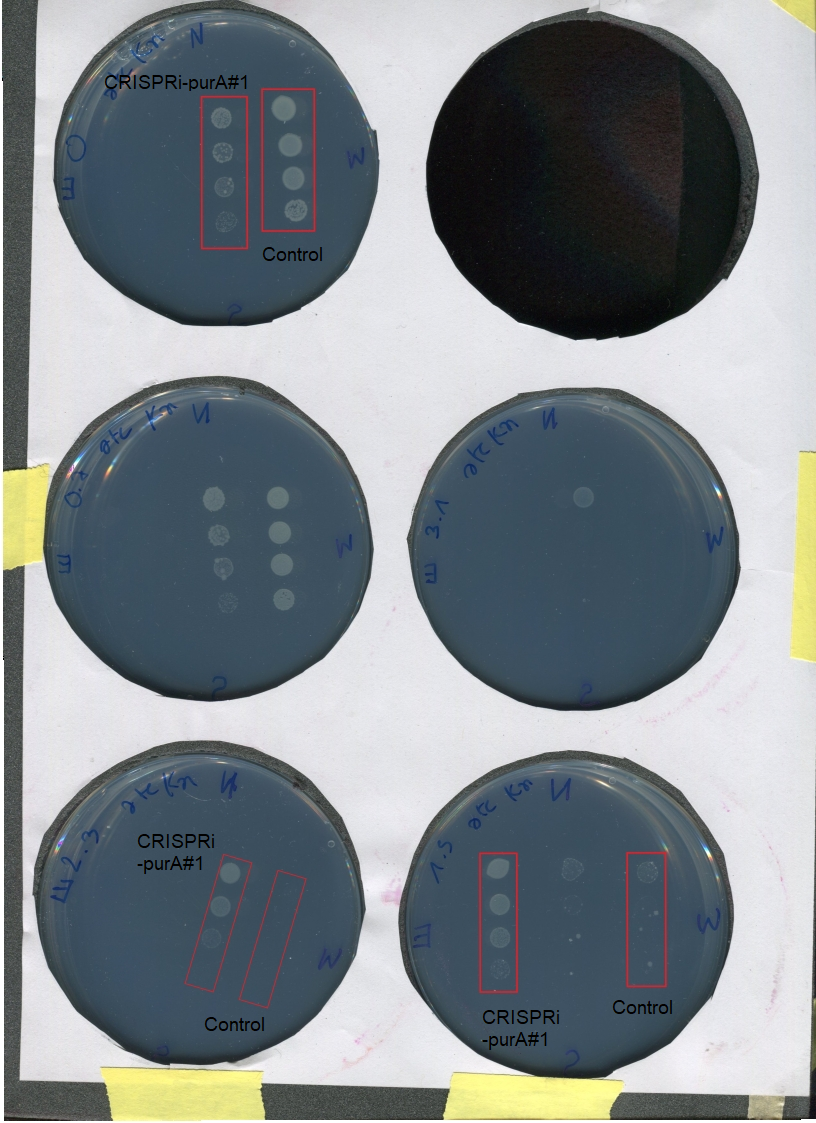

Supplement: Supplementary file 18 — Figure S7 Source Data [file 44320_2024_84_MOESM18_ESM.zip › SD_figS6/All concentrations.tif]

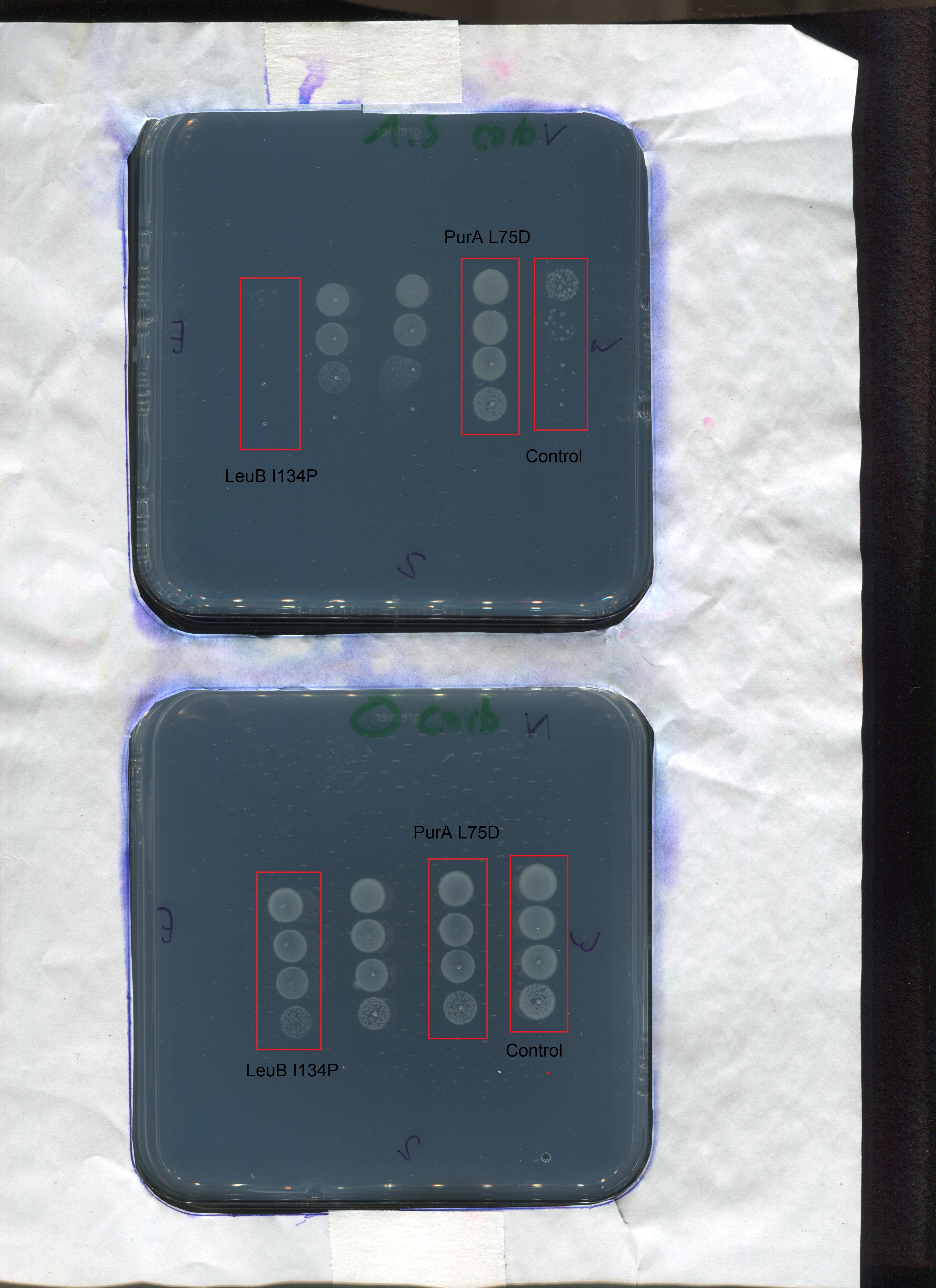

Supplement: Supplementary file 19 — Figure S9 Source Data [file 44320_2024_84_MOESM19_ESM.zip › SD_figS8/0 and 1.5.jpg]

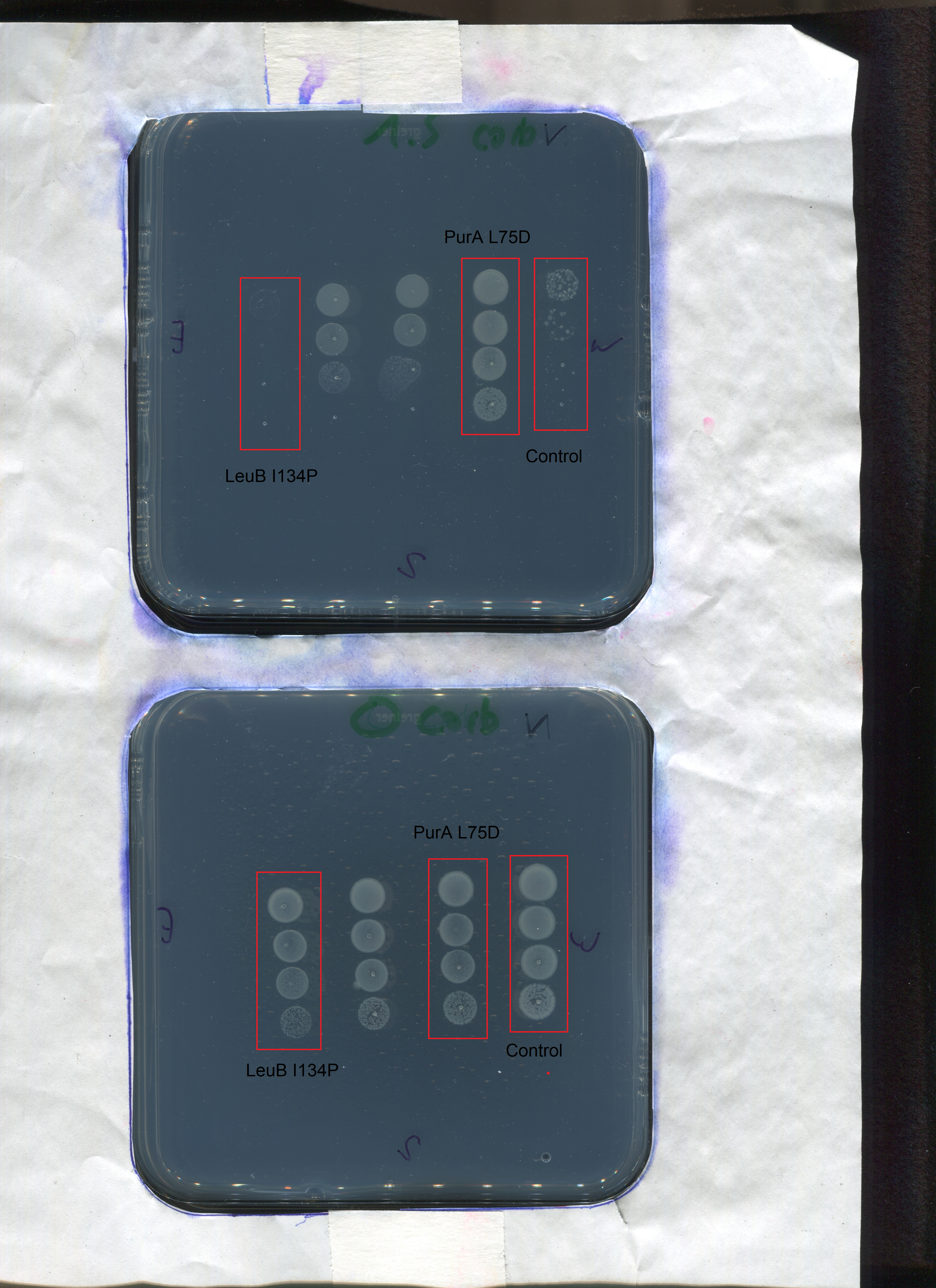

Supplement: Supplementary file 19 — Figure S9 Source Data [file 44320_2024_84_MOESM19_ESM.zip › SD_figS8/0 and 1.5.tif]

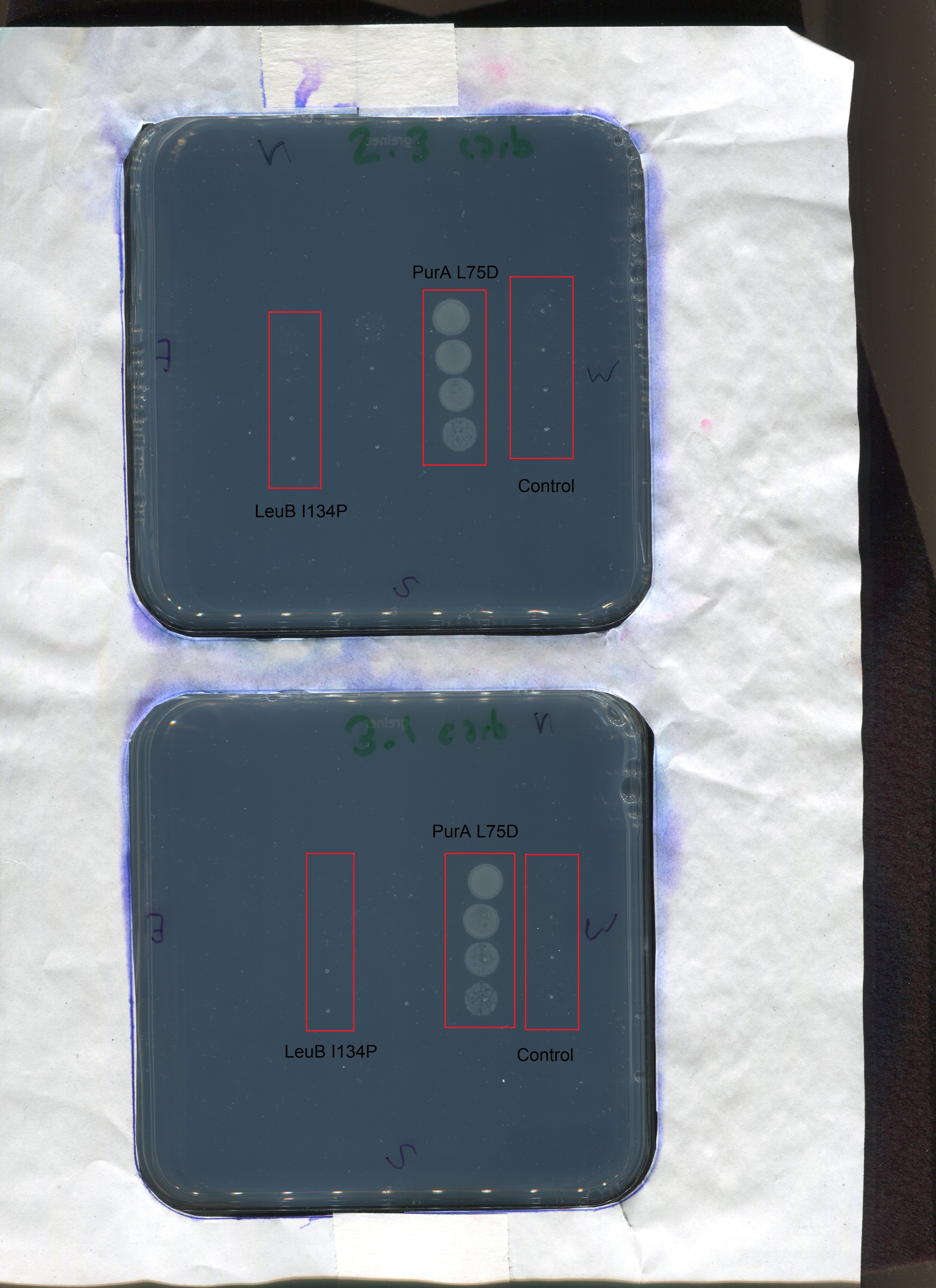

Supplement: Supplementary file 19 — Figure S9 Source Data [file 44320_2024_84_MOESM19_ESM.zip › SD_figS8/2.3 and 3.1.jpg]

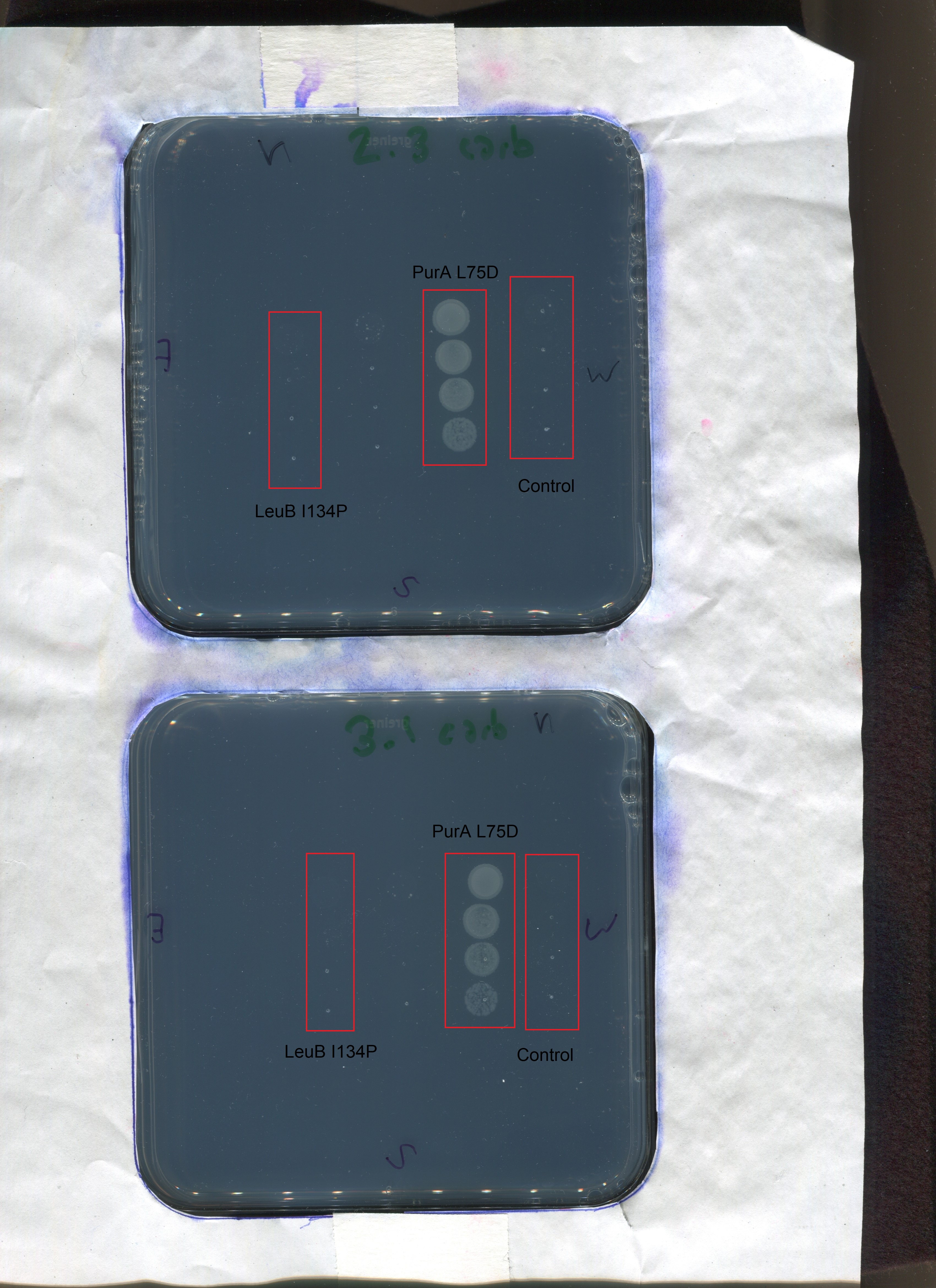

Supplement: Supplementary file 19 — Figure S9 Source Data [file 44320_2024_84_MOESM19_ESM.zip › SD_figS8/2.3 and 3.1.tif]

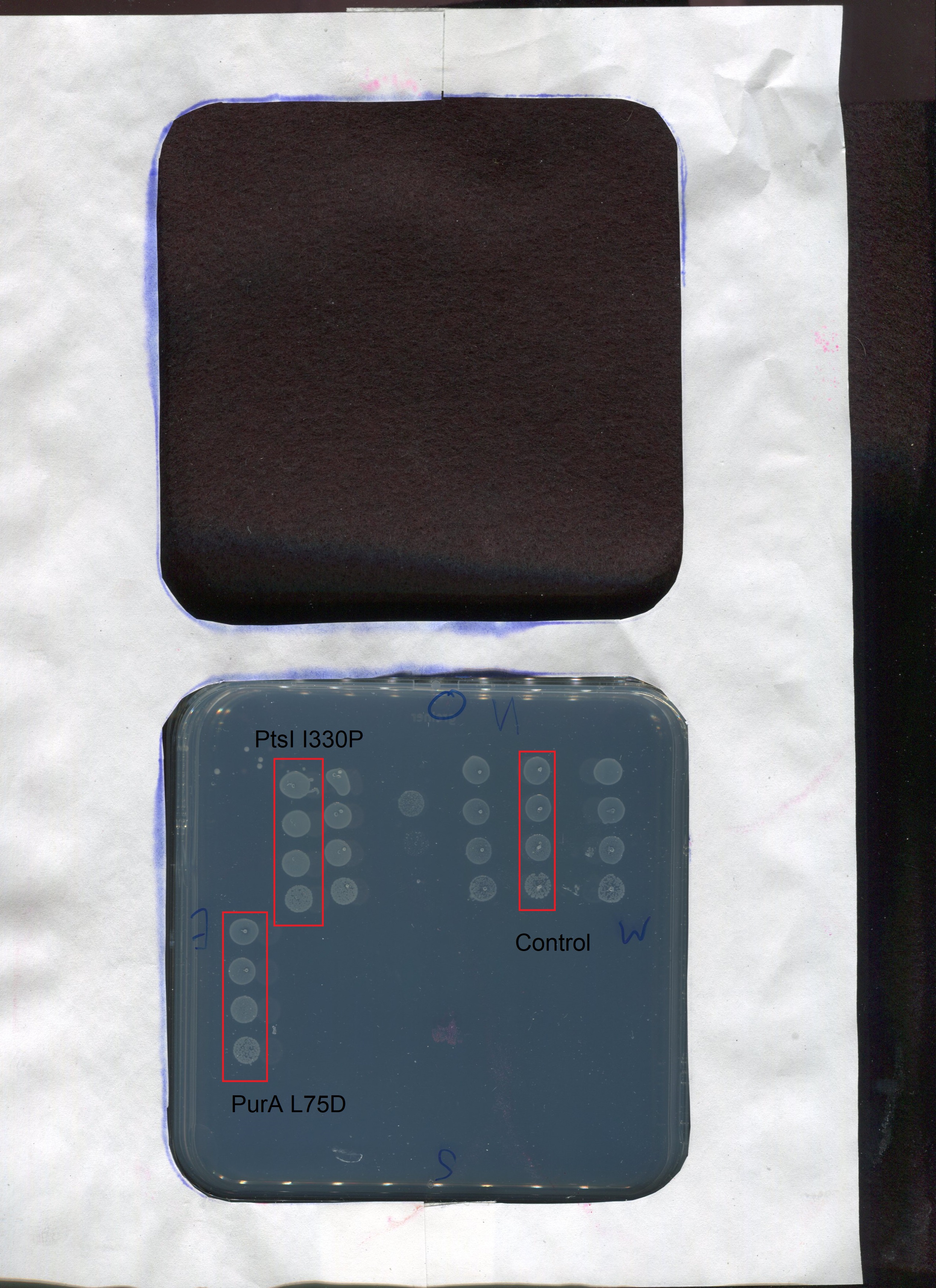

Supplement: Supplementary file 20 — Figure S10 Source Data [file 44320_2024_84_MOESM20_ESM.zip › SD_figS9/S9A/0.jpg]

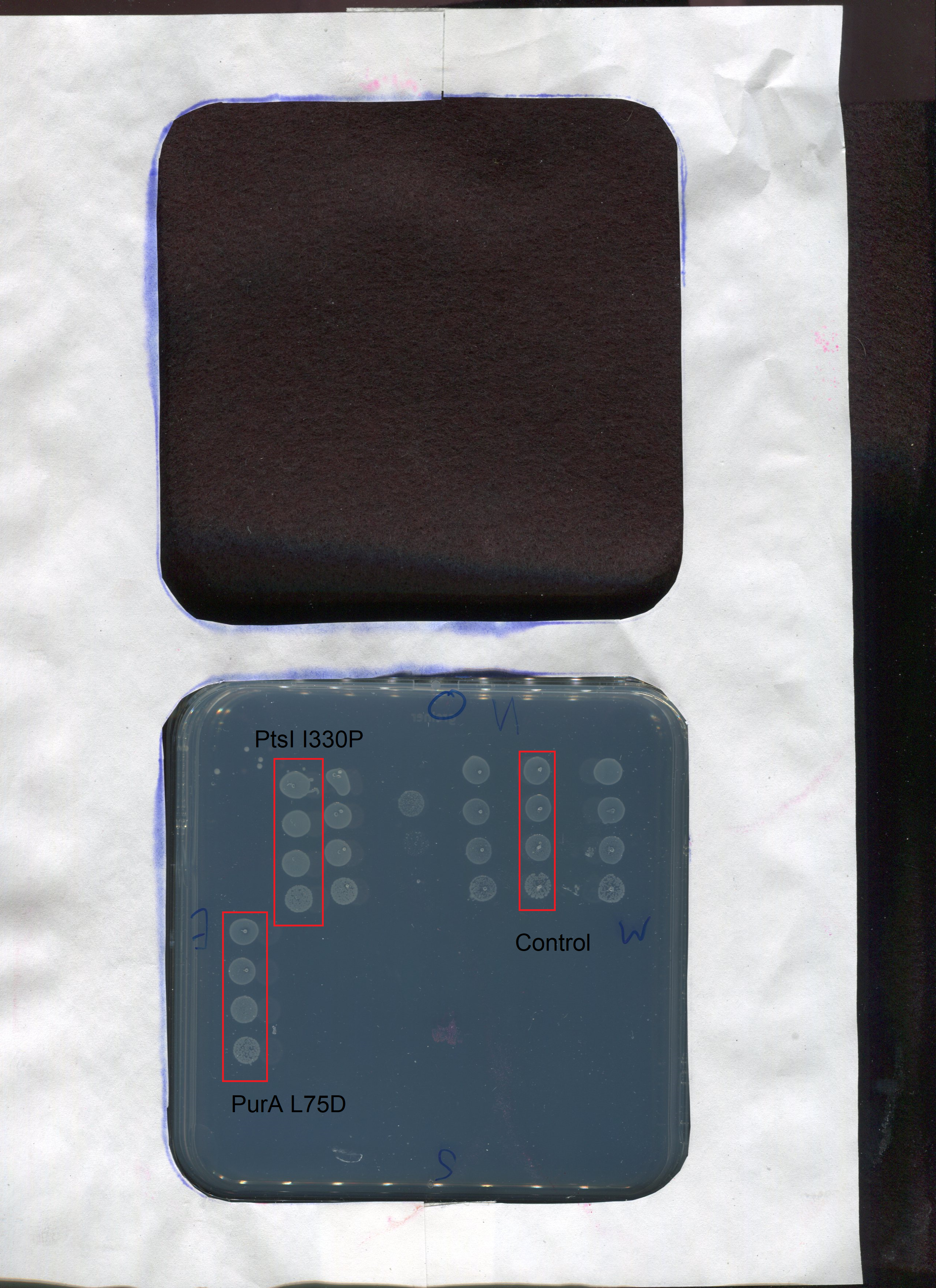

Supplement: Supplementary file 20 — Figure S10 Source Data [file 44320_2024_84_MOESM20_ESM.zip › SD_figS9/S9A/0.tif]

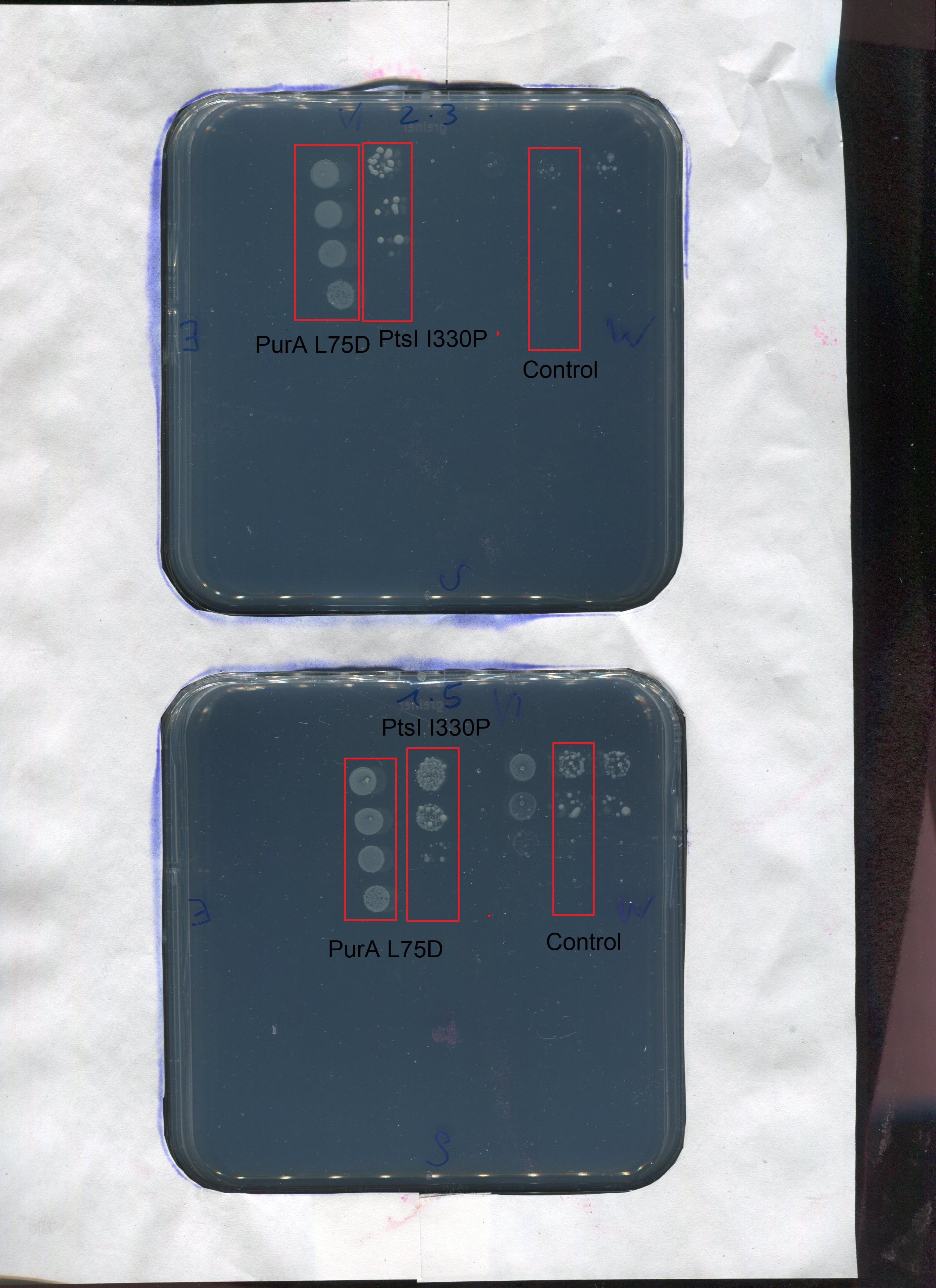

Supplement: Supplementary file 20 — Figure S10 Source Data [file 44320_2024_84_MOESM20_ESM.zip › SD_figS9/S9A/2.3 and 1.5.jpg]

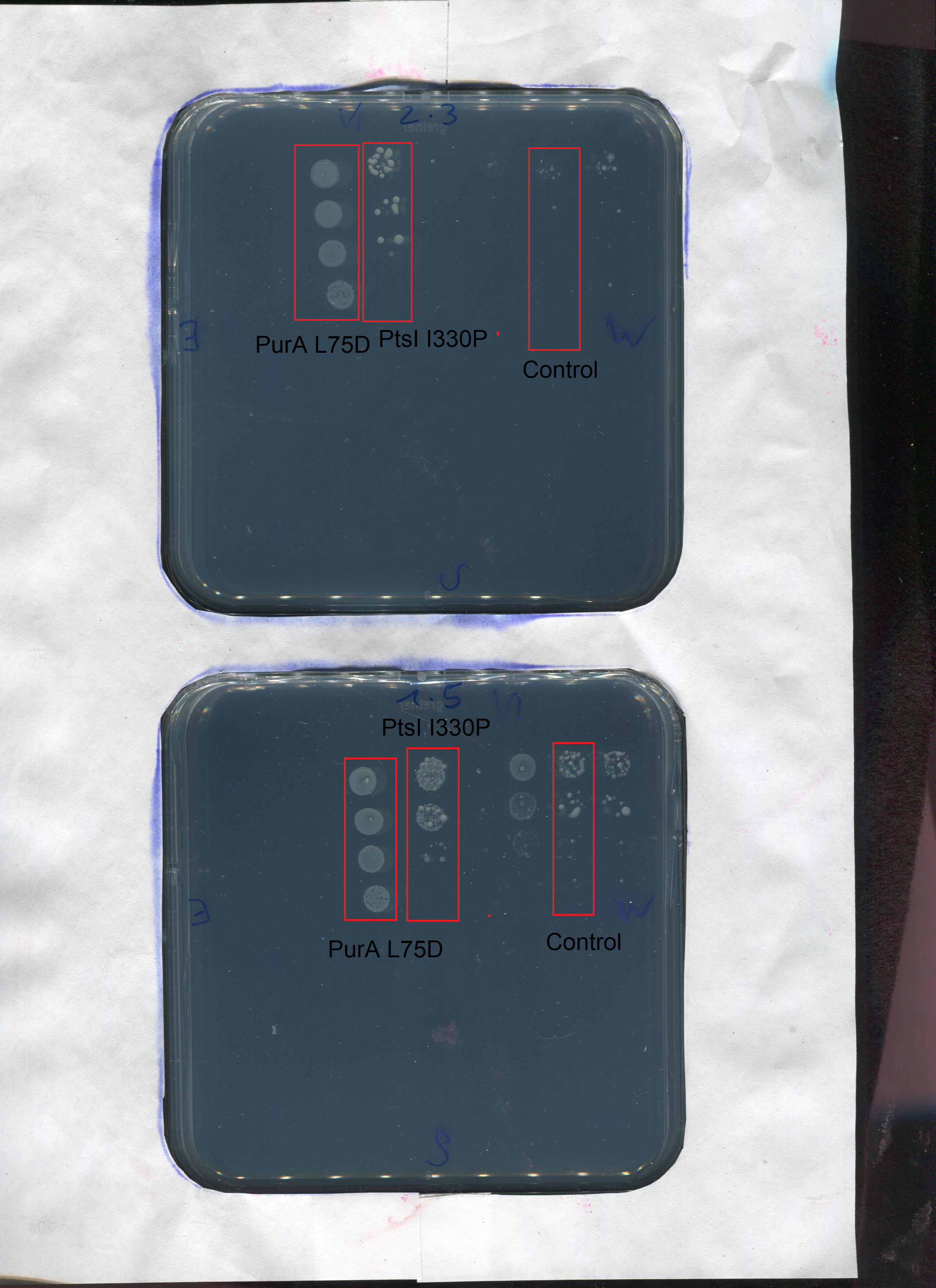

Supplement: Supplementary file 20 — Figure S10 Source Data [file 44320_2024_84_MOESM20_ESM.zip › SD_figS9/S9A/2.3 and 1.5.tif]

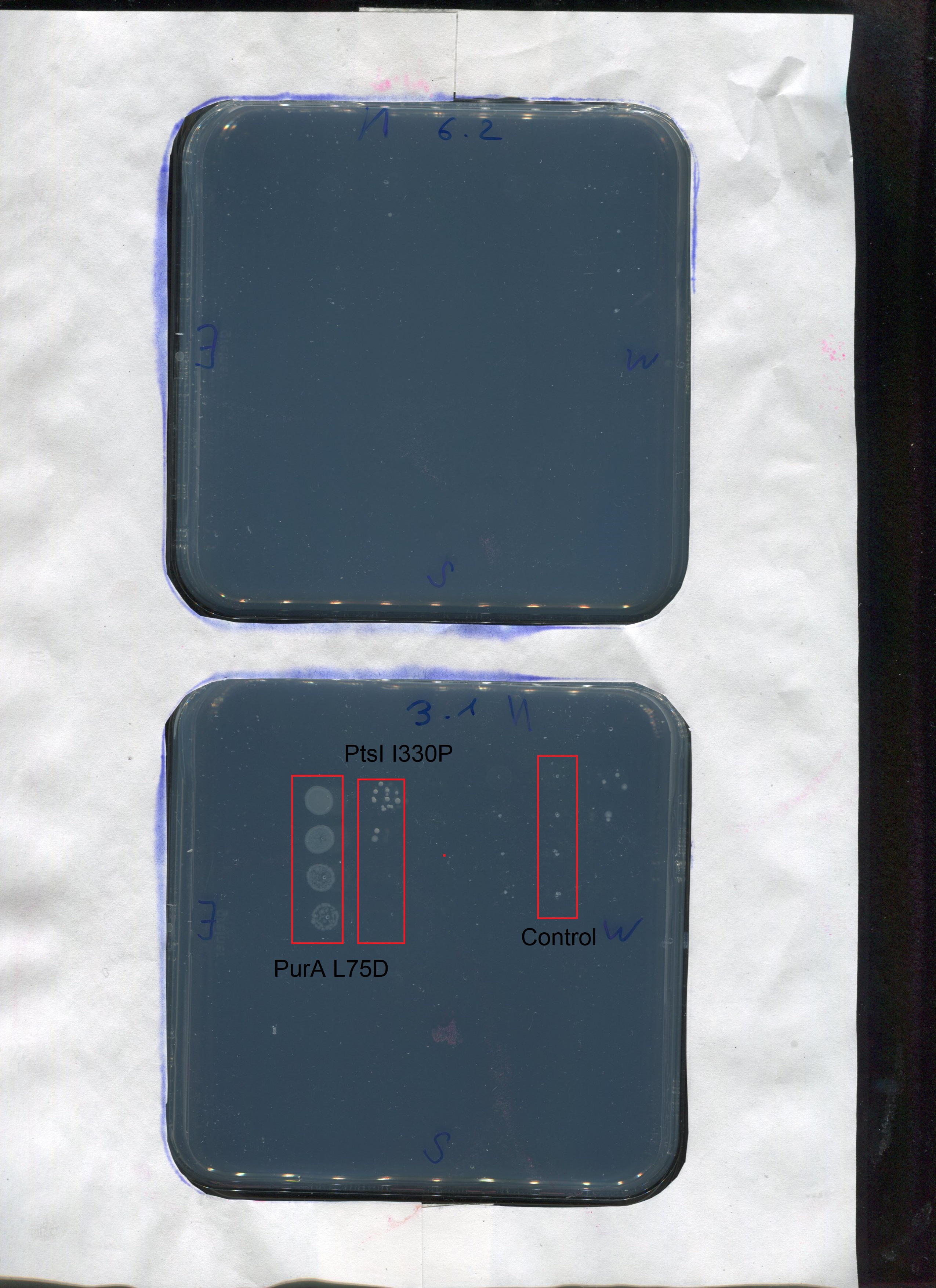

Supplement: Supplementary file 20 — Figure S10 Source Data [file 44320_2024_84_MOESM20_ESM.zip › SD_figS9/S9A/3.1.jpg]

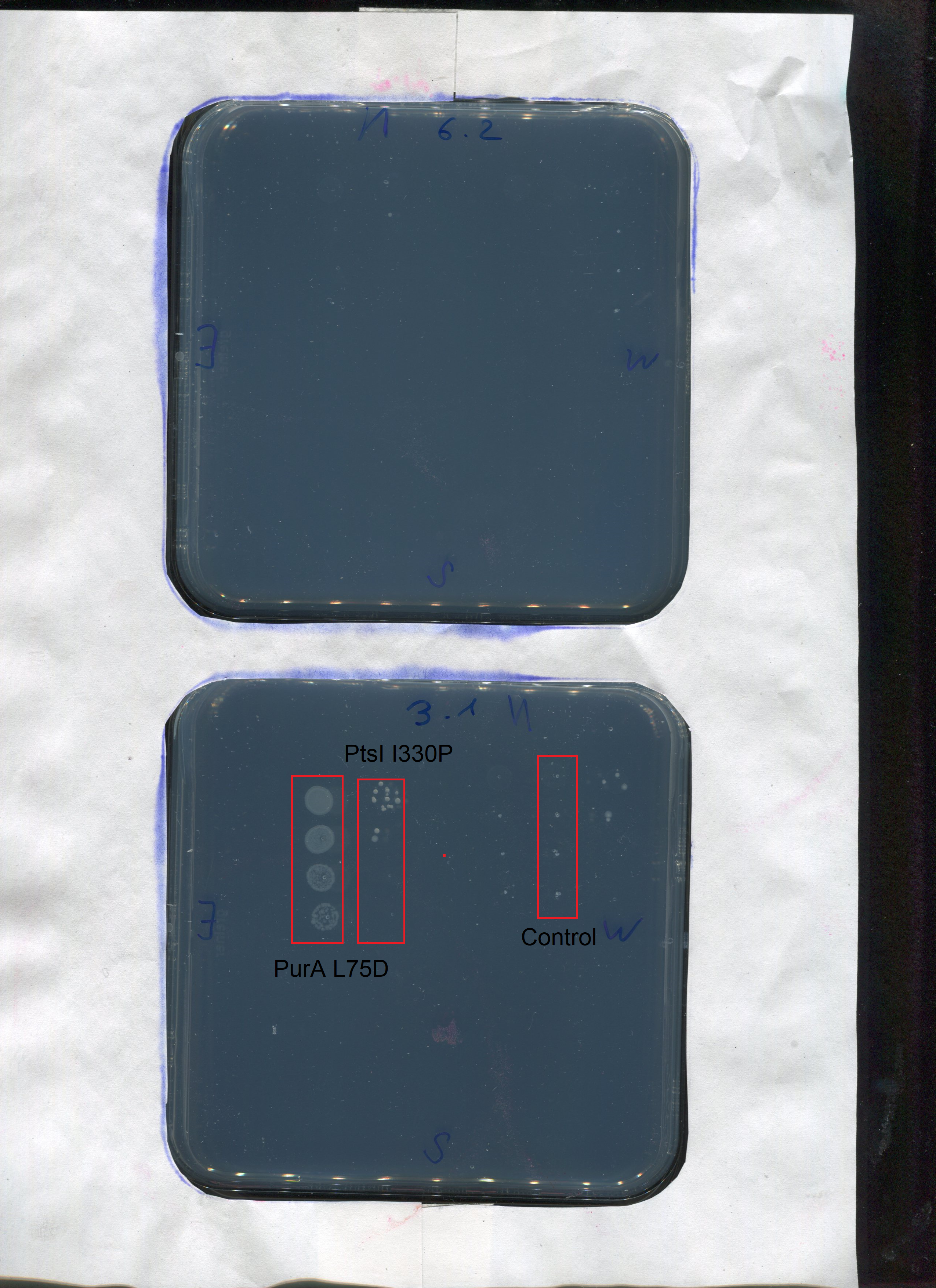

Supplement: Supplementary file 20 — Figure S10 Source Data [file 44320_2024_84_MOESM20_ESM.zip › SD_figS9/S9A/3.1.tif]

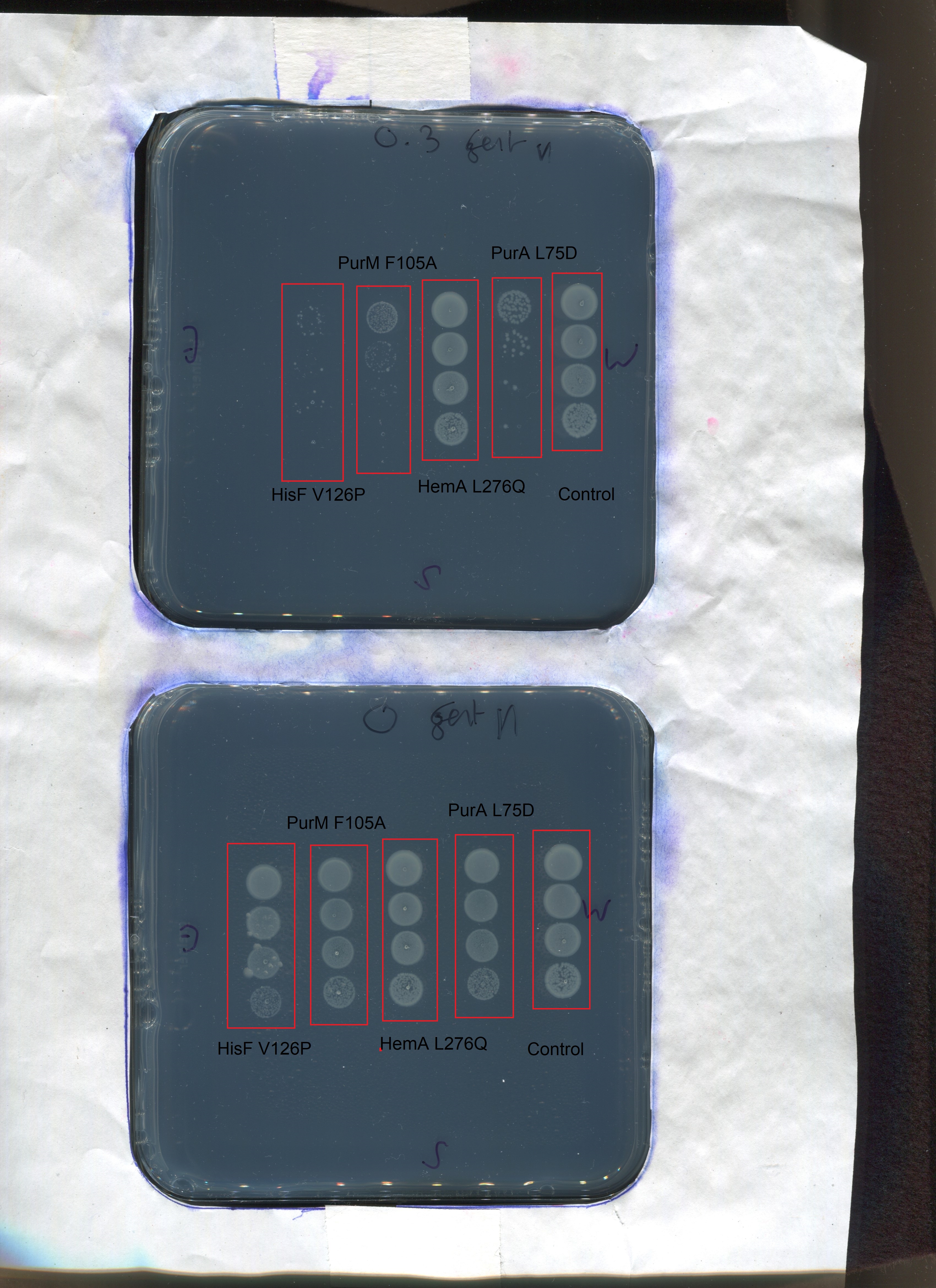

Supplement: Supplementary file 20 — Figure S10 Source Data [file 44320_2024_84_MOESM20_ESM.zip › SD_figS9/S9B/0 and 0.3.jpg]

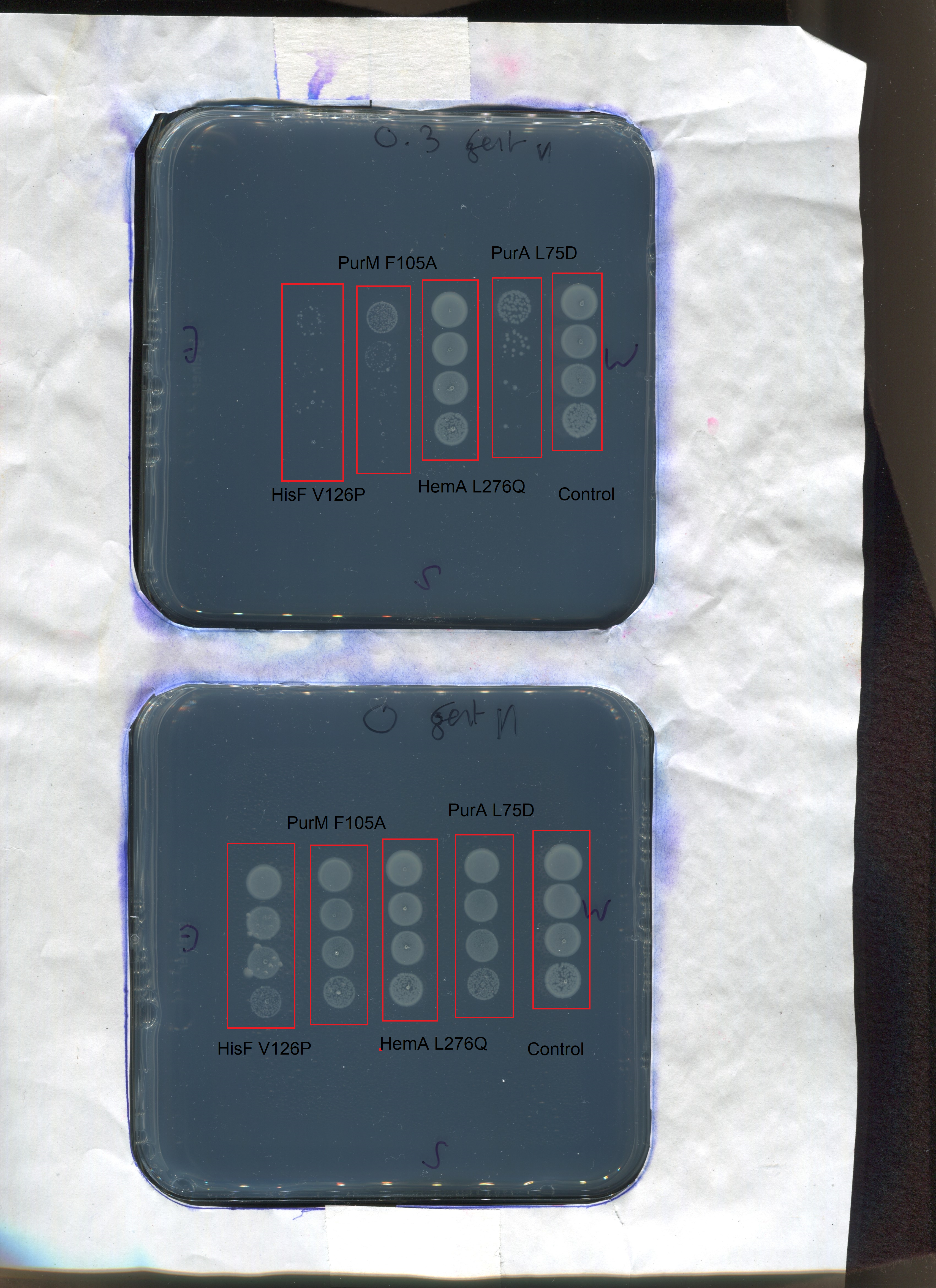

Supplement: Supplementary file 20 — Figure S10 Source Data [file 44320_2024_84_MOESM20_ESM.zip › SD_figS9/S9B/0 and 0.3.tif]

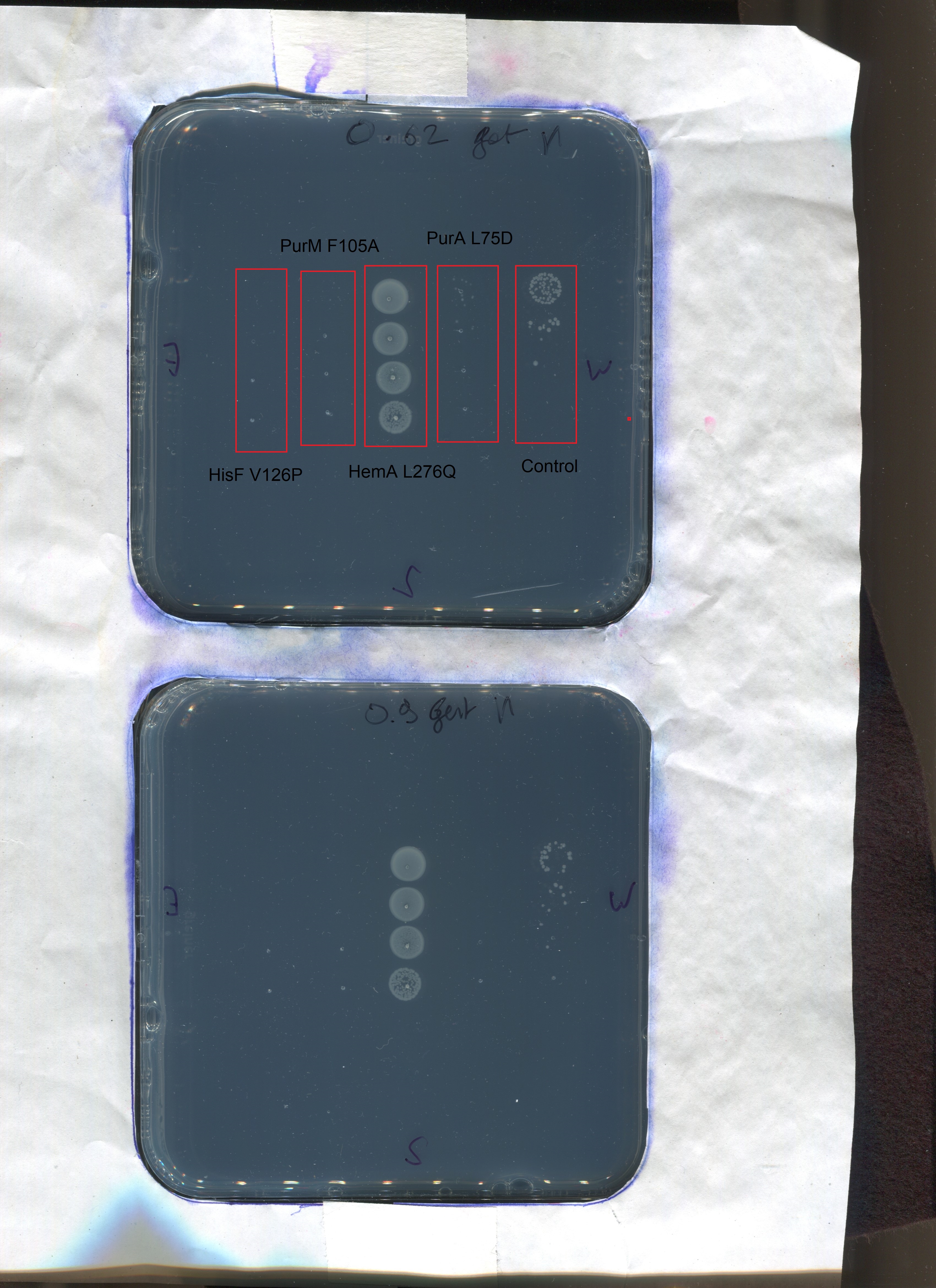

Supplement: Supplementary file 20 — Figure S10 Source Data [file 44320_2024_84_MOESM20_ESM.zip › SD_figS9/S9B/0.6.jpg]

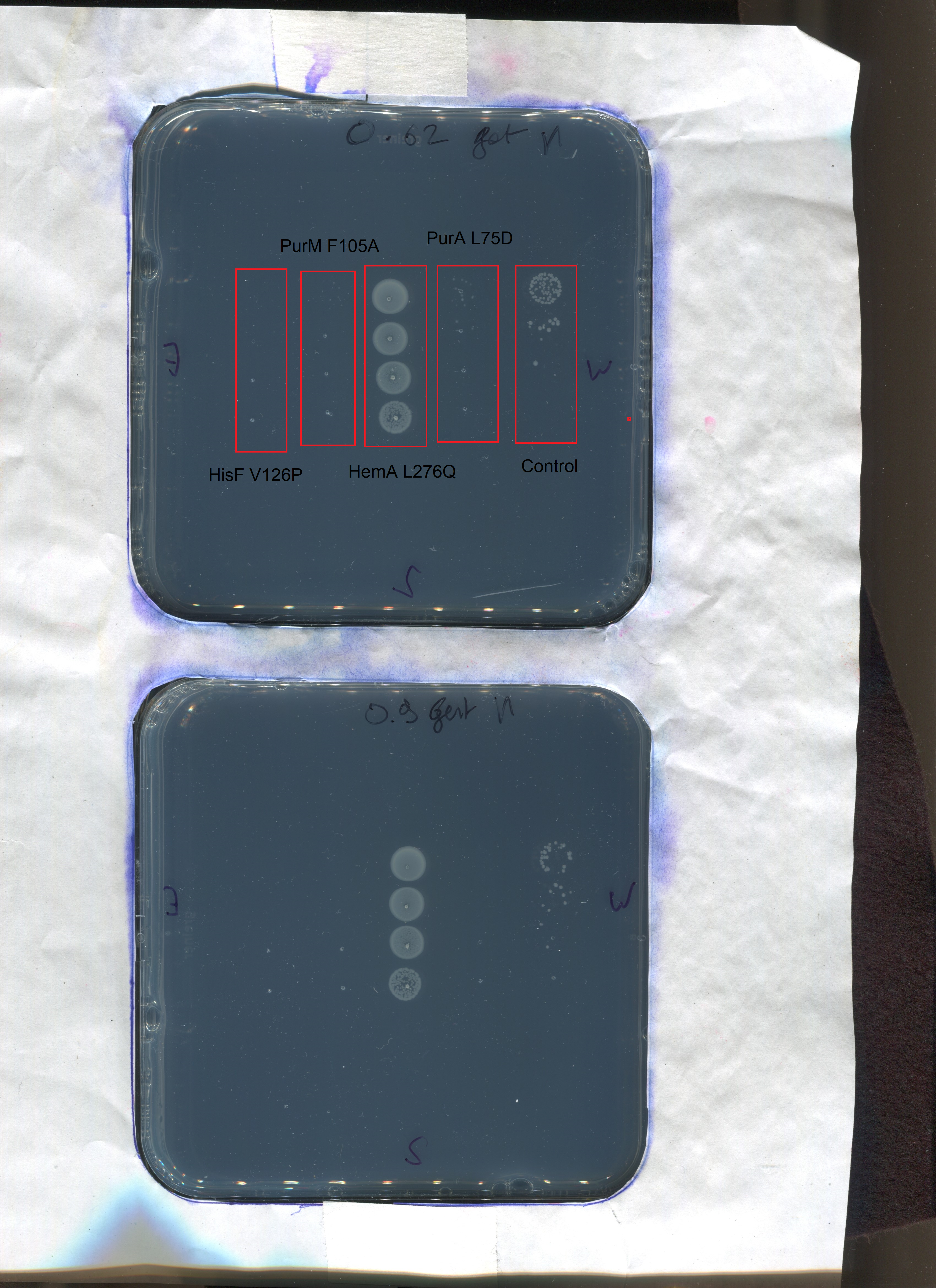

Supplement: Supplementary file 20 — Figure S10 Source Data [file 44320_2024_84_MOESM20_ESM.zip › SD_figS9/S9B/0.6.tif]
