# Supplementary material for: Metabolic mutations reduce antibiotic susceptibility of E. coli by pathway-specific bottlenecks
Source: Mol Syst Biol. 2025 Jan 2;21(3):274–93. doi: 10.1038/s44320-024-00084-z (PMC11876631; doi:10.1038/s44320-024-00084-z)
Supplement: Supplementary file 21 — Expanded View Figures [file 44320_2024_84_MOESM21_ESM.pdf]

Expanded View Figures

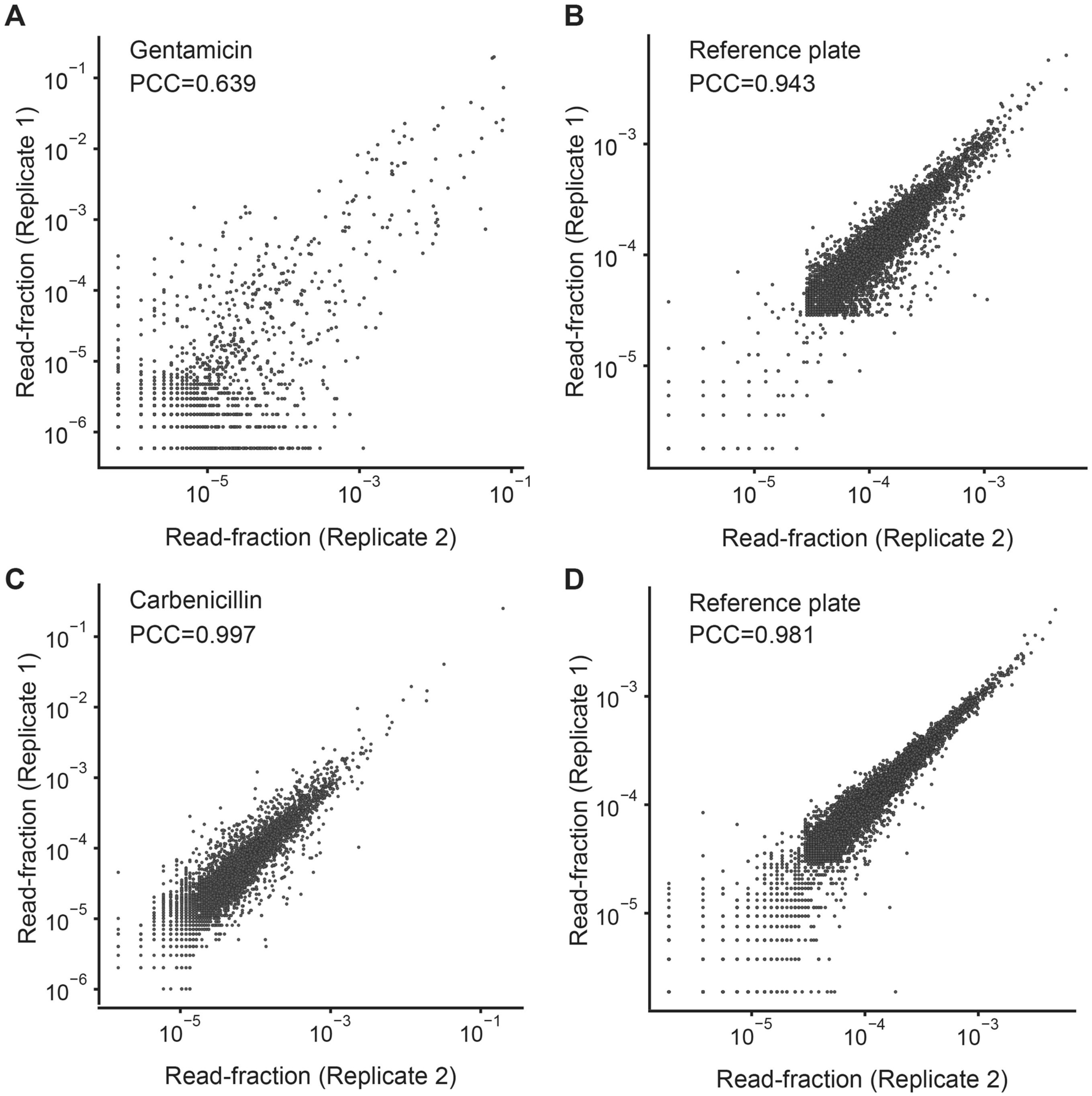

Figure EV1. Read fractions of barcodes of mutants in the CRISPR library.

(**A**, **B**) are barcode read fractions from the gentamicin screen. (**C**, **D**) are barcode read fractions from the carbenicillin screen. The Pearson correlation coefficient (PCC) is shown for the two replicates.

© The Author(s)

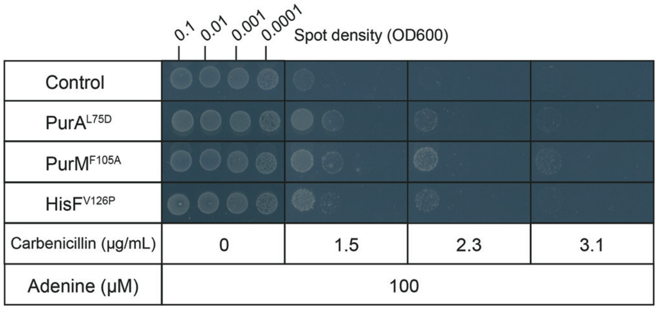

Figure EV2. Agar dilution assay with the control strain and three purine mutants (HisF^{V126P}, PurM^{F105A} and PurA^{L75D}).

Each strain was spotted on agar plates with minimal glucose medium supplemented with 100 μM adenine and increasing concentrations of carbenicillin (MIC = 1.5 μg/mL). Plates were incubated 48 h. Shown is one of $n = 2$ replicates. Spot assays were performed on the same plate per concentration, and scans of plates with different concentrations were assembled into a single figure using Adobe Illustrator.

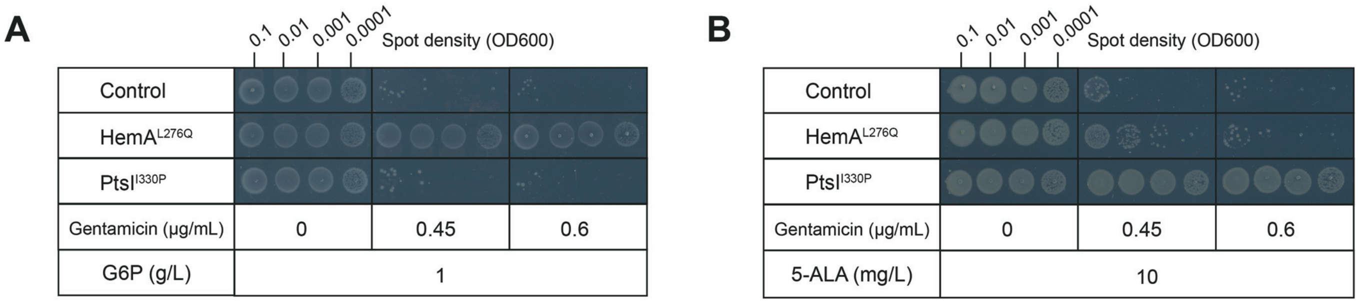

Figure EV3. Agar dilution assay with the control strain, the HemA^{L276Q} strain and the PtsI^{I330P} strain.

(**A**) Each strain was spotted on agar plates with minimal medium containing glucose-6-phosphate (G6P) instead of glucose as carbon source, and with increasing concentrations of gentamicin (MIC = 0.45 μg/mL). (**B**) same as (**A**) but with glucose as carbon source and supplementation of 5-aminolevulinic acid (5-ALA). Plates were incubated 48 h. Spot assays were performed on the same plate per concentration, and scans of plates with different concentrations were assembled into a single figure using Adobe Illustrator.

© The Author(s)

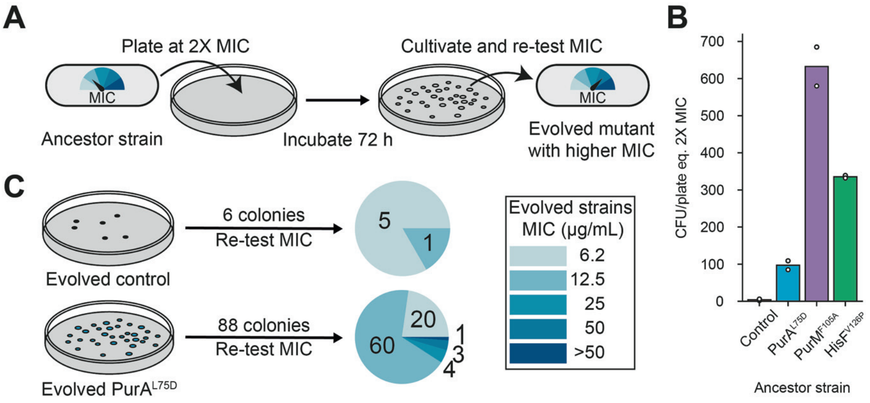

Figure EV4. Evolution of higher carbenicillin resistance.

(**A**) Schematic of the experimental workflow to evolve higher carbenicillin resistance. (**B**) Number of spontaneous mutants that appeared after 3 days incubation of the control strains and the three mutants (HisF^{V126P}, PurM^{F105A} and PurA^{L75D}) at their respective 2× MIC (3.1 μg/mL for the control strain and 6.2 μg/mL for the purine mutants). Bars are means of $n = 2$ distinct samples (dots). (**C**) Pie charts show carbenicillin MIC values of the evolved control strains and the evolved PurA^{L75D} strains. 88 colonies were picked from a plate inoculated with the PurA^{L75D} strain. All 6 colonies on the plate with the control strain were picked. Agar dilution assays on minimal glucose agar were performed to assess the MIC of these strains.

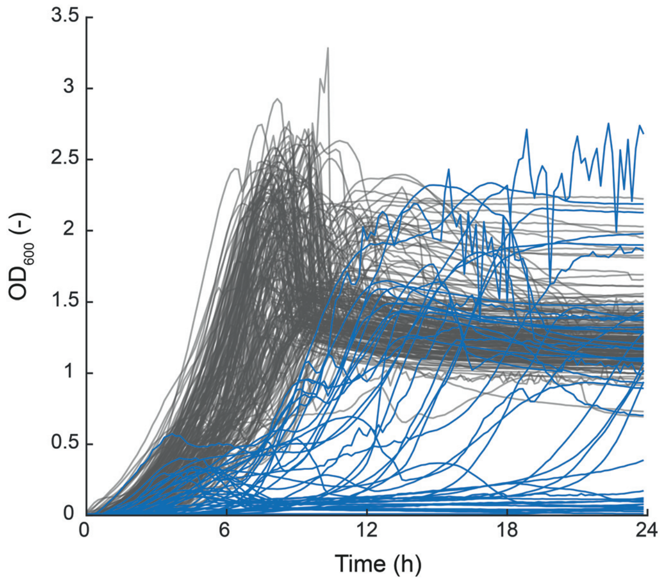

Figure EV5. Growth of 235 clinical *E. coli* isolates on minimal glucose medium.

Curves are the mean of $n = 2$ cultures in 96-well plates. Blue lines show 41 strains with the lowest area under the curve (AUC). These strains were used for metabolome analysis.

 © The Author(s)
